# Pathway control in metallosupramolecular polymerization of a monoalkynylplatinum(ii) terpyridine complex through competitive complex formation†

**DOI:** 10.1039/d4sc06083k

**Published:** 2024-11-06

**Authors:** Minhye Kim, Heekyoung Choi, Minjoo Kim, Seonghan Kim, Seohyeon Yun, Eunji Lee, Jaeheung Cho, Sung Ho Jung, Jong Hwa Jung

**Affiliations:** a Department of Chemistry, Gyeongsang National University Jinju 52828 Korea jonghwa@gnu.ac.kr; b Research Institute of Advanced Materials Chemistry, Gyeongsang National University Jinju 52828 Korea; c Department of Chemistry, Ulsan National Institute of Science and Technology Ulsan 44919 Korea; d Department of Chemistry and Advanced Materials, Gangneung-Wonju National University Gangneung 25457 Korea

## Abstract

Understanding the pathway complexity of supramolecular polymerization in biomimetic systems has been a challenging issue due to its importance in the development of rationally controlled materials and insight into self-assembly in nature. We herein report a kinetic trapping strategy as a new methodology on how to control the pathway of metallosupramolecular polymerization by employing secondary metal ions and/or ligands which form competitive complex species. For this, we proposed monoalkynylplatinum(ii) metalloligand (Pt-L^1^) derived from a bis(amideterpyridine) receptor with one unoccupied terpyridyl terminal as a coordination site for the secondary metal ion (Ag^+^ or Fe^2+^). The inherent pathway complexity intrinsic to the Pt-L^1^-anchored supramolecular polymerization has been modulated through the incorporation of Ag^+^ or Fe^2+^. During the supramolecular polymerization of Pt-L^1^ in the presence of Ag^+^ and Fe^2+^, the added secondary ligand bpy (4,4′-dimethyl-2,2′-bipyridine) or DA18C6 (1,14-diaza-18-crown-6) form complexes as kinetic species, thereby inhibiting spontaneous polymerizations. The supramolecular polymer (SP-I), with a spherical structure composed of Pt-L^1^ in the absence of metal ions as a kinetic product, did not transform into the thermodynamic product, namely supramolecular polymer (SP-III) with a left-handed fiber structure, due to a high energy barrier. However, the supramolecular polymer (SP-II) with a left-handed fiber structure, which was formed by Pt-L^1^ in the presence of AgNO_3_, converted to SP-III upon the addition of NaCl. Additionally, SP-II transformed into supramolecular polymer (SP-IV) upon the addition of Fe(BF_4_)_2_, through an on-pathway process. Both the morphological and emissive characteristics of the resulting supramolecular polymers can be fine-tuned *via* the Pt⋯Pt or Ag⋯Ag interactions as well as through the changes of the coordination geometry depending on the existing Ag^+^ or Fe^2+^ ions. The present results have important implications in expanding the scope of pathway complexity to produce a variety of products *via* kinetically controlled processes involving secondary metal ions and ligands.

## Abstract 2

The modification of metalloligands based on bis-type amideterpyridine platinum(ii) complexes not only induces the formation of helical supramolecular polymers but also introduces a kinetic trapping strategy in competitive conditions.

## Graphical abstract

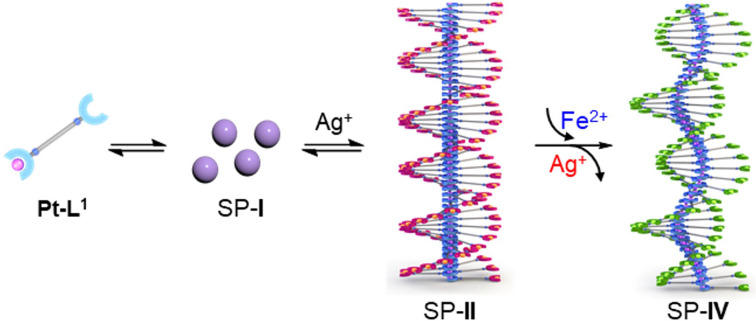

## Introduction

In both natural and artificial supramolecular systems, the formation of complicated multi-functional structures is often derived from concerted non-covalent interactions.^1–13^ The performance of specific functions in these systems requires particular molecular and supramolecular organization. Such specificity can be especially challenging due to the coexistence of multiple competitive pathways, known as pathway complexity.^14–16^ In the realm of biomolecules, such pathways frequently give rise to multifaceted systems characterized by an array of structures and functionalities.^17–19^ Therefore, the concept of pathway complexity in supramolecular polymers (hereafter ‘SPs’) has emerged as an invaluable paradigm for mimicking inherent natural processes. In other words, the strategic modulation of pathway complexity holds the potential to herald the development of functionally advanced SPs with enhanced architectural sophistication.^20,21^

Recently, several researchers have utilized the nature of pathway complexity to regulate the morphology and length of SPs.^22–27^ For example, some innovative attempts have been made in kinetically controlled supramolecular polymerization, emerging as pioneering strategies to yield self-assembled structures with properties surpassing those derived from equilibrium states.^28–34^ A promising alternative to attain kinetic control is the strategic design of monomers that are kinetically trapped with inactive conformations. Würthner and coworkers reported a perylene bisimide derivative where the monomeric state is stabilized by hydrogen bonding.^35^ They further demonstrated a novel strategy to control supramolecular polymerization *via* a chaperone-like two components system as a new kinetic trapping method, utilizing a squaraine dye.^36^ Similarly, Sánchez and colleagues reported the kinetically governed self-assembly behaviors of a suite of *N*-annulated perylenebisimides.^37^ They employed the trialkoxybenzamide group, linked to the *N*-annulated perylene fragment comprising a variable count of methylene units to optimize the formation of an H-bonded pseudocycle, thereby moderating the supramolecular polymerization. In contrast, Sugiyasu, Takeuchi and coworkers elucidated that porphyrin entities spatially align in a *J*-aggregate, typified by face-to-face stacking but with substantial offset, as a kinetic manifestation.^38,39^ Following an initial latency period, this *J*-aggregate form transitioned to the H-aggregate, characterized by face-to-face stacking but with augmented overlap. These kinetic controls have opened new pathways, catalyzing programmable self-assembly that generates specific aggregated species marked by regulated polydispersity, particularly evident in block copolymerization scenarios.^40^ To date, efforts to navigate the pathway complexity in purely organic π-conjugated systems have predominantly revolved around the integration of the amide functionality within the molecular blueprint, as well as the judicious alteration of temperature and solvent geometries to control aggregation modes.

Metallo-SPs incorporating d^8^ metal ions – namely, Pt(ii), Pd(ii) and Au(iii) – and interfaced with ligands like terpyridine, pyridine-triazole, and pyridine-benzimidazole, have attracted substantial interest.^41,42^ This intrigue arises from their inherent photophysical properties, notably luminescence, coupled with their propensity for metal–metal and π–π interactions.^43–46^ With specific regard to the Pt(ii) complex, Manners and colleagues pioneered their first attempt in 2015 to regulate the length of metallo-SPs based on tridentate pyridine-bis-tetrazole ligand.^47^ The Yam, Eisenberg, Tung, and Chen groups have probed alkynyl terpyridine-centric tridentate ligands in homo-metal complex systems, motivated by their captivating photophysical characteristics and their ensuing diverse applicative potential spanning molecular recognition, pH sensing, biomolecular labeling and beyond.^48–59^ Yam and coworkers, in particular, have offered pivotal insights, elucidating the nexus between self-assembly dynamics and structure–property relation in the supramolecular polymerization of alkynylplatinum(ii) terpyridine complexes.^60–63^

In particular, bis-type ligands not only dictate the selective formation of mononuclear complexes, so-called metalloligands, depending on the binding affinity for metal ions but also produce a variety of complexes with different stoichiometric ratios. Among these, some complexes may act as intermediates in supramolecular polymerization. Moreover, introducing a secondary ligand during supramolecular polymerization of the metalloligands might induce the formation of kinetic species due to competitive conditions which may suppress the spontaneous nucleation of monomers.

**Fig. 1:**
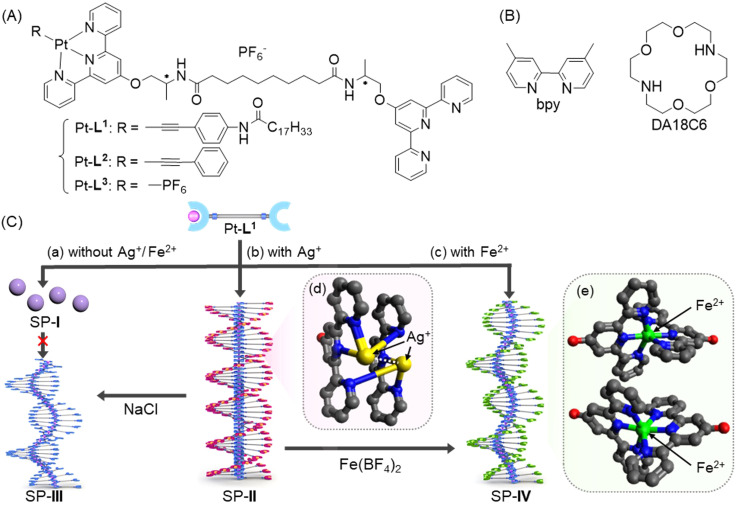
(A) Chemical structures of Pt-L^1^ and its reference compounds Pt-L^2^ and Pt-L^3^. (B) Chemical structures of the secondary ligands (bpy and DA18C6). (C) Proposed formation mechanism of SPs from Pt-L^1^ in the (a) absence and (b and c) presence of secondary metal ions (Ag^+^ and Fe^2+^); (a) SP-I formed by an isodesmic model and (b) SP-II (Pt-L^1^/Ag^+^ = 2 : 2) and (c) SP-IV (Pt-L^1^/Fe^2+^ = 2 : 1) with a left-handed helical structure formed by a cooperative model. (d and e) Partial chemical structures of the Pt-L^1^ complexes with (d) Ag^+^ and (e) Fe^2+^ ions obtained by DFT calculation.

Thus, we envisioned that the modification of metalloligands based on bis-type amideterpyridine may not only induce the formation of SPs but also might introduce a kinetic trapping strategy in competitive conditions. To probe this hypothesis, in this work, we employed monoalkynylplatinum(ii) diterpyridine complex (hereafter ‘Pt-L^1^’) with one terpyridine terminal remaining unoccupied (Fig. 1). In addition to the self-assembly of Pt-L^1^ alone, as depicted, the strategy involves the secondary metal ions (Ag^+^ and Fe^2+^) and ligands (bispyridine (hereafter ‘bpy’) and diaza-18-crown-6 (hereafter ‘DA18C6’)) to modulate the inherent pathway complexity associated with the Pt-L^1^ based supramolecular polymerization. In particular, we have emphasized the influence of secondary metal ions and ligands on the new kinetically controlled process because this is the first example of employing this strategy systemically in metallosupramolecular polymerization to control the pathway. The details of our approaches and the related investigations are discussed below.

## Results and discussion

### Synthesis of a monomeric building block (Pt-L^1^) and its reference compounds (Pt-L^2^ and Pt-L^3^)

As a versatile metalloligand, Pt-L^1^ was designed for supramolecular polymerization to be driven synergistically *via* a concerted combination of π–π stacking, intermolecular H-bond, and Pt⋯Pt interaction (Fig. 1). Incorporating an extended alkyl chain into the phenylacetylene framework is expected to promote fiber elongation, enhancing the flexibility of SPs. The unoccupied terpyridine site can be occupied by the secondary metal ion (*e.g.* Ag^+^), facilitating a metal–metal interaction, which may yield a pronounced helical structure.

The synthetic routes for the bis-type ligand and target metalloligands are shown in Scheme S1.† The chiral precursor *R*-L^2^ incorporating an *R*-alanine moiety was prepared using a known procedure.^64^ The bis-type ligand *R*-L^1^ was synthesized by the reaction of *R*-L^2^ with decanedioyl dichloride in the presence of triethylamine in dichloromethane. The precursor Pt-L^3^ (also as a reference compound) was prepared *via* the reaction of *R*-L^1^ with dichloro(1,5-cyclooctadiene)platinum(ii) to yield monochloroplatium(ii) complex PtCl-L^2^, followed by the anion exchange with NH_4_PF_6_. The main target metalloligand Pt-L^1^ was synthesized through the coupling of Pt-L^3^ with *N*-(4-ethylphenyl)stearamide (R-1, Scheme S2†) in the presence of CuI and diisopropylamine in dichloromethane. Another reference compound Pt-L^2^ was obtained by reacting phenylacetylene in an identical condition.

### Complexation behaviors of Pt-L^1^ with the secondary metal ions (Ag^+^ and Fe^2+^)

As a preliminary study, the complexation stoichiometries between Pt-L^1^ and secondary metal ions were investigated by high-resolution electrospray ionization mass spectrometry (HR-ESI-MS) in DMSO/H_2_O (9 : 1 v/v). In the presence of AgNO_3_ (1.5–3.0 equiv.), the intense peak corresponding to a 1 : 1 species ([(Pt-L^1^)Ag]^2+^, *m*/*z* 731.2876) was observed (Fig. S1–S5†). Some minor peaks corresponding to a 2 : 1 and a 3 : 2 species have vanished after 24 h ageing, reflecting the binding of one silver(i) to the free terpyridine moiety, regardless of the AgNO_3_ concentration. When Fe(BF_4_)_2_ (1.5 equiv.) was used as a secondary metal ion source, initially [(Pt-L^1^)_2_Fe]^4+^ (major, *m*/*z* 691.8191) and [(Pt-L^1^)Fe(H_2_O)]^3+^ (minor) coexists (Fig. S6†). After 5 h, the major peak intensity for the 2 : 1 species enhanced, indicating that both terpyridine sites are occupied by Fe^2+^, forming a distorted octahedral coordination geometry as the thermodynamic product.

The complexation behaviors were also monitored by UV-vis measurements. The spectrum of Pt-L^1^ displays prominent high-energy absorption bands spanning 290–370 nm and lower-energy bands in the 430–480 nm range (Fig. S7a†). These can be ascribed to the intraligand π–π* transition and the metal-to-ligand charge transfer (MLCT), respectively, with a superimposed ligand-to-ligand charge transfer (LLCT). In the presence of AgNO_3_ (1.5 equiv.), an alteration in the absorption tail in the 525–570 nm range is evident (Fig. S7a†), indicative of a metal-metal-to-ligand charge transfer (MMLCT) stemming from Pt⋯Pt interactions.^65^ Interestingly, in the presence of Fe(BF_4_)_2_ (1.5 equiv.) (Fig. S7b†), a pronounced shoulder band in the 520–580 nm range appears unlike that of AgNO_3_ probably due to the stronger MLCT interactions^66^ with Fe^2+^ over Ag^+^, similar to the results obtained from the HR-ESI-MS.

### Supramolecular polymerization of Pt-L^1^ without and with AgNO_3_

**Fig. 2:**
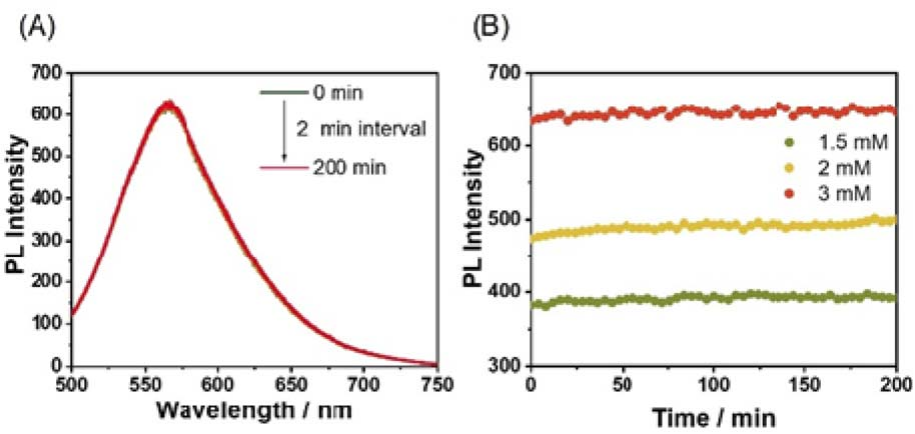
(A) Time-dependent PL spectra of Pt-L^1^ (3.0 mM, *λ*_ex_ = 420 nm) in DMSO/H_2_O (9 : 1 v/v) at 293 K. (B) Plots for times *vs.* PL intensity (565 nm) of different concentrations of Pt-L^1^ (1.5–3.0 mM).

In monitoring the supramolecular polymerization process including the mechanisms, time- and/or concentration-dependent measurements to follow the transition from dissolved monomeric species to the self-assembled aggregated state have been the mainstream. As shown in Fig. 2, the photoluminescence (PL) spectrum of Pt-L^1^ exhibits a strong emission band with a maximum at ∼565 nm dominated by the MLCT transition, indicating that the typical aggregation of the monomeric species occurs to form a supramolecular polymer (hereafter ‘SP-I’, see Fig. 1). In addition, the time-dependent spectra show no significant changes with time at 293 K, reflecting that SP-I is formed without Pt⋯Pt interactions, which we will discuss in the later part.^67^ We also observed temperature-dependent PL spectral changes of SP-I in DMSO and H_2_O (9 : 1 v/v). The PL band of SP-I observed in DMSO and H_2_O (9 : 1 v/v) at 298 K increased as the temperature rose (Fig. S8A†), which can be attributed to the π–π stacking interactions between the terpyridine moieties of Pt-L^1^ being disrupted as SP-I dissociates into monomeric species. This observation indicates that the PL band obtained at approximately 565 nm in the DMSO and H_2_O (9 : 1 v/v) mixture is associated with the MLCT of SP-I. Additionally, an intense PL band of Pt-L^1^ was observed at 560 nm in pure DMSO (Fig. S8B†). These observations suggest that the PL band detected at approximately 565 nm in the DMSO and H_2_O (9 : 1 v/v) mixture is associated with the MLCT of SP-I. The absorbance of SP-I in DMSO and H_2_O (9 : 1 v/v) around 280 nm is lower than that of monomeric Pt-L^1^ in pure DMSO (Fig. S8C†), due to the formation of SP-I with nanoparticles, driven by π–π stacking interactions.

**Fig. 3:**
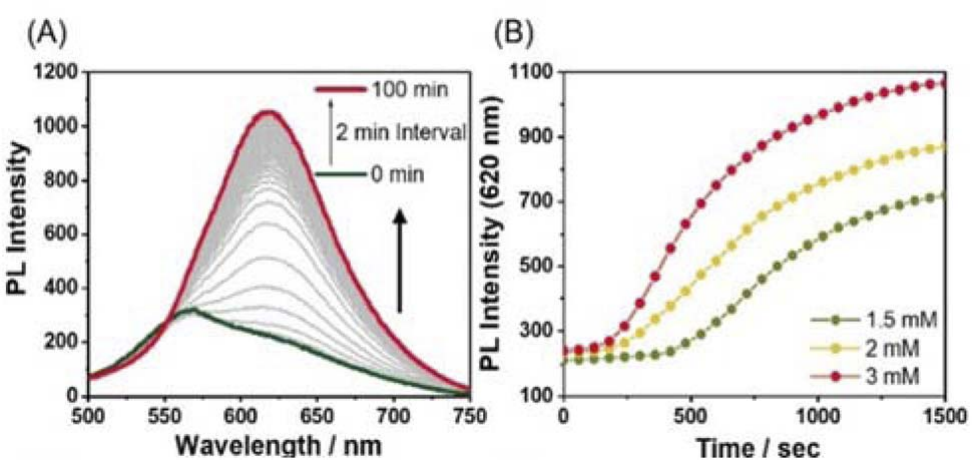
(A) Time-dependent PL spectra of Pt-L^1^ (2.0 mM, *λ*_ex_ = 420 nm) in the presence of AgNO_3_ (1.5 equiv.) in DMSO/H_2_O (9 : 1 v/v) at 293 K. (B) Plots for PL intensity at 620 nm *vs.* time at different concentrations of Pt-L^1^ (1.5–3.0 mM) in the presence of AgNO_3_ (1.5 equiv.).

The influences of the secondary metal ions on the formation of SPs have been investigated. After the addition of AgNO_3_ (1.5 equiv.) to Pt-L^1^ (1.0–3.0 mM), interestingly, a new emission band around 620 nm appears and gradually increases with time (Fig. 3). The red-shift (from ∼560 to ∼620 nm, Δ*λ* = ∼60 nm) with the intensity increase is mainly attributed to the Pt⋯Pt interactions characterized by MMLCT.^68^ The PL profile with a plot of the intensity *vs.* time shows typical sigmoidal curves with a well-recognized lag phase. This result seems to be associated with the transient existence of [(Pt-L^1^)_3_Ag_2_]^5+^ and [(Pt-L^1^)_2_Ag]^3+^, as evidenced by HR-ESI-MS data (Fig. S1†). Taken collectively, the above UV-vis, mass, and PL results for the complexations and supramolecular polymerizations suggest that SP-II generated in the presence of Ag^+^ seems to be derived from the aggregation of [(Pt-L^1^)_2_Ag_2_]^4+^ featuring the Pt⋯Pt interactions. Moreover, as the concentrations of Pt-L^1^ increase in the 1.5–3.0 mM range, a decrease in the duration of the lag phase is noted from 8 to 2 min (Fig. 3A and B), which could be related to the increased ratio of monomeric [(Pt-L^1^)_2_Ag_2_]^4+^ (Fig. S5†). These observations suggest a buffered release and self-nucleation of the monomer from SP-II, followed by autocatalytic growth of the remaining monomers *via* an on-pathway mechanism. When AgBF_4_ and AgPF_6_ were also employed in the same procedures, the lag times increased in the order of NO_3_^−^ < PF_6_^−^ < BF_4_^−^ (Fig. S9†), implying a kinetic tunability of the supramolecular polymerization depending on the anionic environment. We recorded the CD spectra of SP-I and SP-II in a DMSO/H_2_O mixture (9 : 1 v/v) (Fig. S10†). A very weak positive CD signal for SP-I was detected around 325 nm, while a relatively stronger positive CD signal for SP-II was observed at approximately 335 nm in the presence of AgNO_3_ (1.5 equiv.). These results suggest that SP-II forms a helical molecular arrangement, leading to the induction of chiral supramolecular polymers.

**Fig. 4:**
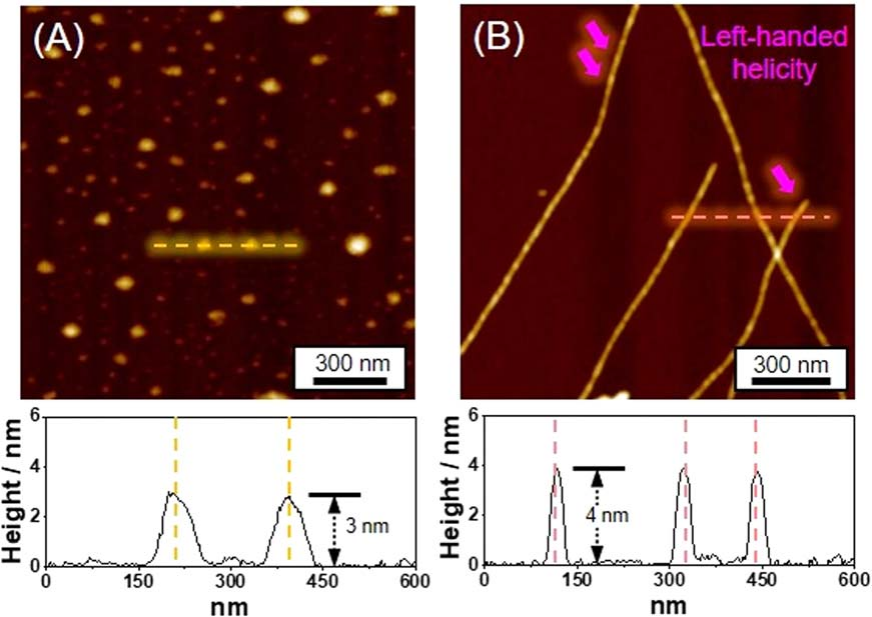
AFM images and height profiles of (A) SP-I (assembled from Pt-L^1^ (3.0 mM) only), and (B) SP-II (assembled from Pt-L^1^ (2.0 mM) + AgNO_3_ (1.5 equiv.)) in DMSO and H_2_O (9 : 1 v/v).

Distinct morphologies of the SPs were elucidated using atomic force microscopy (AFM). The SP-I assembled from Pt-L^1^ alone features nanoparticles with a height of 3 nm (Fig. 4A). The SP-I with nanoparticles is formed from the monomeric Pt-L^1^ in the DMSO and H_2_O (9 : 1 v/v) mixture, where one portion of the terpyridine is coordinated with Pt(ii) ion while another remains a free ligand. This asymmetric structure may contribute to the micellar property due to the higher polarity of the Pt(ii)-coordinated portion compared to the free terpyridine portion, which shows a hydrophobic effect in the aqueous environment. Whereas SP-II assembled from Pt-L^1^ with AgNO_3_ (1.5 equiv.) shows a left-handed fiber structure with a height of 4 nm on the HOPG surface (Fig. 4B and S11†), probably due to the Ag⋯Ag interactions within a tetrahedral structure, as demonstrated by DFT calculations (Fig. 1C(d)).

### Removal of Ag^+^ from SP-II: two different SPs with the same component

**Scheme 1:**
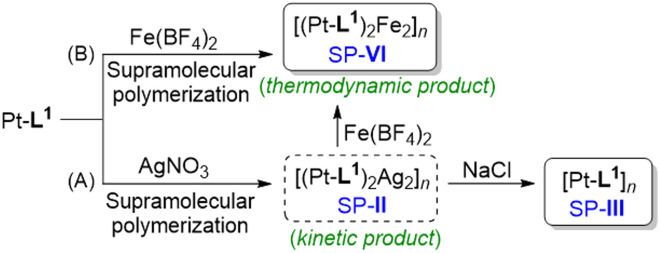
Proposed formation mechanism of SP-II, SP-III and SP-VI in the presence of secondary metal ions: (A) AgNO_3_ and (B) Fe(BF_4_)_2_, upon the addition of NaCl or Fe(BF_4_)_2_ to SP-II.

Having obtained SP-I and SP-II of types [Pt-L^1^]_*n*_ and [(Pt-L^1^)_2_Ag_2_]_*n*_^4+^ respectively, by direct methods, we propose an indirect formation of a new SP of type [Pt-L^1^]_*n*_ by removal of Ag^+^ from SP-II. Indeed, it is challenging to compare the new SP (hereafter ‘SP-III’, see Scheme 1) with SP-I because both products originate from different routes but contain the same component. For this, an excess amount of Cl^−^ (as NaCl) was added to SP-II solution to remove the bounded Ag^+^ (Scheme 1A). Upon addition, interestingly, the PL intensity at 620 nm increased by approximately 2-fold (Fig. S12†). This result is associated with the enhanced Pt⋯Pt interactions upon removal of Ag^+^. The distinct changes in the PL properties represent the appearance of a novel pathway. Moreover, SP-III exhibited a loosely packed left-handed fiber structure with a height of 6 nm (Fig. S13†) compared to SP-II. Furthermore, to demonstrate the presence of Ag^+^ ions in SP-II and SP-III before and after treatment with NaCl, the helical fibers were analyzed using scanning electron microscopy (SEM) combined with energy-dispersive spectrometry (EDS) microanalysis (Fig. S14†). The fibers of SP-II, prior to NaCl treatment, prominently displayed a characteristic peak associated with silver (Fig. S14B†). In contrast, the silver peak disappeared in the fiber of SP-III obtained after treatment with NaCl (6.0 equiv.) (Fig. S14D†). These findings suggest that SP-III consists solely of Pt-L^1^ as a building block, without the presence of an Ag^+^ complex. Considering the combined results, the supramolecular polymerization of Pt-L^1^ initially afforded SP-I as a kinetically controlled product. Meanwhile, SP-III obtained from SP-II*via* the removal of bounded Ag^+^ is thermodynamically stable (Scheme 1A). Consequently, the proposed give-and-take-away approach of the secondary metal ion is versatile for the controlled preparation of the kinetic and thermodynamic products.

### Supramolecular polymerization of Pt-L^1^ in the presence of Fe(BF_4_)_2_

**Fig. 5:**
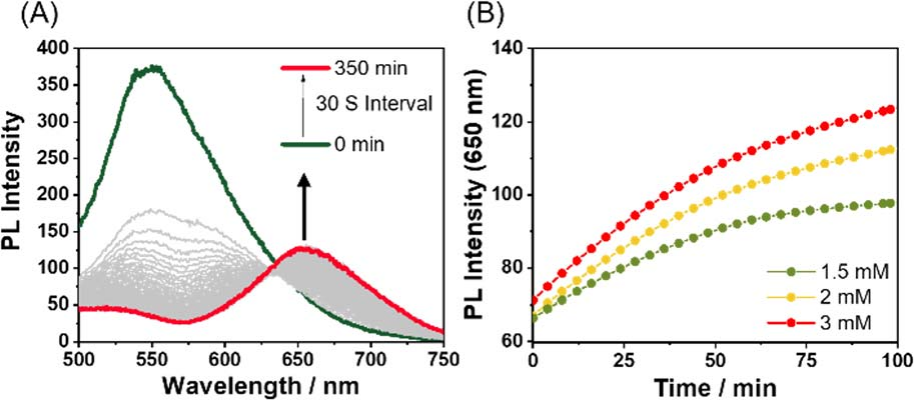
(A) Time-dependent PL spectra of Pt-L^1^ (2.0 mM, *λ*_ex_ = 420 nm) in the presence of Fe(BF_4_)_2_ (1.5 equiv.) in DMSO/H_2_O (9 : 1 v/v) at 293 K. (B) Plots for PL intensity at 650 nm *vs.* time at different concentrations of Pt-L^1^ (1.5–3.0 mM) in the presence of Fe(BF_4_)_2_ (1.5 equiv.).

When Fe^2+^ was used as a secondary metal ion by adding Fe(BF_4_)_2_ (1.5 equiv., Scheme 1B) to Pt-L^1^, the time-dependent PL spectra showed a larger red-shift (from ∼560 nm to ∼650 nm, Δ*λ* = ∼90 nm) than that of Ag^+^ and stabilized within approximately 100 min, with the intensity decrease (Fig. 5A, hereafter ‘SP-IV’, see Fig. 1C). This pronounced red shift to 650 nm is ascribed to the formation of excimers within the [(Pt-L^1^)_2_Fe]^4+^ complex during the polymerization process.^69^ Notably, the PL profiles displayed no lag phase (Fig. 5B), despite the coexistence of [(Pt-L^1^)Fe]^3+^ and [(Pt-L^1^)_2_Fe]^4+^ with 1 : 1 and 2 : 1 stoichiometric ratios, respectively. According to our UV-vis titrations to determine the stability constants between Pt-L^1^ and Fe^2+^, the above result could be explained by the higher stability of the 2 : 1 complex (log *K* = 11.87) than the 1 : 1 complex (log *K* = 5.57) (Fig. S15 and S16†). The stability constant of [(Pt-L^1^)_2_Fe]^4+^ is approximately 10^6^ times higher than that of the 1 : 1 complex, [(Pt-L^1^)Fe]^3+^. Consequently, the 1 : 1 complex [(Pt-L^1^)Fe]^3+^ rapidly converts to the 2 : 1 complex. Thus, no lag phase was observed in the presence of Fe^2+^ ions during the supramolecular polymerization because the 1 : 1 complex did not act as a kinetically trapped species in this process. SP-IV exhibits a left-handed fiber structure with a height of 5 nm (Fig. S17†). Consequently, the excimer [(Pt-L^1^)_2_Fe]^4+^ is an active precursor in forming SP-IV. The positive CD signal of SP-IV was also observed (Fig. S10c†), similar to that of SP-II, although its intensity was slightly lower compared to SP-II.

### Supramolecular polymerization of Pt-L^1^ in the presence of Ag^+^ and Fe^2+^*via* on-pathway process

**Fig. 6:**
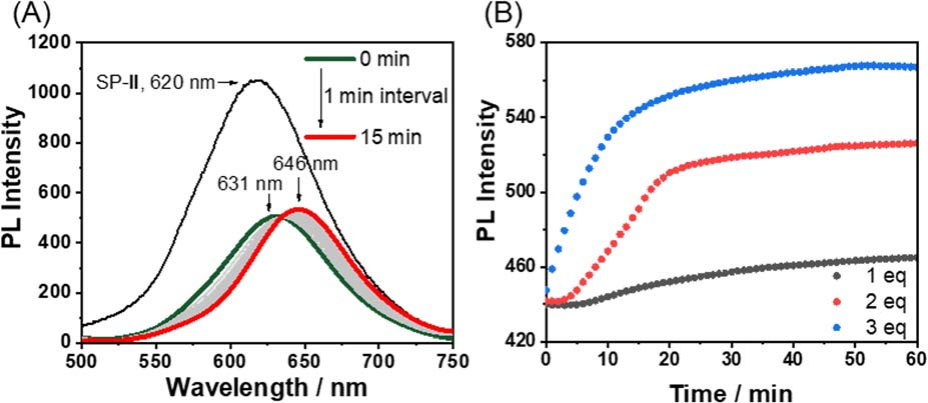
(A) Time-dependent PL spectra of SP-II (assembled from Pt-L^1^ + 1.5 equiv. of AgNO_3_, *λ*_ex_ = 420 nm) upon addition of Fe(BF_4_)_2_ (3.0 equiv.) in DMSO/H_2_O (9 : 1 v/v) at 293 K, *λ*_ex_ = 420 nm. (B) Plots of PL intensity (at 650 nm) *vs.* times at different concentrations of Fe(BF_4_)_2_.

Having obtained the results with Ag^+^ and Fe^2+^ separately, a competitive metal system was performed. In this approach, SP-II of type [(Pt-L^1^)_2_Ag_2_]_*n*_^4+^ was prepared and then exposed to Fe^2+^ to induce the consecutive reaction in the competition condition (Scheme 1B). Practically, the time-dependent PL spectral evolution of SP-II was recorded in the presence of Fe(BF_4_)_2_ in DMSO/H_2_O (9 : 1 v/v) (Fig. 6A and B). The added Fe^2+^ induced a notable red-shift from 620 nm to 630 nm and subsequently to 650 nm with an isosbestic point at 638 nm. The appearance of a new PL band near 631 nm is attributed to the metastable [(Pt-L^1^)Fe]^3+^ complex *via* the replacement of Ag^+^ with Fe^2+^. The higher stability of the complex of Pt-L^1^ with Fe^2+^ (log *K* = 11.87 for a 2 : 1 complex) than with Ag^+^ (log *K* = 7.70 for a 1 : 1 complex) supports the above spectroscopic results (Fig. S16†). In other words, the proposed competition condition allows the conversion of SP-II to SP-IV*via* the metastable intermediate [(Pt-L^1^)Fe]^3+^. Also, the duration of the lag phase from the sigmoidal PL profiles reduces with the increase of Fe^2+^ concentrations (Fig. 6B), indicating that the formation of SP-IV proceeds *via* an on-pathway mechanism retaining the left-handed helical shape (Fig. S18†). As a result, SP-IV can be generated in two routes; one is a direct method (*via* Pt-L^1^ + Fe^2+^) and another is a two-step method including competition condition (*via* SP-II + Fe^2+^) (Scheme 1). Consequently, in a scenario where both Ag^+^ and Fe^2+^ coexisted, the resulting SP-IV was identified to be a thermodynamic product (Scheme 1), whereas SP-II existed as the kinetic product in the presence of Fe^2+^. The left-handed helical fiber structure of SP-IV (Fig. S18†) is attributed to the formation of an octahedral coordination complex in the presence of Fe^2+^. The related thermodynamic studies are discussed in the later part. These results clearly show the decisive roles of the secondary metal ions in the pathway complexity of supramolecular polymerization depending on their relative reactivity.

### Comparative studies with the reference compounds (Pt-L^2^ and Pt-L^3^)

To support the supramolecular polymerization behaviors of Pt-L^1^ observed in the absence and presence of secondary metal ions, similar experiments were repeated using reference compounds Pt-L^2^ and Pt-L^3^, in which phenylacetylene and PF_6_ (as an R group) are bound to the Pt(ii) center, respectively (Fig. 1). In the time-dependent PL spectra of Pt-L^2^ and Pt-L^3^, each prominent MLCT band centered at ∼560 nm shows no significant changes with time (Fig. S19†). In the presence of AgNO_3_ or Fe(BF_4_)_2_, it is also noted that no appreciable spectral changes were observed (Fig. S20†), despite the ESI-MS results showing the formation of complexes of type [(Pt-L^2^)Ag]^2+^, [(Pt-L^3^)Ag]^2+^, [(Pt-L^2^)_2_Fe]^4+^ or [(Pt-L^3^)_2_Fe]^4+^ (Fig. S21–S24†), similar to the case of Pt-L^1^. These findings demonstrate that the flexibility of the extended alkyl chain in Pt-L^1^ plays a pivotal role in promoting the formation of Pt⋯Pt interaction during supramolecular polymerization. The AFM images for the supramolecular aggregates of Pt-L^2^ in both the absence and the presence of AgNO_3_ or Fe(BF_4_)_2_ show a sheet structure with a thickness of 3 nm (Fig. S25†). Similarly, SPs of Pt-L^3^ without a metal ion showed a sheet with a height of approximately 3 nm (Fig. S26†). These results suggest that the Pt⋯Pt and Ag⋯Ag interactions in Pt-L^1^ play an essential role in forming the helical structure during supramolecular polymerization.

### IR, Raman, and WXRD studies

To understand the reasons underlying the substantial enhancement of PL signal of the self-assembled Pt-L^1^ with metal ions, we employed various rcharacterization techniques such as FT-IR spectroscopy, Raman spectroscopy, and wide-angle X-ray diffraction (WXRD). In the FT-IR study, SP-I showed typical vibration bands of amide I and II at 1681 (C=O stretch) and 1578 cm^−1^ (Fig. S27b†), respectively. For SP-II, the amide I and II bands shifted to lower wavenumbers of 1645 (C=O stretch) and 1554 cm^−1^, respectively (Fig. S27c†), probably due to the enhanced intermolecular H-bonds.^70,71^ In addition, the vibration bands of SP-IV appeared at 1651 and 1550 cm^−1^ for amide I and II, respectively (Fig. S27d†). Additionally, the two NH stretching bands observed in the FT-IR spectra could be ascribed to the presence of two different types of amide bonds within the structures (Fig. S27a†). These results indicate that the intermolecular H-bonds observed in SP-II or SP-IV with a helical fiber structure are stronger than those observed in SP-I. The wide-angle X-ray diffraction (WXRD) patterns of SP-I show one small peak at *d* = 3.20, corresponding to π–π stacking (Fig. S28†). While, SP-II shows three peaks at *d* = 3.20, 2.82 and 2.79, which corresponds to π–π stacking, Pt–Pt, and Ag–Ag interactions, respectively. The Ag–Ag interaction in SP-II was observed at 120 cm^−1^ by the Raman spectroscopy (Fig. S29†).

### Thermodynamic studies for the formation of SPs

**Fig. 7:**
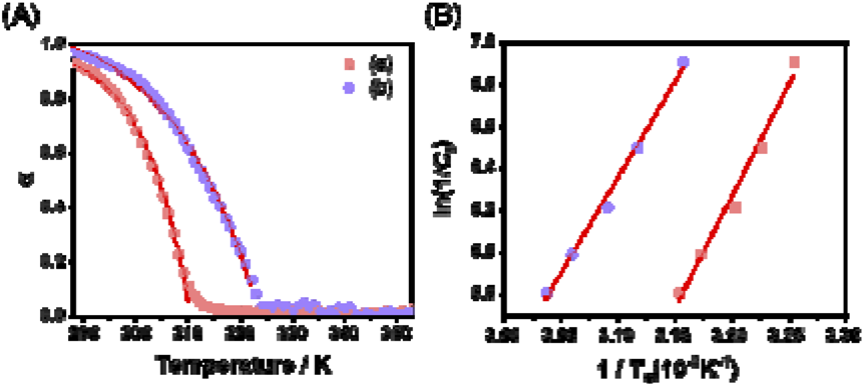
(A) Degree of aggregation (*α*) in different concentrations of Pt-L^1^ (2.0 mM) with 1.5 equiv. of (a) AgNO_3_ and (b) Fe(BF_4_)_2_ as a function of temperature at 620 and 650 nm in DMSO/H_2_O (9 : 1 v/v). (B) Natural logarithm of the reciprocal *C*_T_ as a function of the reciprocal *T*_e_ showing the linear relationship.

In the presence of AgNO_3_ and Fe(BF_4_)_2_, as can be seen in the above time-dependent PL and other studies, Pt-L^1^ exhibited different supramolecular polymerization behaviors compared to the conditions without metal ions. To support or reconfirm these behaviors, temperature-dependent PL spectral changes were also studied. The heating profile of SP-I (1.0–3.0 mM) in the absence of metal ions displayed a sigmoidal trend (Fig. S30A and S31†), reflecting an immediate disassembly into monomers and the supramolecular polymerization proceeds *via* an isodesmic model. Conversely, in the presence of AgNO_3_ and Fe(BF_4_)_2_, a marked reduction in the intensity at 620 or 650 nm was observed at 310 K and 320 K, respectively (Fig. S30B and C†). At the same time, there was an elevation in the intensity of the MLCT band centered around 560 nm (Fig. S30B and C†). Upon heating the solutions containing SP-II and SP-IV, the melting curves manifested a non-sigmoidal pattern (Fig. 7A and S32†). Taken collectively, these results suggest that both SP-II and SP-IV are formed *via* a cooperative model governed by a nucleation–elongation mechanism.

Upon determining the elongation binding constants (*K*_e_) and/or elongation temperature (*T*_e_) *via* curve fitting with cooperative model,^72^ the thermodynamic parameters (Δ*G*_e_, Δ*H*_e_ and Δ*S*) were calculated using the van't Hoff plot (Fig. 7 and S31C†). For example, the negative value of Δ*G*_e_ for SP-III (−19.1 kJ mol^−1^) is larger than that of SP-I (−17.8 kJ mol^−1^) (Table 1), indicating that the SP-III with a left-handed helical structure is the thermodynamically favored product, while SP-I with a spherical structure is a kinetically trapped product. On the other hand, the elongation temperatures for SP-IV, when formed in the presence of Fe^2+^, were roughly 9–12 K higher than those recorded for the Ag^+^ system (Fig. 7B). In particular, the Δ*G*_e_ value (−21.5 kJ mol^−1^) surpasses that obtained in the presence of Ag^+^ (−19.6 kJ mol^−1^), suggesting that SP-IV is thermodynamically more stable than SP-II. Consequently, it is possible that the SP-II formed with Ag^+^ can be spontaneously converted to SP-IV upon the introduction of Fe^2+^, following an on-pathway mechanism as shown in Scheme 1.

**Table 1:** Thermodynamic parameters for the formation of SPs in DMSO/H_2_O (9 : 1 v/v)

	Δ*H*_e_ (kJ mol^−1^)	Δ*S* (J K^−1^ mol^−1^)	Δ*G*_e_ (kJ mol^−1^)
SP-I	−165.0	−494.2	−17.8
SP-II	−88.7	−231.6	−19.6
SP-IV	−75.8	−182.0	−21.5

### Pathway complexity of SPs

**Fig. 8:**
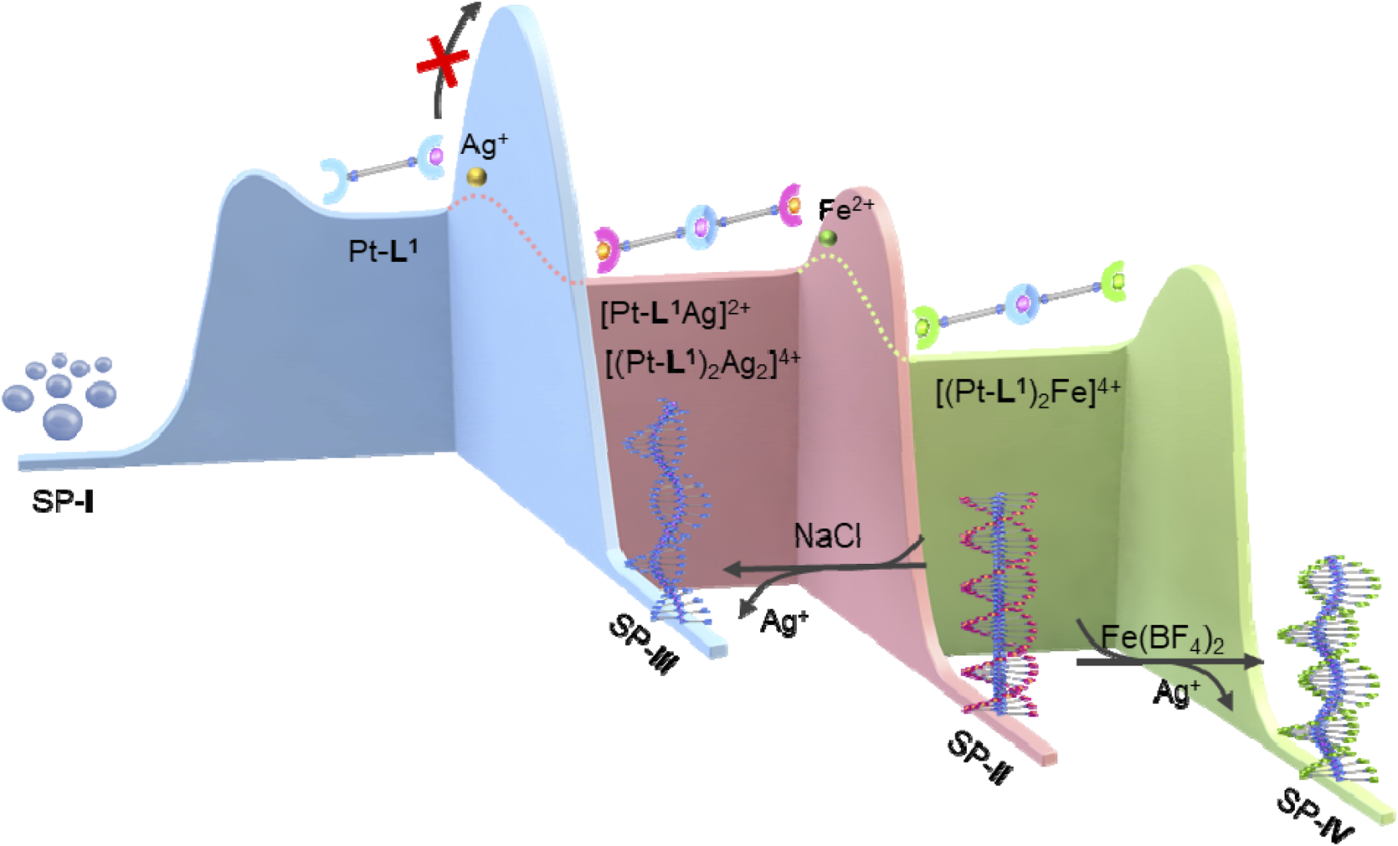
Proposed energy landscapes for the formation of SP-I, SP-II, SP-III and SP-IV: Pt-L^1^ (blue) only, with AgNO_3_ (pink), with Fe(BF_4_)_2_ (green).

Based on the above observations, we proposed an energy landscape representing the complexity of pathways and mechanism for the supramolecular polymerizations (Fig. 8). In the absence of metal ions, both the heating and cooling curves of SP-I exhibited a well-defined sigmoidal shape without hysteresis (Fig. S33†), indicating the supramolecular polymerization of Pt-L^1^ to SP-I*via* an isodesmic model. In the transformation to SP-II or SP-IV, with increasing concentrations of Pt-L^1^ in the presence of AgNO_3_ or Fe(BF_4_)_2_, sigmoidal kinetic profiles were observed, and the duration of the lag phase decreased (Fig. 3B). Furthermore, an increase in the concentration of Pt-L^1^ in the presence of Fe(BF_4_)_2_ rapidly led to reaching an equilibrium state (Fig. 5B). These observations indicate a buffered release and self-nucleation of the monomeric [(Pt-L^1^)_2_Ag_2_]^4+^ and [(Pt-L^1^)_2_Fe]^4+^ under pre-equilibrium between kinetic products, followed by autocatalytic growth of the remaining monomers *via* an on-pathway mechanism (Fig. 8). In particular, an increase in the concentration of Fe(BF_4_)_2_ in the transformation of SP-II to SP-IV led to a decrease in the lag time (Fig. 6B), indicating that increasing concentration of Fe(BF_4_)_2_ accelerates the conversion of SP-II to SP-IV. Therefore, SP-II acts as a kinetically trapped state in the presence of Fe(BF_4_)_2_.

Interestingly, in contrast to the SP-I, the hidden pathway of SP-III in Pt-L^1^ was obtained by adding NaCl to SP-II under kinetically controlled supramolecular polymerization. This is evidenced by the observation that heating and then cooling SP-III resulted in the formation of SP-I, and the obtained SP-III showed a non-sigmodal heating curve with *T*_e_ (315 K), indicating cooperative growth (Fig. S34†). Moreover, the SP-I did not transform into SP-III within the experimental timescale. This outcome suggests that the formation of SP-I and SP-III from Pt-L^1^ occurred through distinct pathways and mechanisms. Therefore, these results indicate that the pathway of Pt-L^1^ into SP-III was prevented due to a higher activation barrier than the pathway for SP-I. A rational strategy employing the complexation behavior of Pt-L^1^ with secondary metal ions revealed a hidden pathway to SP-III (Fig. 8).

### Kinetic control of supramolecular polymerization by secondary ligands

**Fig. 9:**
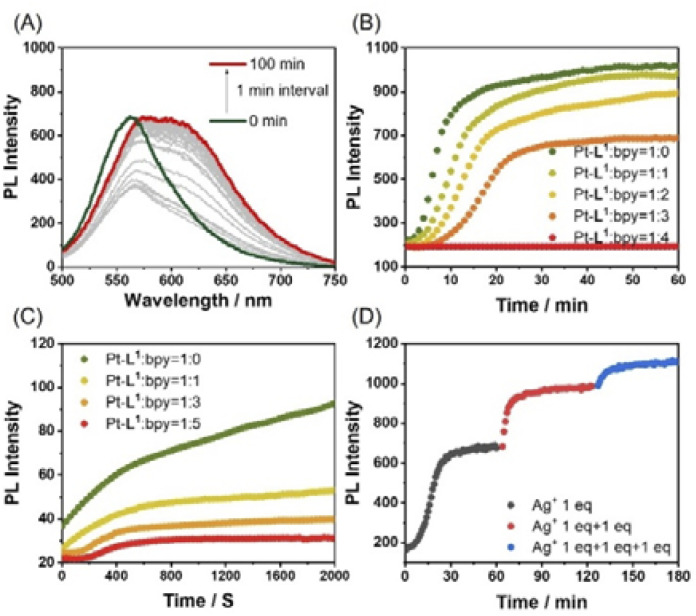
(A) Time-dependent PL spectra of a mixture of Pt-L^1^ (2.0 mM), bpy (6 mM), and AgNO_3_ (1.5 equiv.) in DMSO/H_2_O (9 : 1 v/v) at 293 K, *λ*_ex_ = 420 nm. (B and C) Plots of the PL intensity at 620 or 650 nm *vs.* times for supramolecular polymerization in the mixed (B) Pt-L^1^ and bpy with AgNO_3_ (1.5 equiv.), and (C) the mixed Pt-L^1^ and bpy with Fe(BF_4_)_2_ (1.5 equiv.) in DMSO/H_2_O (9 : 1 v/v). (D) Plot showing the PL intensity (at 620 nm) *vs.* time of Pt-L^1^ (2 mM) with stepwise addition of AgNO_3_ (1.0–3.0 equiv.).

**Scheme 2:**
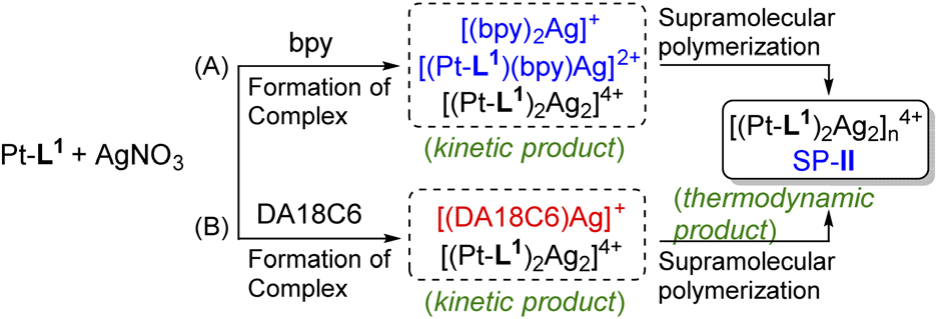
Proposed formation mechanism of SP-II in the presence of either (A) bpy and Ag^+^ or (B) DA18C6 and Ag^+^ as secondary ligands.

In the complexations of the mixed ligand system, there is the possibility of forming a variety of complexes including kinetically controlled products. Motivated by this trend, we guess that metallosupramolecular polymerizations can also be kinetically regulated in the presence of a competing coligand. To elucidate the concept of kinetic control in such systems, we monitored the time-dependent PL spectra of Pt-L^1^ in the presence of secondary ligands (bpy or DA18C6) upon coordination with AgNO_3_ or Fe(BF_4_)_2_ (Fig. 9). When bpy was used, the time-dependent PL intensity at 620 nm showed sigmoidal progressions (Fig. 9B), reflecting the cooperative pathway formation. The PL peak intensity at 620 nm diminished in comparison to that observed in the absence of bpy. The lag time increases by 1.5 to 3.0-fold with increasing bpy concentrations attributed to the [(bpy)_2_Ag]^+^ complex formation (Scheme 2) as evidenced by HR-ESI-MS (Fig. S35†). Consequently, some complexed species including [(bpy)_2_Ag]^+^ (log *K* = 7.10, Fig. S36†) and [(Pt-L^1^)(bpy)Ag]^2+^ serve as kinetic species during the supramolecular polymerization to build up SP-II of type [(Pt-L^1^)_2_Ag_2_]_*n*_^4+^ as a thermodynamic product (Fig. S37†). For comparison, pyridine was also employed as a secondary ligand. However, no significant changes were observed in the lag time for the supramolecular polymerization of a mixture containing Pt-L^1^ and pyridine in the presence of AgNO_3_ or Fe(BF_4_)_2_ (Fig. S38†), compared to pure Pt-L^1^ with metal ions (Fig. 3 and 5). The result suggests that the pyridine complex has not served as an intermediate in controlling the kinetics, due to its weaker coordinating ability than bpy. In the parallel experiments with DA18C6 (Fig. S39†), a similar trend with bpy was observed and the elongated lag phases exceeded those observed with bpy, probably due to the formation of a 1 : 1 complex, [(DA18C6)Ag]^+^ (Scheme 2), as a kinetic complex with higher stability (log *K* = 7.22) than that of bpy (Fig. S40 and S41†). As a result, no secondary ligands were incorporated into the formation of SPs due to the absence of driving forces for the self-assembly. However, the secondary ligands competitively influence the kinetic control of supramolecular polymerization.

More interestingly, in the presence of bpy or DA18C6 and Fe^2+^, distinct lag phases (*t*_50_ = 2.5 min with bpy and 3.5 min with DA18C6) were observed in the formation of SP-IV of [(Pt-L^1^)_2_Fe]^4+^ (Fig. 9C). The duration of the lag phase was kinetically modulated due to the formation of [(bpy)_3_Fe]^2+^ or [(DA18C6)Fe]^2+^ as a kinetic complex (Fig. S42–S45†), influenced by the concentration of the second ligands. The PL spectral changes exhibited a sigmoidal progression, reflecting a cooperative polymerization pathway governed by a nucleation–elongation mechanism. The observed lag phase is attributed to the stable formation of [(bpy)_3_Fe]^2+^ (log *K* = 11.56) (Fig. S46†), which are kinetically trapped species. Additionally, upon the addition of bpy to the equilibrium states of SP-II and SP-IV, no significant changes in PL intensity were observed (Fig. S47†). This result implied that bpy contributed to the kinetic pathway in supramolecular polymerization. Again, it is concluded that secondary ligand complexes can significantly influence kinetics as effective intermediates in metallosupramolecular polymerization.

To realize the kinetic growth pathway of the cooperative supramolecular polymerization, the addition of AgNO_3_ was modified. For example, 3.0 equiv. of AgNO_3_ or Fe(BF_4_)_2_ was added in three divided steps with an equal amount (1.0 equiv. in each step) to the mixture of Pt-L^1^ and bpy. As shown in Fig. 9D and S48,† the first addition resulted in a sigmoidal pattern, while the second and third were non-sigmoidal, suggesting that the SP formed during the first batch served as a nucleation seed. As shown in Fig. 9D, notably the magnitude of aggregation is reduced during the second and third batches (red and blue curved lines) compared to the first one (black curved line). The observed difference in the aggregation degree (*α*) during subsequent additions may be attributed to the *in situ* formation of kinetic products with the secondary ligand across the batches.

## Conclusions

In summary, we have demonstrated a novel strategy for controlling the pathway for the supramolecular polymerization of a monoalkynylplatinum(ii) terpyridine complex (Pt-L^1^) by introducing secondary metal ions and ligands. By employing a kinetic trapping strategy through the formation of complexes with various coordination numbers in the presence of secondary metal ions, we were able to regulate spontaneous metallosupramolecular polymerization. The introduction of the secondary metal ion to the Pt-L^1^ solution produced kinetic complexes, which act as a key factor in the kinetic control. In particular, the addition of Ag^+^ induced a large red-shift due to the Pt⋯Pt and Ag⋯Ag interactions during the formation of supramolecular polymer. These Pt⋯Pt and/or Ag⋯Ag interactions during supramolecular polymerization also act as additional driving forces. Furthermore, the self-assembly process was precisely and kinetically controlled by introducing secondary ligands to create competition complexes. In the presence of secondary ligands, some kinetic complexes that form complexes with the secondary ligand act as efficient kinetic species. Therefore, it can be concluded that SP-I, as a kinetical product, is formed by an isodesmic model, whereas SP-II, SP-III and SP-IV are generated by a cooperative model *via* a nucleation–elongation mechanism. In particular, SP-II obtained in the presence of Ag^+^ ion transforms to SP-III or SP-IV through an on-pathway mechanism upon the introduction of NaCl or Fe(BF_4_)_2_. As a result, we have shown a novel methodology on how to control the pathway of metallosupramolecular polymerization precisely by employing secondary metal ions and/or ligands which form competitive complex species in the self-assembly process. Consequently, this study provides new insights into the molecular design that rationally governs the aggregation pathways of multi-component systems, with implications for the development of optical materials.

## Data availability

The data supporting this article have been included as part of the ESI.†

## Author contributions

M. K., H. C. and M. K. contributed to the design and performed the preparation and characterization of supramolecular polymers. S. K. and J. C. contributed ESI-MS observation. S. Y. and E. L. determined stability constants. J. H. J. and S. H. J. directed the project. All authors contributed to the discussion and the preparation of the paper.

## Conflicts of interest

There are no conflicts to declare.

## Supplementary Material

SC-OLF-D4SC06083K-s001
