# Supplementary material for: Pathway control in metallosupramolecular polymerization of a monoalkynylplatinum(ii) terpyridine complex through competitive complex formation
Source: Chem Sci. 2024 Nov 6;15(47):19729–38. doi: 10.1039/d4sc06083k (PMC11575569; doi:10.1039/d4sc06083k)
Supplement: SC-015-D4SC06083K-s001 [file SC-015-D4SC06083K-s001.pdf]

Supplementary Information (SI) for Chemical Science.
This journal is © The Royal Society of Chemistry 2024

Electronic Supplementary Information (ESI)

Pathway control in metallosupramolecular polymerization of monoalkynylplatinum(II) terpyridine complex through competitive complex formation

Minhye Kim,[a] Heekyoung Choi,[a] Minjoo Kim,[a] Seonghan Kim,[c] Seohyeon Yun,[d] Eunji Lee,[d] Jaeheung Cho,[c] Sung Ho Jung,*[a,b] and Jong Hwa Jung*[a,b]

[a] Department of Chemistry, Gyeongsang National University, Jinju 52828, Republic of Korea

[b] Research Institute of Advanced Chemistry, Gyeongsang National University, Jinju 52828, Republic of Korea

[c] Department of Chemistry, Ulsan National Institute of Science and Technology, Ulsan 44919, Republic of Korea

[d] Department of Chemistry and Advanced Materials, Gangneung-Wonju National University, Gangneung 25457, Republic of Korea

Table of Contents

1. Method

1.1 General Characterization ········ S3

1.2 Atomic Force Microscopy (AFM) Studies ········ S3

1.3 UV-Vis Studies ········ S3

1.4 Preparation of Silver(I) or Iron(II) Complexes ········ S3

1.5 Preparation of Silver(I) or Iron(II) Complexes with the Secondary Ligands······ S3

1.6 Thermodynamic Studies ········ S4

1.7 Calculation of Complex Stability Constants ········ S4

1.8 Theoretical Calculations ········ S4

2. Synthesis and Characterization

2.1 Synthesis of *R*-$\mathbf{L}^2$ ········ S5

2.2 Synthesis of *R*-$\mathbf{L}^1$ ········ S5

2.3 Synthesis of PtCl-$\mathbf{L}^2$ ········ S5

2.4 Synthesis of **R-1** ········ S6

2.5 Synthesis of Pt-$\mathbf{L}^3$ ········ S6

2.6 Synthesis of Pt-$\mathbf{L}^2$ ········ S6

2.7 Synthesis of Pt-$\mathbf{L}^1$ ········ S7

3. Supplementary Schemes and Figures

3.1 Scheme S1 and S2 ········ S8

3.2 Figures S1-S48 ········ S9

4. Analytical Data

4.1 ^{1}H- and ^{13}C-NMR Spectoscopy ········ S57

4.2 ESI-MS Spectrometry ········ S62

5. Supplementary References ········ S66

Supplementary Data

1. General

1.1 General Characterization

The ^{1}H and ^{13}C NMR spectra were taken on a Bruker DRX 300 and a Bruker DRX 500. The high-resolution mass spectra (HR-MS) were measured by electrospray ionization (ESI) with a micro TOF Focus spectrometer from SYNAPT G2 (Waters, U.K.). IR spectra were observed over the range 500-4000 cm^{-1}, with a Thermo Scientific Nicolet iS 10 model. Powder X-ray patterns (PXRD) were recorded on a Rigaku model NANOPIX X-ray diffractometer with a Cu K_{α} radiation source.

1.2 Atomic Force Microscopy (AFM) Studies

Atomic force microscope (AFM) imaging was performed by using XE-100 and a PPP-NCHR 10 M cantilever (Park systems). The AFM samples were prepared by spin-coating (3000 rpm) onto freshly cleaved Muscovite Mica, and images were recorded with the AFM operating in a noncontact mode in air at RT with the resolution of 1024 × 1024 pixels, using moderate scan rates (0.3 Hz) and analyzed using XEI software developed by Park systems.

1.3 PL Studies

The photoluminescence (PL) spectra were recorded on a JASCO FP-8650 fluorescence spectrophotometer. The PL spectra were measured over the range of 250-800 nm using a quartz cell with a 10 mm path length. Scans were taken at a rate of 400 nm/min with a sampling interval of 0.5 nm and a response time of 0.5 s. To investigate the supramolecular polymerization process, temperature- and time-dependent PL spectral changes were monitored with an excitation wavelength of 420 nm.

1.4 Preparation of Silver(I) and Iron(II) Complexes

Pt-$\mathbf{L}^{1}$ (1.5-3.0 mM) was dissolved by heating in DMSO. $AgNO_3$ or $Fe(BF_4)_2$ (1.5-3.0 equivalent) was dissolved in H_2O. The Pt-$\mathbf{L}^{1}$ solution was added to $AgNO_3$ or $Fe(BF_4)_2$ (1.5-3.0 equivalent) solution. The mixed solvent ratio was maintained at DMSO/H_2O (9:1 v/v).

1.5 Preparation of Silver(I) or Iron(II) Complexes with Added Secondary Ligands

Pt-$\mathbf{L}^{1}$ (2 mM) and bpy or DA18C6 were dissolved by heating in DMSO, respectively. $AgNO_3$ or $Fe(BF_4)_2$ (1.5 equivalent) was dissolved in H_2O. The Pt-$\mathbf{L}^{1}$ and secondary ligand solution was added to $AgNO_3$ or $Fe(BF_4)_2$ solution. The mixed solvent ratio was maintained at DMSO/H_2O (9:1 v/v).

1.6 Thermodynamic Studies

The molar fraction of aggregated molecules (α_{agg}) at a certain temperature was calculated from the intensity at 565 nm, 620 nm, or 650 nm. Here, intensity (α_{agg}) and intensity (mono) refer to the emission intensities of fully aggregated states (at the lowest temperature) and purely monomeric states (at the highest temperature), respectively, while intensity is the emission intensity at a given temperature (T).[1]

$$\alpha_{agg} = 1 - \frac{intensity(agg) - intensity(T)}{intensity(agg) - intensity(mono)}$$

The plot of α_{agg} versus temperature provides heating curves with non-sigmoidal (*cooperative mechanism*) and sigmoidal (*isodesmic mechanism*) shape, which were fitted using the models proposed by Meijer *et al.*[2] The $\Delta H°$, $\Delta S°$, and $\Delta G°$ values were obtained using the van't Hoff equation. The van't Hoff plots were produced as proposed in the literature.[3] The ΔS and ΔH values used in the cooperative supramolecular polymerization models were determined by fitting of the heating curves,[4-6] obtained from the temperature-dependent PL spectra.

1.7 Calculation of Complex Stability Constants

The UV-vis titrations of Pt-$\mathbf{L}^{1}$ (20 μM) with $AgNO_3$ or $Fe(BF_4)_2$ (0-3.0 equiv.) were performed in DMSO/H_2O (9:1 v/v) at 293 K. The titration data were fitted into a desired binding model with HyperSpec to calculate stability constants; 1:1 and 2:2 (ligand-to-metal) stoichiometries in the silver(I) complexations and 1:1 and 2:1 stoichiometries in the iron(II) complexations.[7-9]

The UV-vis titrations of bpy (200 μM) with $AgNO_3$ or $Fe(BF_4)_2$ (0-3.0 equiv.) were performed in DMSO/H_2O (9:1 v/v) at 293 K. The titration data were fitted into a desired binding model with HyperSpec to calculate stability constants; 2:1 (ligand-to-metal) stoichiometry for the silver(I) complexation and 3:1 stoichiometry for the iron(II) complexation.

The NMR titration of DA18C6 (5 mM) with $AgNO_3$ (0-2.0 equiv.) was performed in DMSO-d_6/D_2O (9:1 v/v) at 293 K. The titration data were fitted into a desired binding model with HyperNMR to calculate stability constants for 1:1 (ligand-to-metal) stoichiometry.

1.8 Theoretical Calculations

The density functional theory (DFT) calculations were performed to optimize the silver(I) complex systems using the Gaussian 09 package.[10] The unrestricted B3LYP functional was employed for all optimizations and frequency calculations with Def2-SVP level of theory for all atoms.[11-13] All calculations were performed in the gas phase. All the optimized structures were confirmed by vibrational frequency analysis with no imaginary frequency.

2. Synthesis and Characterization

2.1 Synthesis of *R*-$\mathbf{L}^2$

R -(−)-2-amino-1-propanol (0.28 g, 3.7 mmol) was added to a stirred suspension of powdered KOH (1.05 g, 18.7 mmol) in dry DMSO (20 mL) at 60 °C. After 30 min, 4′-chloro-2,2′:6′,2″-terpyridine (1.00 g, 3.7 mmol) was added to the mixture. The mixture was then stirred for 4 h at 70 °C and poured into 600 mL of distilled water thereafter. CH_2Cl_2 (3 × 200 mL) was used to extract the aqueous phase. Residual water in dichloromethane was dried over Na_2SO_4 and CH_2Cl_2 was removed in a vacuum, and the desired product was purified by recrystallization with ethyl acetate to give 0.72 g (72%) of *R*-$\mathbf{L}^2$. Mp = 118.3 °C; IR (KBr pellet): 3375, 2964, 2926, 2846, 1577, 1565, 1473, 1439, 1403, 1353, 1204, 799 cm^{-1}; ^{1}H NMR (300 MHz, $CDCl_3$): δ 8.70 (tdd, *J* = 4.8, 1.8, 0.9 Hz, 2H), 8.62 (dt, *J* = 8.0, 1.1 Hz, 2H), 8.02 (s, 2H), 7.84 (td, *J* = 7.7, 1.8 Hz, 2H), 7.33 (ddd, *J* = 7.4, 4.8, 1.2 Hz, 2H), 4.14 (dd, *J* = 9.0, 4.1 Hz, 1H), 3.94 (dd, *J* = 9.1, 7.6 Hz, 1H), 3.41 (dddd, *J* = 10.6, 7.6, 6.6, 4.2 Hz, 1H), 1.21 (d, *J* = 6.5 Hz, 3H); ^{13}C NMR (125 MHz, DMSO-d_6): δ 167.2, 157.1, 155.3, 149.7, 137.9, 125.0, 121.3, 107.3, 75.1, 46.2, 20.43; HR-ESI-Mass (m/z) calculated for $C_{18}H_{18}N_4O$ $[M]^+$: 306.3690, Found $[M]^+$: 306.3690.

2.2 Synthesis of *R*-$\mathbf{L}^1$

R-$\mathbf{L}^1$ was prepared according to a literature procedure.[14] In a two-neck flask, *R*-$\mathbf{L}^2$ (0.50 g, 1.64 mmol) and triethylamine (TEA) (0.1 mL, 0.72 mmol) were added to dry CH_2Cl_2 (10 mL). After cooling the solution in an ice bath, sebacoyl chloride (0.16 mL, 0.75 mmol) was added dropwise. The reactant was stirred for 3 h at room temperature. The crude product was recrystallized from CH_2Cl_2 to give a white crystalline solid *R*-$\mathbf{L}^1$ in 49.7% yield (0.632 g). Mp = 198 °C; IR (KBr pellet): 3428, 3311, 2929, 2845, 1640, 1582, 1563, 1466, 1446, 1407, 1362, 1207, 1038, 785 cm^{-1}; ^{1}H NMR (300 MHz, DMSO-d_6): δ 8.68 (m, 8H), 7.99 (m, 8H), 7.87 (d, *J* = 7.5 Hz, 2H), 7.50 (ddd, *J* = 7.7, 4.8, 1.6 Hz, 4H), 4.15 (m, 6H), 2.02 (t, *J* = 7.3 Hz, 4H), 1.42 (d, *J* = 7.5 Hz, 4H), 1.21 (s, 3H), 1.19 (s, 3H), 1.13 (s, 8H); ^{13}C NMR (125 MHz, DMSO-d_6): δ 172.3, 167.1, 157.2, 155.3, 149.7, 137.8, 125.0, 121.3, 107.2, 70.9, 44.1, 35.9, 29.2, 29.0, 25.7, 17.6; HR-ESI-Mass (m/z) calculated for $C_{46}H_{50}N_8O_4$ $[M]^+$: 778.3955, Found $[M]^+$: 778.3954.

2.3 Synthesis of PtCl-$\mathbf{L}^2$

In a two-neck flask, *R*-$\mathbf{L}^1$ (0.30 g, 0.385 mmol) in MeOH (10 mL) was refluxed at 80 °C. After R-$\mathbf{L}^1$ is completely dissolved, dichloro(1,5-cyclooctadiene)platinum(II) (0.14 g, 0.385 mmol) was added and then the solution was converted into yellow. The solvents were removed using a rotary evaporator. The crude product was purified via column chromatography (Al_2O_3 using dichloromethane/methanol, 95:5). Then, the organic solvent was removed using a rotary evaporator and dried under vacuum. Yield: 54.1% (0.21 g); IR (KBr pellet): 3466, 3042, 2931, 2853, 1661, 1608, 1563, 1482, 1366, 1221, 1051, 989, 799 cm^{-1}; ^{1}H NMR (300 MHz, DMSO-d_6) δ 8.73 – 8.61 (m, 4H), 8.61 – 8.47 (m, 4H), 8.42 (td, *J* = 7.9, 1.5 Hz, 2H), 8.20 (s, 2H), 8.13 (d, *J* = 6.4 Hz, 1H), 7.93 (td, *J* = 7.7, 1.8 Hz, 3H), 7.85 (d, *J* = 4.0 Hz, 4H), 7.45 (ddd, *J* = 7.5, 4.7, 1.2 Hz, 2H), 4.23 (dt, *J* = 19.4, 7.8 Hz, 4H), 4.10 (d, *J* = 5.8 Hz, 2H), 3.07 (s, 1H), 2.09

(dt, J = 11.4, 7.2 Hz, 4H), 1.47 (s, 2H), 1.27 – 1.17 (m, 15H).; HR-ESI-Mass (m/z) calculated for $C_{46}H_{50}ClN_8O_4Pt$ [M]$^+$: 1008.3286, Found [M]$^+$: 1008.3338.

2.4 Synthesis of R-1

In a 100 mL round-bottom flask, sodium hydroxide (0.07 g, 1.7 mmol, 1.0 equiv.) was added and 4-ethyleniline (0.18 mL, 1.7 mmol, 1.0 equiv.) was added. A hexane solution (30 mL), containing stearoyl chloride (0.50 g, 1.7 mmol, 1.0 equiv.) was slowly added and the reaction mixture was stirred for 5 h. After the reaction was completed, the water layer was filtered. The organic phase was removed under reduced pressure using the organic phase as a solvent. The crude product was washed with isopropanol to give **R-1** (0.50 g, 80%); IR (ATR): 3279, 2912, 2849, 1660, 1602, 1535, 1473, 1461, 1402, 1295, 852 cm^{-1}; ^{1}H NMR (300 MHz, Chloroform-d) δ 7.62 (s, 1H), 7.56 (d, J = 7.7 Hz, 1H), 7.28 (d, J = 7.7 Hz, 1H), 7.25 – 7.20 (m, 1H), 7.09 (s, 1H), 3.06 (s, 1H), 2.35 (t, J = 7.5 Hz, 2H), 1.78 – 1.67 (m, 2H), 1.25 (s, 30H), 0.92 – 0.83 (m, 3H); ^{13}C NMR (75 MHz, $CDCl_3$) δ 171.11, 137.66, 128.75, 127.60, 122.79, 122.54, 119.98, 82.86, 37.56, 31.66, 29.43, 29.39, 29.34, 29.20, 29.10, 28.98, 25.28, 22.43, 13.86; HR-ESI-Mass (m/z) calculated for $C_{26}H_{41}NO$ [M]$^+$: 383.3188, Found [M+H]$^+$: 384.4222.

2.4 Synthesis of Pt-**L^3**

In a two-neck flask, PtCl-**L^2** (0.10 g, 0.10 mmol) in MeOH (10 mL) was refluxed at 80 °C. After PtCl-**L^2** was completely dissolved, NH_4PF_6 (0.16 g, 0.10 mmol) was added and the solution was converted into yellow. The solvents were removed using a rotary evaporator. The product was purified by recrystallization with water and MeOH. Yield: 80% (0.08 g); IR (KBr pellet): 3370, 3242, 2925, 2850, 1646, 1604, 1551, 1484, 1350, 1212, 1074, 1002, 710 cm^{-1}; ^{1}H NMR (300 MHz, DMSO-d_6) δ 8.65 (td, J = 5.6, 1.5 Hz, 4H), 8.57 (d, J = 8.0 Hz, 2H), 8.52 (d, J = 8.0 Hz, 2H), 8.42 (td, J = 7.9, 1.5 Hz, 2H), 8.19 (s, 2H), 8.14 (d, J = 6.1 Hz, 1H), 7.93 (td, J = 7.7, 1.8 Hz, 3H), 7.88 – 7.79 (m, 4H), 7.45 (ddd, J = 7.6, 4.8, 1.2 Hz, 2H), 4.22 (dd, J = 14.4, 7.9 Hz, 4H), 4.10 (d, J = 5.8 Hz, 2H), 3.06 (td, J = 7.3, 4.8 Hz, 1H), 2.09 (dt, J = 13.5, 7.1 Hz, 4H), 1.47 (s, 2H), 1.28 – 1.17 (m, 15H).; HR-ESI-Mass (m/z) calculated for $C_{46}H_{50}F_6N_8O_4PPt$ [M]$^+$: 1118.3240, Found [M]$^+$: 1118.4215.

2.5 Synthesis of Pt-**L^2**

Pt-**L^3** (0.50 g, 0.50 mmol) and phenylacetylene (0.71 g, 6.94 mmol) were dissolved in distilled dichloromethane (20 mL). Diisopropylamine (0.5 mL) was added to the solution mixture and degassed by N_2 bubbling with sonication. A catalytic amount of CuI (20 mg) was added to the mixture and stirred in the dark for 10 h. Excess water was added to the reaction mixture. The product was collected by filtration and dried under vacuum to give Pt-**L^2** as dark brown solid in 60.5% yield (0.32 g); IR (KBr pellet): 3458, 3351, 3005, 1709, 1648, 1438, 1401, 1311, 1186, 1020, 953cm^{-1}; ^{1}H NMR (300 MHz, DMSO-d_6) δ 9.00 – 8.88 (m, 2H), 8.66 (ddd, J = 4.8, 1.8, 0.9 Hz, 2H), 8.62 – 8.48 (m, 4H), 8.41 (td, J = 7.9, 1.6 Hz, 2H), 8.23 (s, 2H), 8.07 (d, J = 6.3 Hz, 1H), 7.98 – 7.86 (m, 5H), 7.85 – 7.75 (m, 2H), 7.52 – 7.41 (m, 4H), 7.40 – 7.31 (m,

2H), 7.31 – 7.24 (m, 1H). 4.31 – 4.21 (m, 1H), 4.10 (q, J = 5.3 Hz, 2H), 3.17 (d, J = 5.3 Hz, 3H), 2.14 – 1.88 (m, 4H), 1.46 (s, 3H), 1.30 – 1.10 (m, 15H).; HR-ESI-Mass (m/z) calculated for $C_{54}H_{55}N_8O_4Pt$ $[M]^+$: 1074.3989, Found $[M+H]^+$: 1075.3357.

2.6 Synthesis of Pt-L[1]

In a 100 mL round-bottom flask Pt-**L**[3] (0.20 g, 0.20 mmol) and **R-1** (0.15g, 0.39 mmol) were dissolved in distilled DCM (20 mL). Diisopropylamine (0.5 mL) and CuI (10 mg) were added to the reaction mixture and stirred in the dark for 12 h. The reaction mixture was extracted with DCM and water. The organic phase was dried over sodium sulfate and solvents were filtered and removed under reduced pressure. The crude product was purified by Al_2O_3 column chromatography (MeOH/chloroform 3:100 v/v) to give Pt-**L**[1] (0.22 g, 81.8%); IR (ATR): 3451, 3294, 2922, 2854, 1681, 1644, 1578, 1554, 1463, 1400, 1366, 1034, 840, 790 cm^{-1}; ^1H NMR (500 MHz, DMSO-d_6) δ 8.69 (s, 4H), 8.50 (s, 4H), 8.33 (s, 2H), 7.95 (s, 5H), 7.62 (s, 2H), 7.57 (s, 2H), 7.42 (t, J = 7.7 Hz, 3H), 7.34 (d, J = 7.9 Hz, 1H), 4.30 (s, 3H), 2.55 (s, 3H), 2.33 (t, J = 7.2 Hz, 3H), 1.63 (d, J = 8.6 Hz, 3H), 1.50 (s, 5H), 1.28 (s, 43H), 1.13 (s, 2H), 0.88 (t, J = 6.7 Hz, 4H).; HR-ESI-Mass (m/z) calculated for $C_{72}H_{90}N_9O_5Pt$ $[M]^+$: 1355.6707, Found $[M]^+$: 1355.3509.

3. Supplementary Schemes and Figures

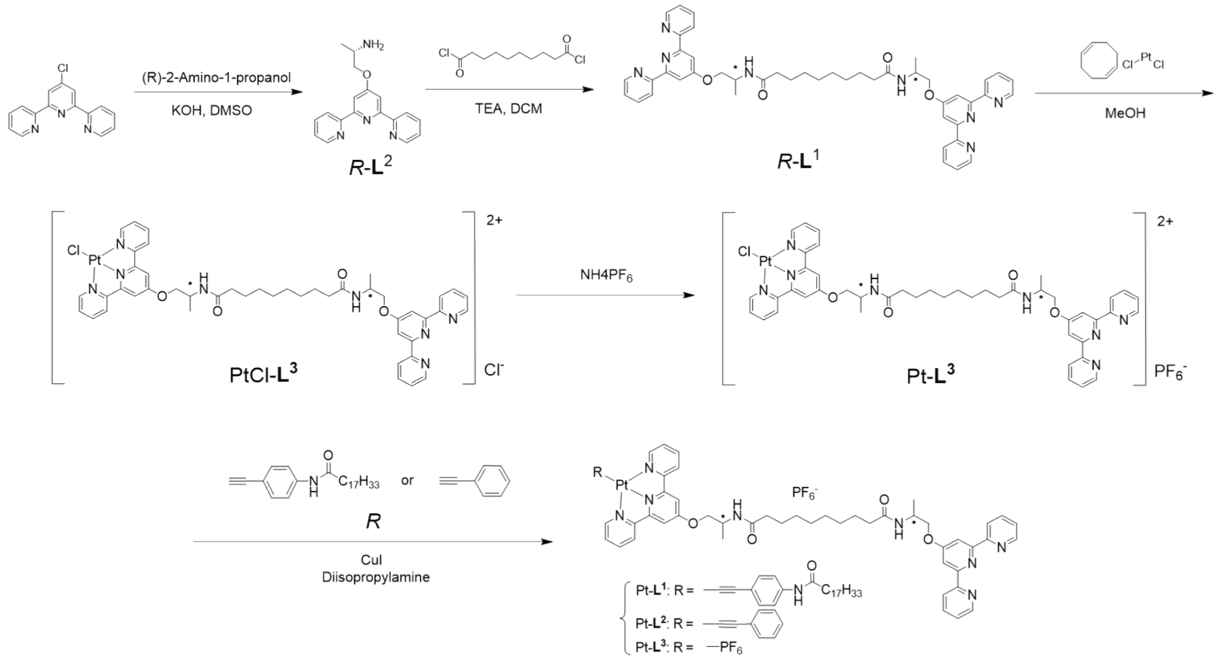

Scheme S1. Synthetic procedures for Pt-**L**[1], Pt-**L**[2], and Pt-**L**[3].

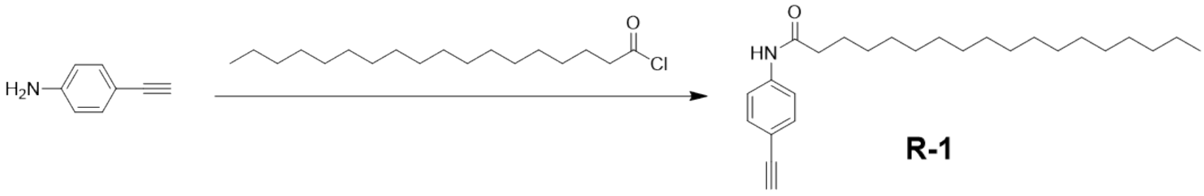

Scheme S2. Synthesis of **R-1**.

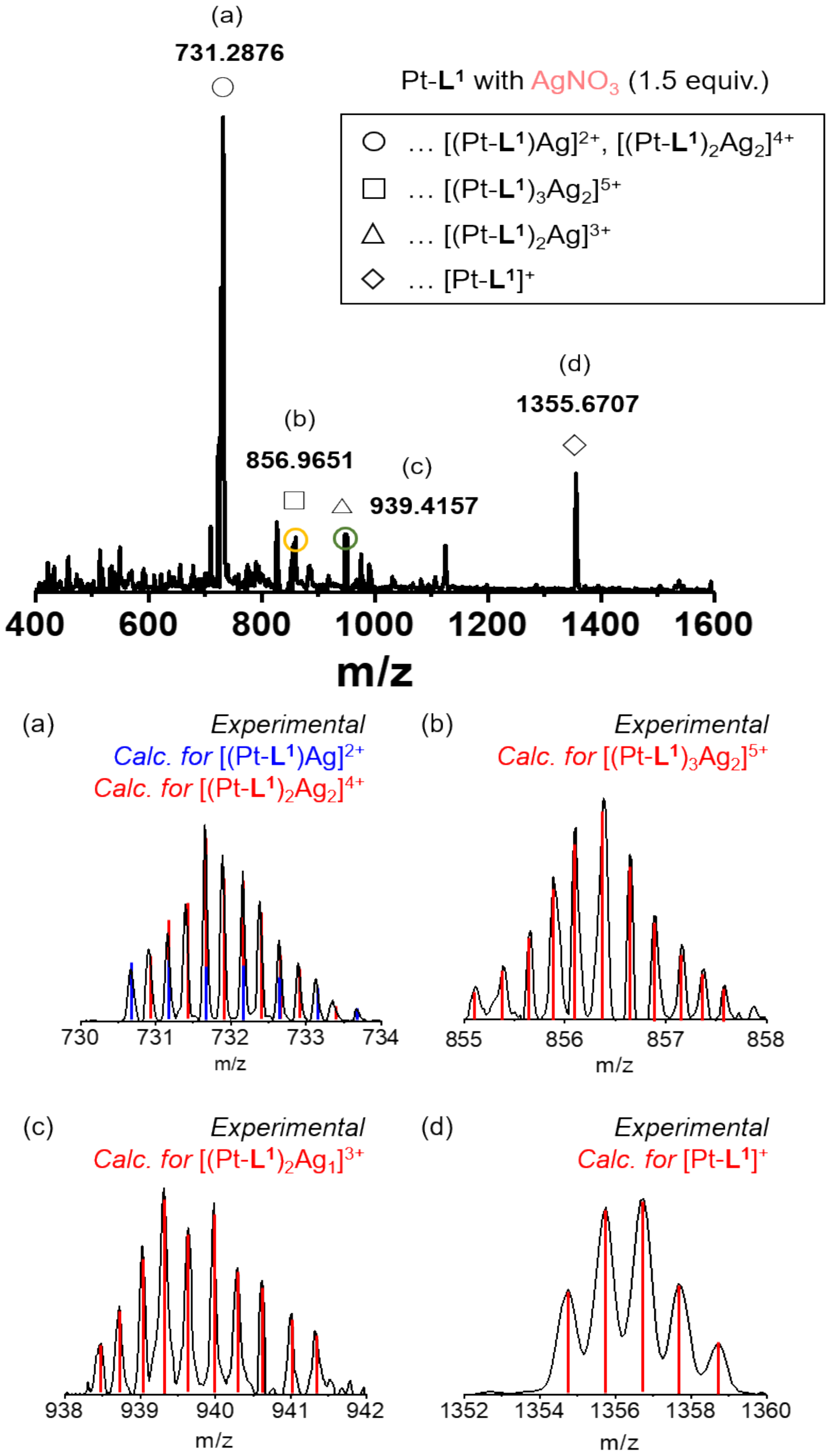

Figure S1. HR-ESI-MS spectra of Pt-**L**^{1} (2.0 mM) in the presence of $AgNO_3$ (1.5 equiv.) in DMSO/H_2O (9:1 v/v) after 24 h aging.

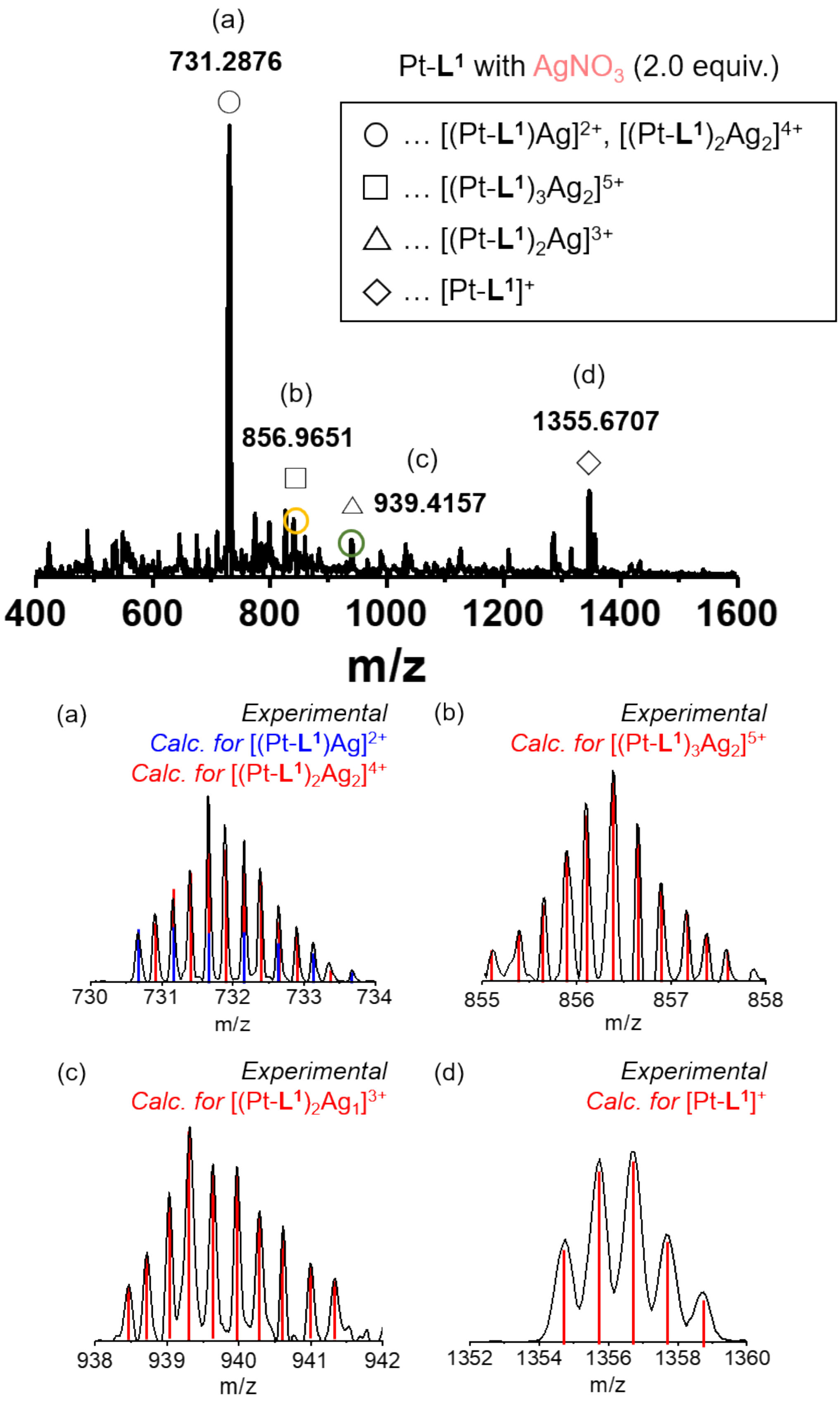

Figure S2. HR-ESI-MS spectra of Pt-**L**[1] (2.0 mM) in the presence of $AgNO_3$ (2.0 equiv.) in DMSO/H_2O (9:1 v/v) after 24 h aging.

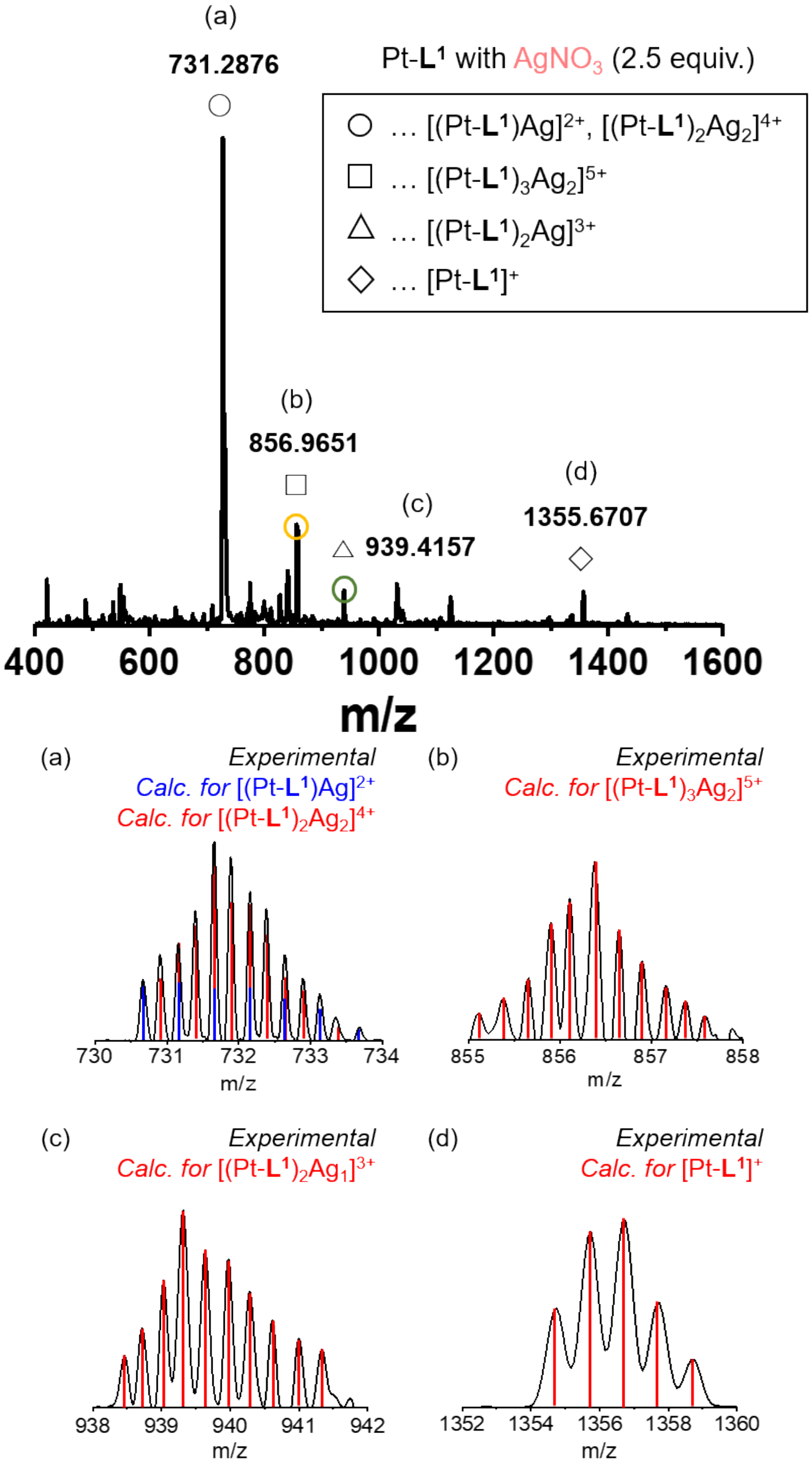

Figure S3. HR-ESI-MS spectra of Pt-**L¹** (2.0 mM) in the presence of $AgNO_3$ (2.5 equiv.) in DMSO/H_2O (9:1 v/v) after 24 h aging.

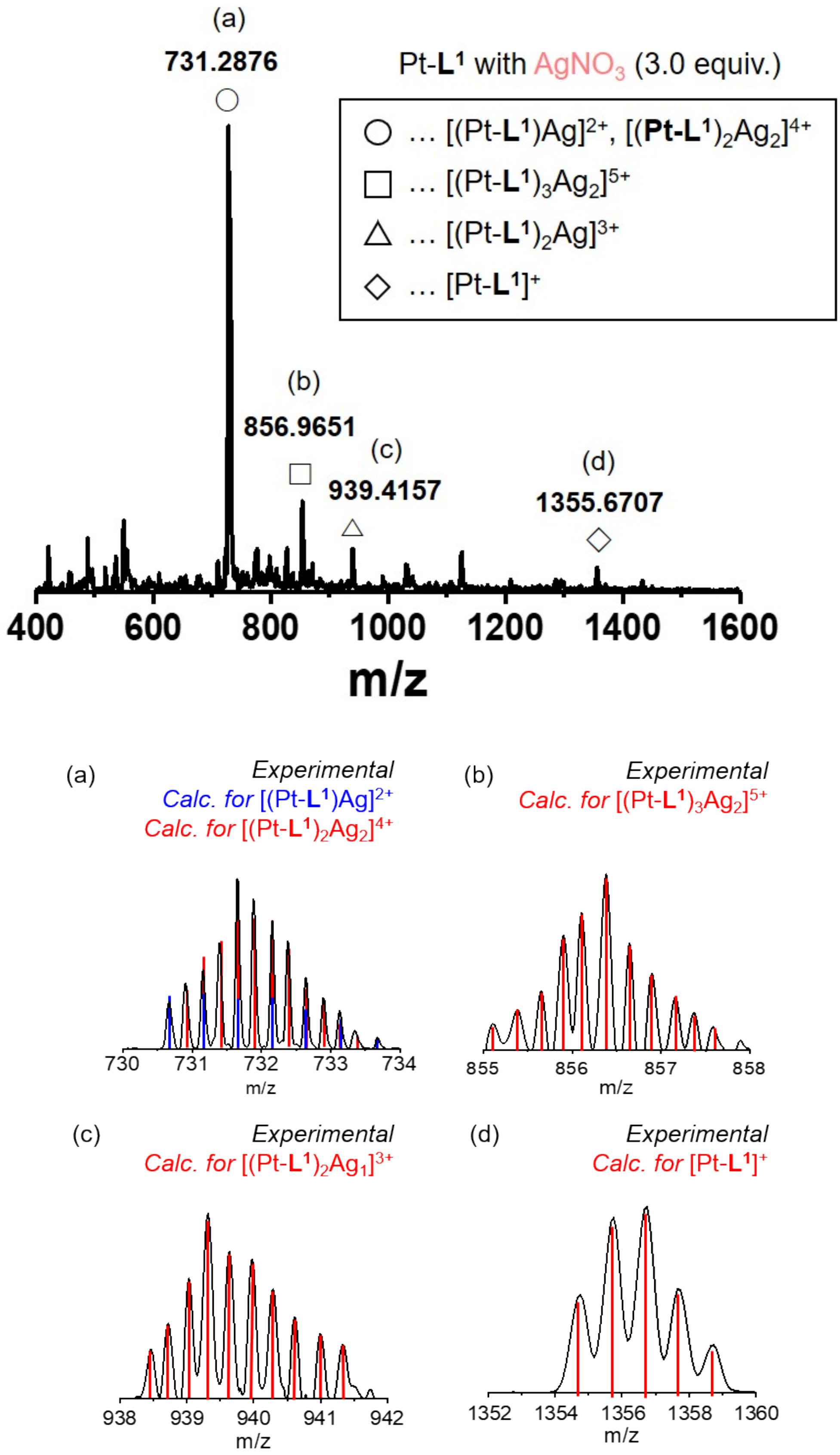

Figure S4. HR-ESI-MS spectra of Pt-**L^1** (2.0 mM) in the presence of $AgNO_3$ (3.0 equiv.) in DMSO/H_2O (9:1 v/v) after 24 h aging.

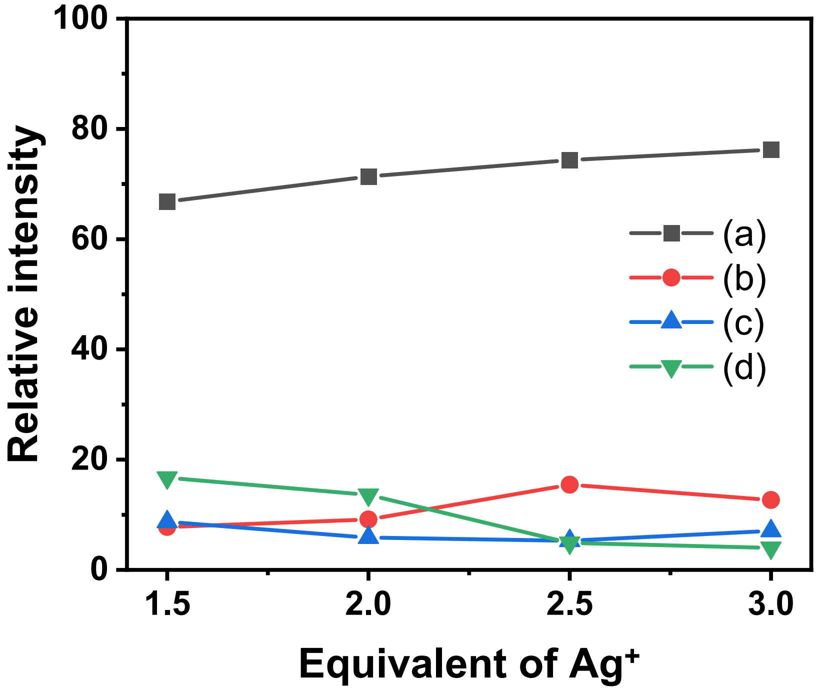

Figure S5. Species distribution for (a) $[(Pt\text{-}\mathbf{L^1})Ag]^{2+}$, $[(Pt\text{-}\mathbf{L^1})_2Ag_2]^{4+}$, (b) $[(Pt\text{-}\mathbf{L^1})_3Ag_2]^{5+}$, (c) $[(Pt\text{-}\mathbf{L^1})_2Ag]^{3+}$ and (d) $[Pt\text{-}\mathbf{L^1}]^{+}$ monitored by HR-ESI-MS spectra of **Pt-L¹** (2 mM) in the presence of different concentrations of $AgNO_3$ at (1.5-3.0 equiv.) in DMSO/H_2O (9:1 v/v) after 24 h aging.

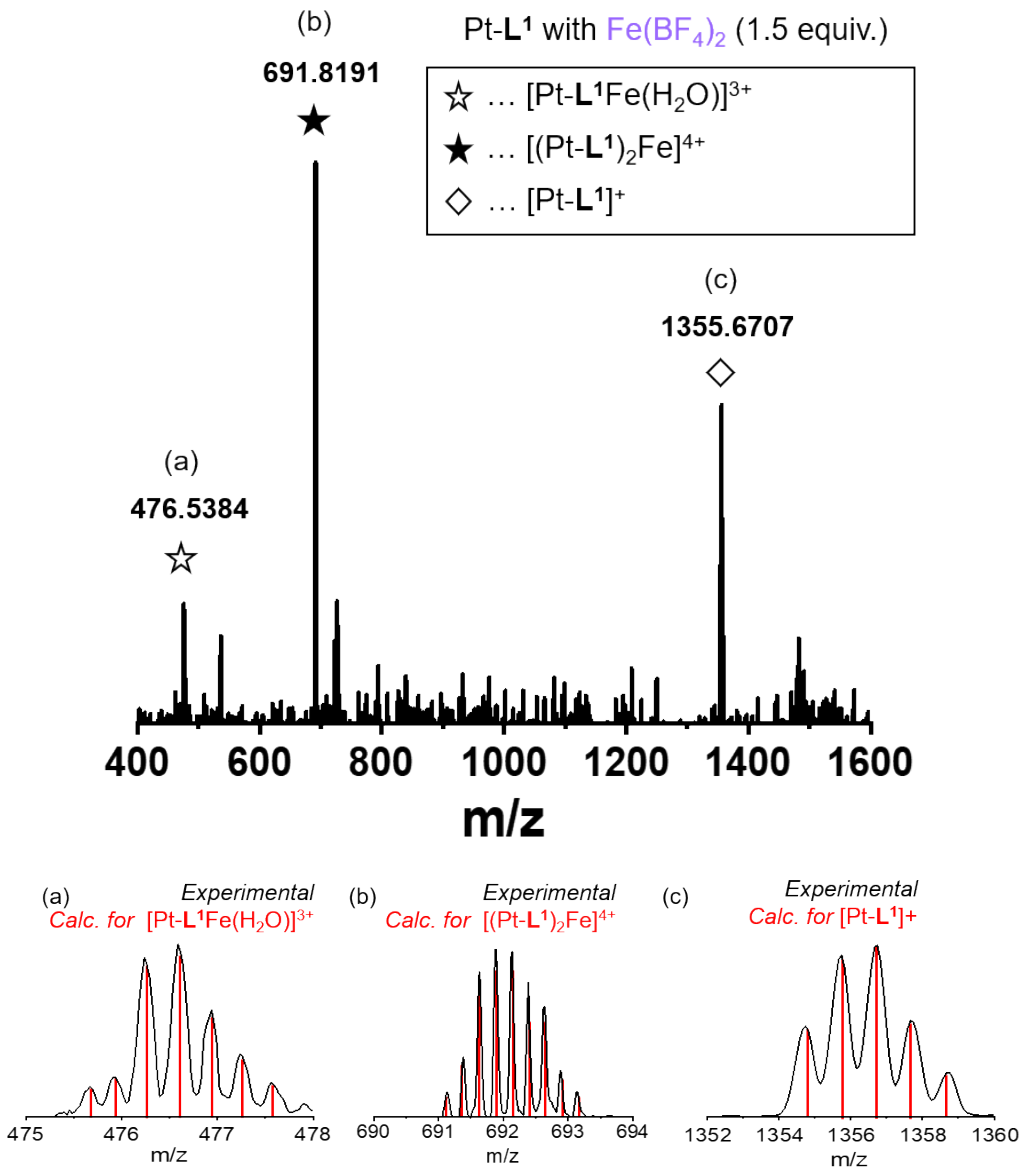

Figure S6. HR-ESI-MS spectra of Pt-**L**¹ (2.0 mM) in the presence of $Fe(BF_4)_2$ (1.5 equiv.) in DMSO/H_2O (9:1 v/v) after 5 h aging.

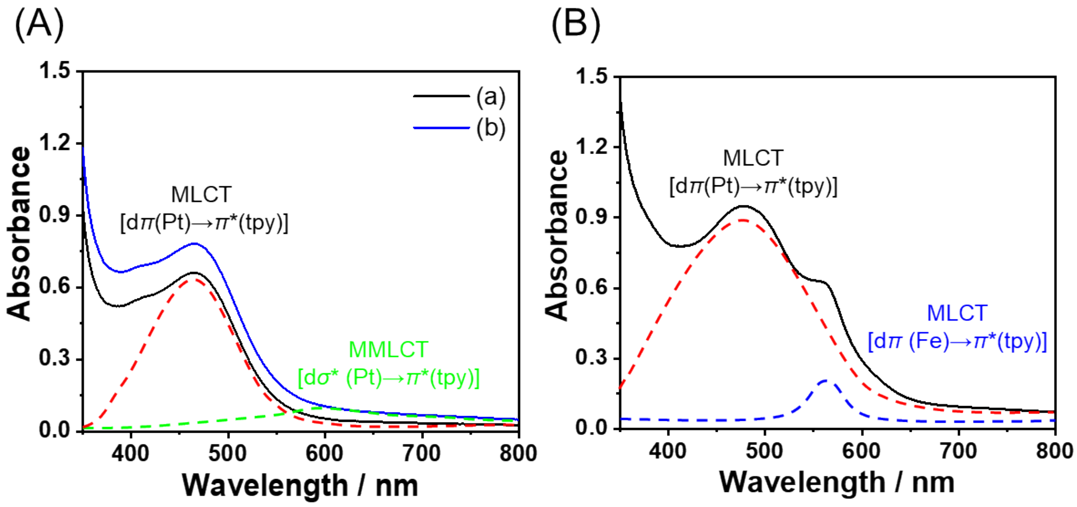

Figure S7. UV spectra of (A) Pt-**L**[1] (2.0 mM) (a) without and with (b) $AgNO_3$ (1.5 equiv.), and (B) $Fe(BF_4)_2$ (1.5 equiv.) in DMSO and H_2O (9:1 v/v). The dotted lines represent Gaussian fits for spectra, with red indicating the MLCT band, green indicating the MMLCT band, and blue indicating the MLCT band from Figures S7A and S7B, respectively.

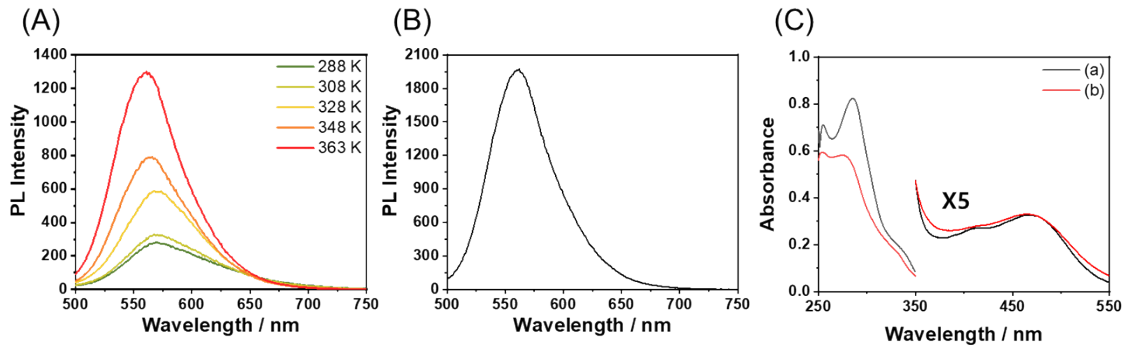

Figure S8. (A) PL spectra (λ_{ex} = 420 nm) of Pt-**L**[1] (2.0 mM) in DMSO and H_2O (9:1 v/v) by the increase of temperature. (B) PL spectrum (λ_{ex} = 420 nm) of Pt-**L**[1] (2.0 mM) in pure DMSO at 293 K. (C) UV-vis spectra of Pt-**L**[1] (2.0 mM) in (a) pure DMSO and (b) DMSO and H_2O (9:1 v/v) Cell pathlength: 0.1 mm.

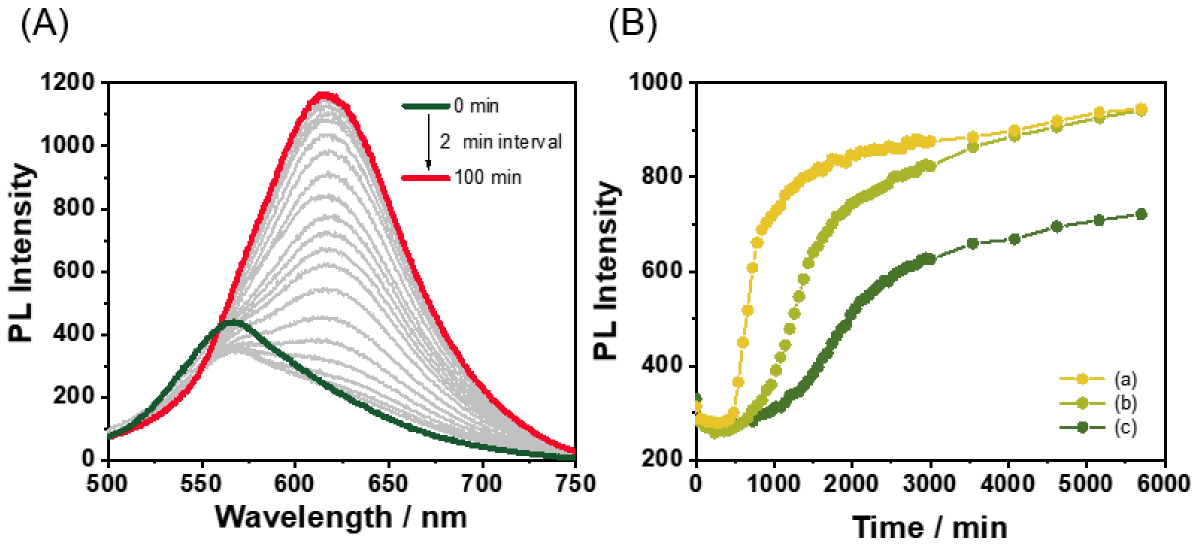

Figure S9. (A) Time-dependent PL spectra (λ_{ex} = 420 nm) of Pt-**L**[1] (2.0 mM) in the presence of $AgPF_6$ (1.5 equiv.) in DMSO/H_2O (9:1 v/v) at 20 °C. (B) Plots of PL intensity (at 620 nm) vs. times for different silver(I) salts; (a) $AgNO_3$, (b) $AgPF_6$, and (c) $AgBF_4$.

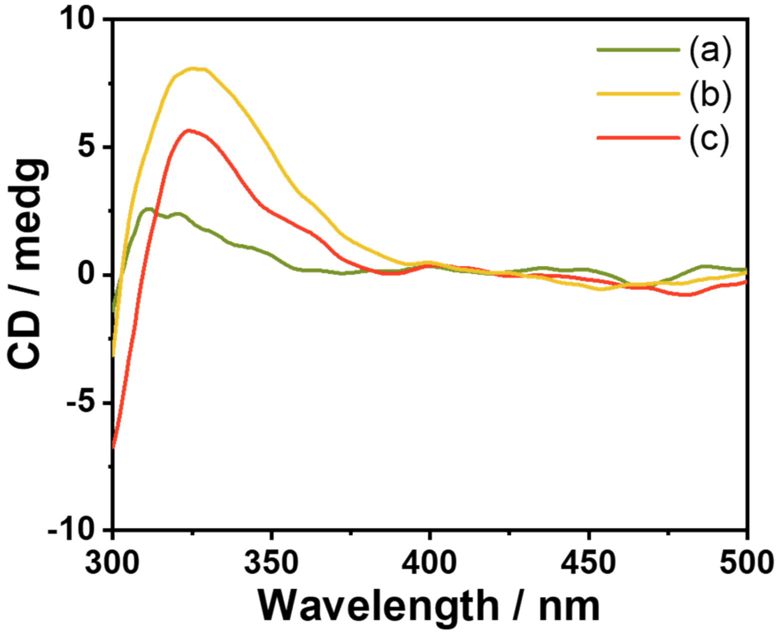

Figure S10. CD spectra of Pt-**L**[1] (2.0 mM) (a) without and with (b) $AgNO_3$ (1.5 equiv.) and (c) $Fe(BF_4)_2$ (1.5 equiv.) in DMSO/H_2O (9:1 v/v) at 293 K.

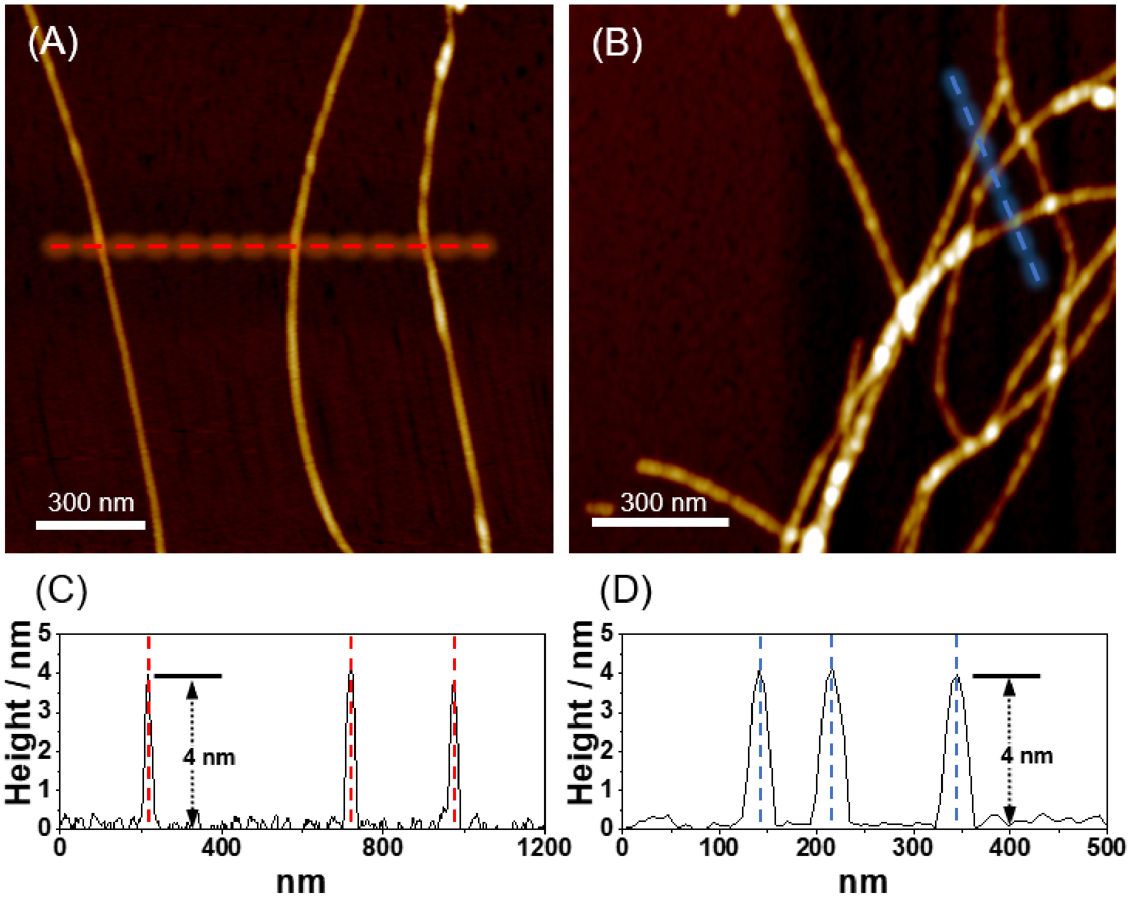

Figure S11. AFM images and height profiles of SP-**II** obtained from Pt-$\mathbf{L}^1$ (2.0 mM) in the presence of $AgNO_3$ (1.5 equiv.) in DMSO/H_2O (9:1 v/v).

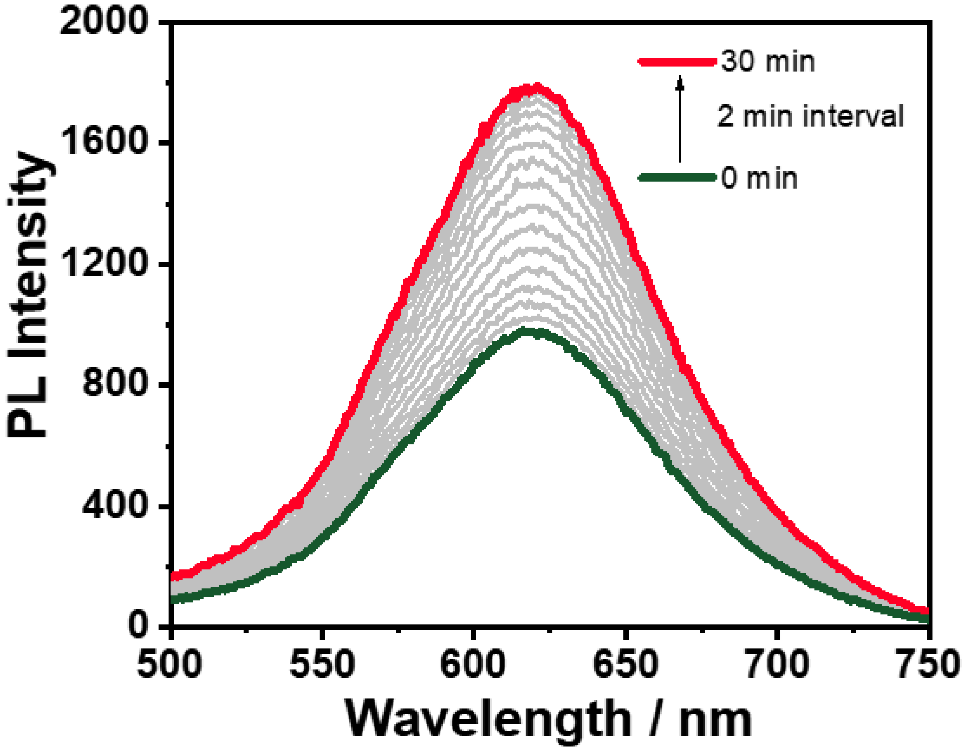

Figure S12. Time-dependent PL spectra (λ_{ex} = 420 nm) upon addition of NaCl (6.0 equiv.) to SP-**II** (2.0 mM) obtained from Pt-**L**[1] (2.0 mM) in the presence of $AgNO_3$ (1.5 equiv.) in DMSO/H_2O (9:1 v/v) at 20 °C.

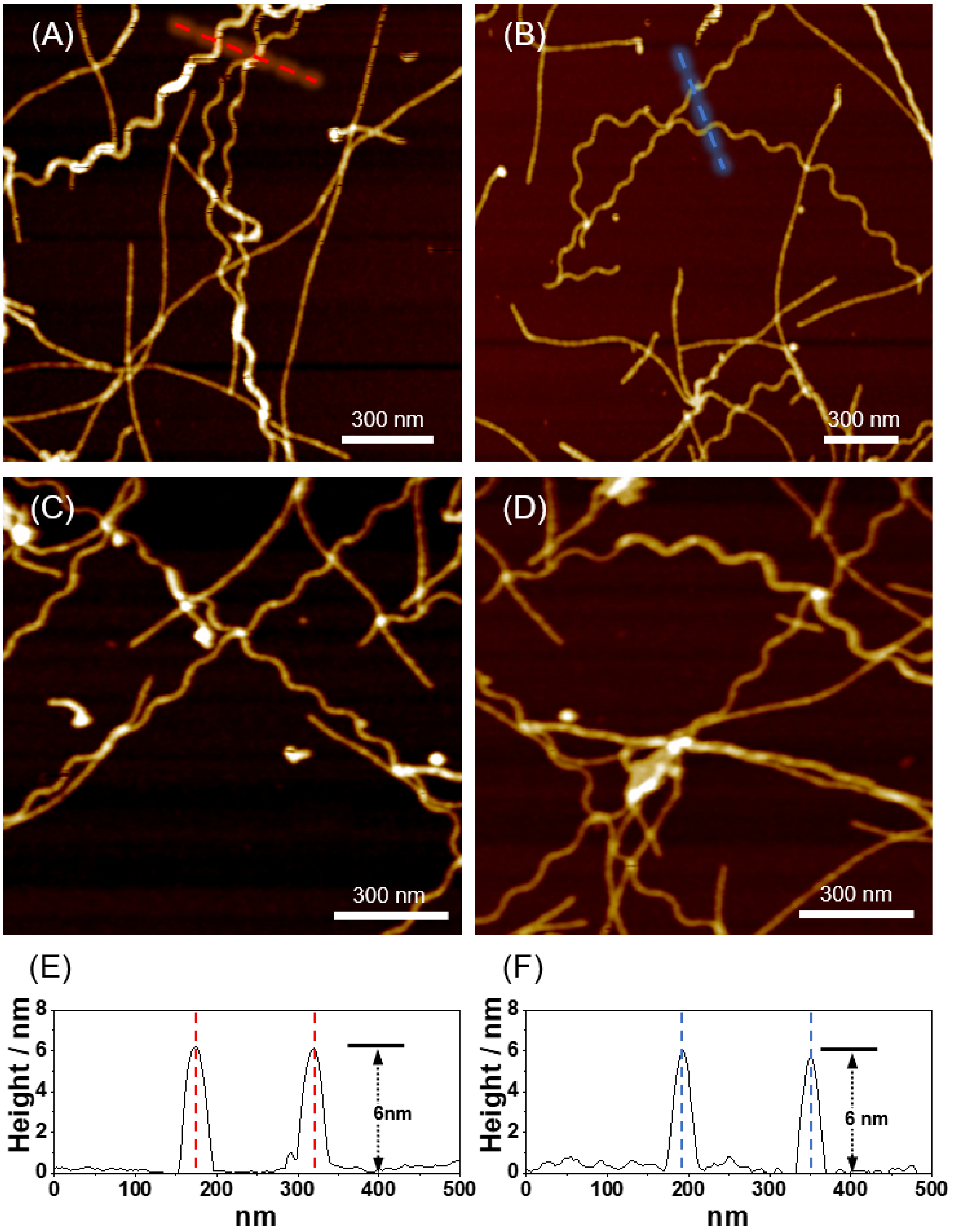

Figure S13. AFM images and height profiles upon addition of NaCl (6 equiv.) to SP-**II** obtained from Pt-**L**[1] (2.0 mM) in the presence of $AgNO_3$ (1.5 equiv.) in DMSO/H_2O (9:1 v/v).

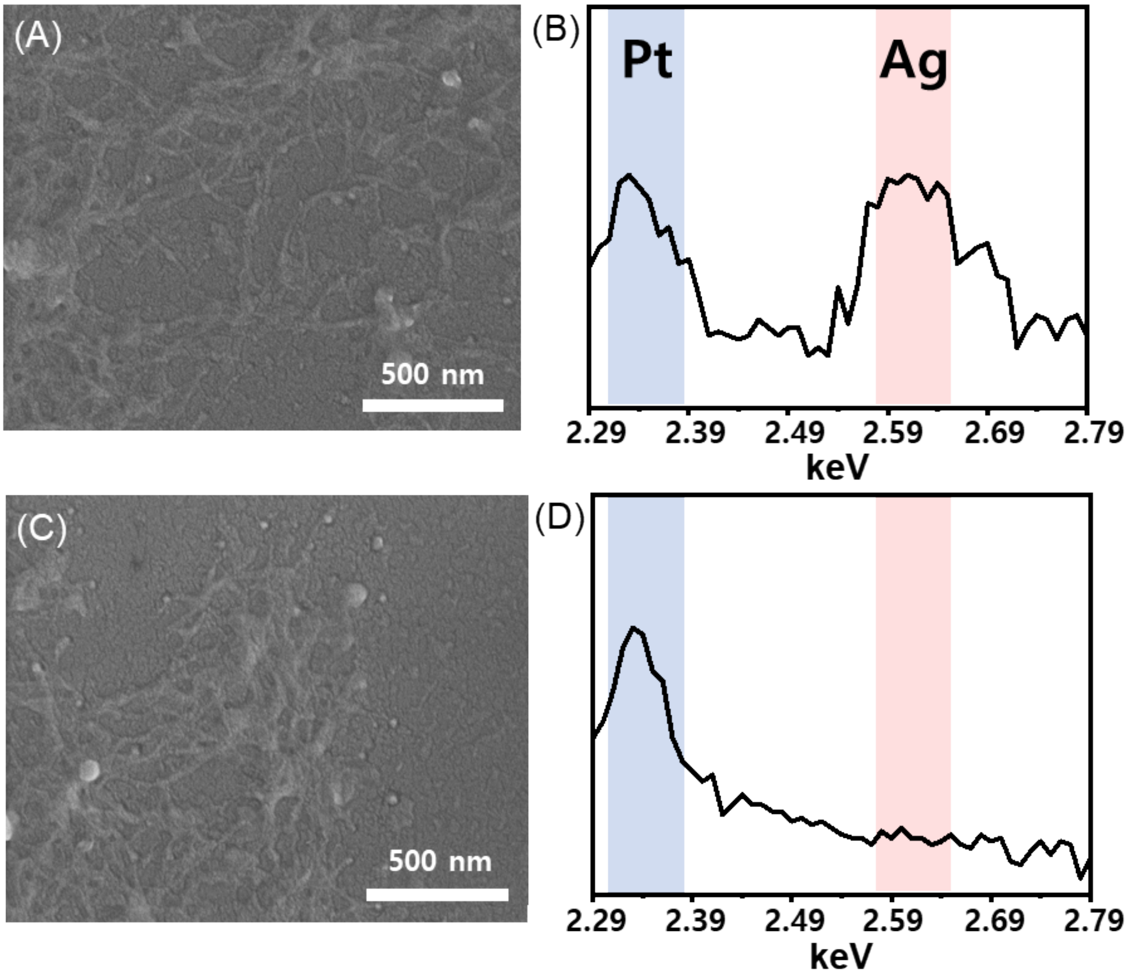

Figure S14. SEM images of (A) SP-II and (C) SP-III. EDS spectra of (B) SP-II and (D) SP-III. SP-III was obtained from SP-II by treatment of NaCl (6.0 equiv.).

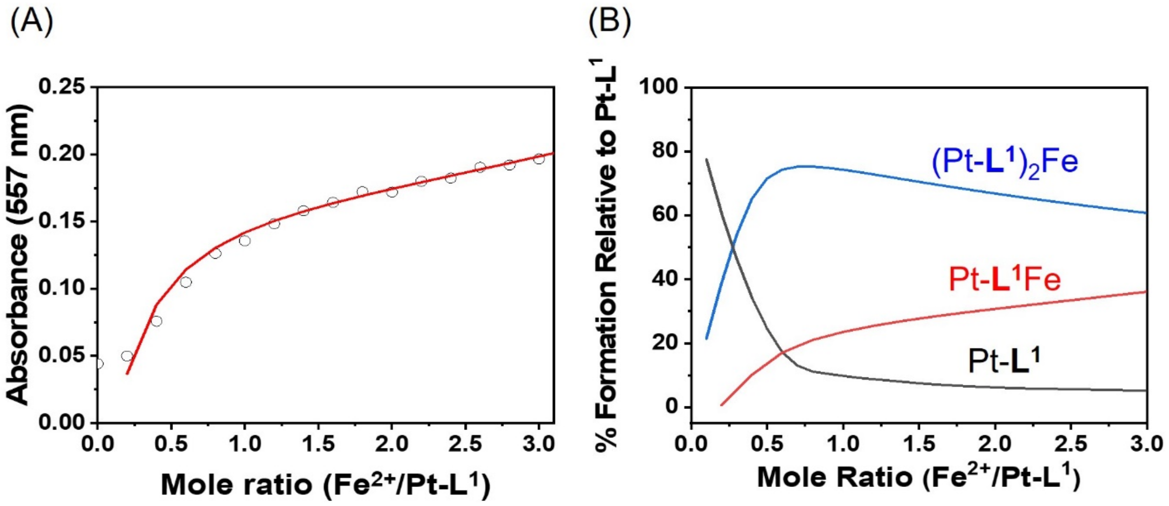

Figure S15. Fitting of the UV-vis titration data to determine the stability constants of the Fe^{2+}-(Pt-**L¹**) complexation with HyperSpec software by employing the multiple binding model including 1:1 and 2:1 ratios: (A) absorbance changes (at 557 nm) of Pt-**L¹** (0.02 mM) as a function of the mole ratio (Fe^{2+}/Pt-**L¹**, ○: experimental points, solid line: theoretical fit) at 293 K. (B) Species distribution plots. The stability constant was determined by monitoring the MLCT band corresponding [dπ(Fe)→π*(py)] transition.

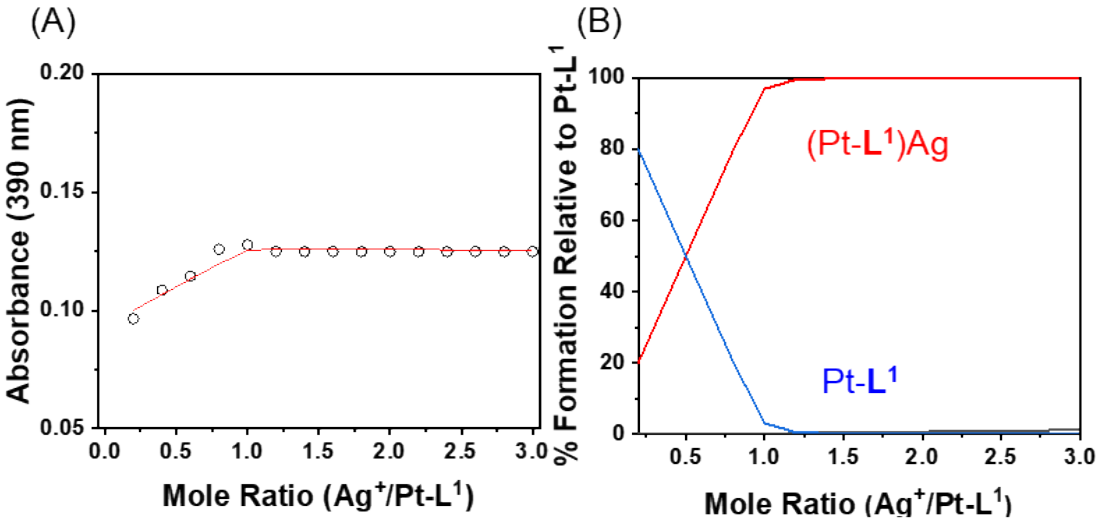

Figure S16. Fitting of the UV-vis titration data to determine the stability constants of the Ag^{+}-(Pt-**L**^{1}) complexation with HyperSpec software by employing the multiple binding model including 1:1 ratio: (A) absorbance changes (at 390 nm) of Pt-**L**^{1} (0.02 mM) as a function of the mole ratio (Ag^{+}/Pt-**L**^{1}, ○: experimental points, solid line: theoretical fit) in DMSO/H_2O (9:1 v/v) at 293 K and (B) species distribution plots.

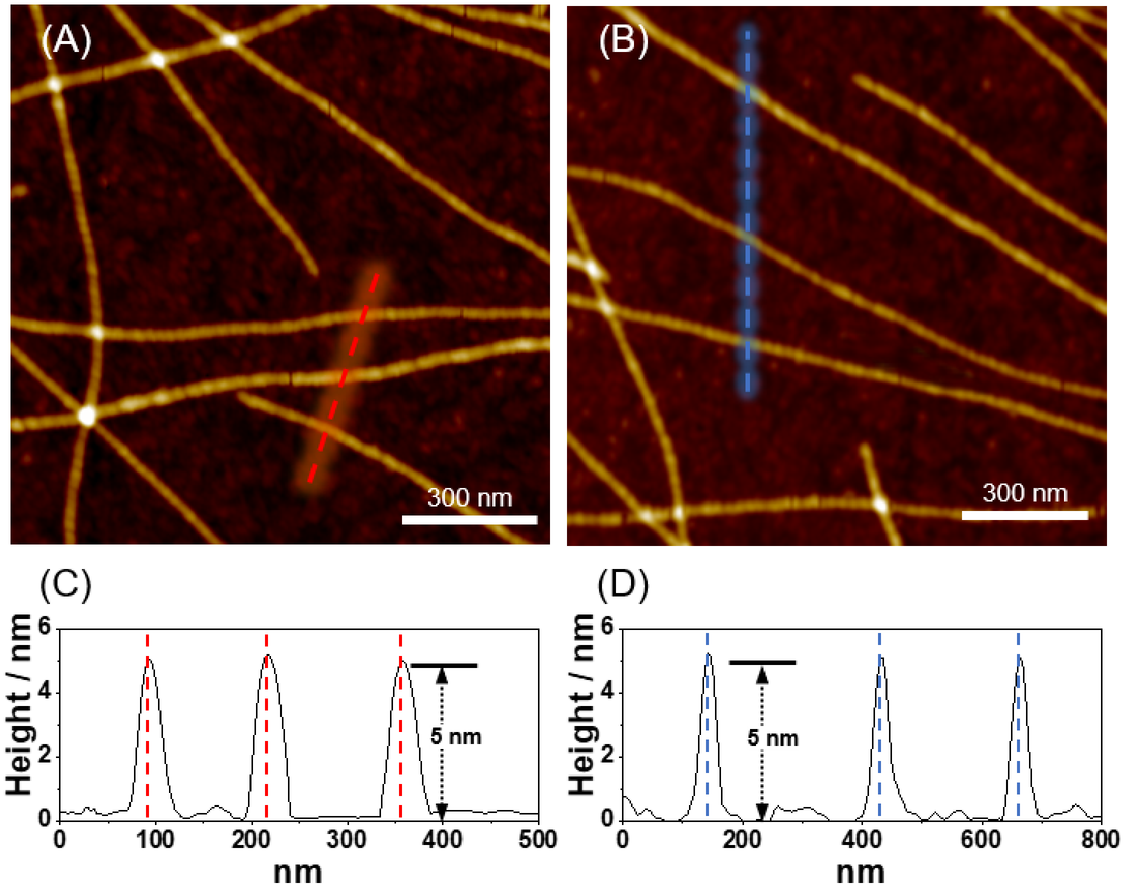

Figure S17. AFM images and height profiles of SP-**IV** obtained from Pt-**L**[1] (2.0 mM) in the presence of $Fe(BF_4)_2$ (1.5 equiv.) in DMSO/H_2O (9:1 v/v).

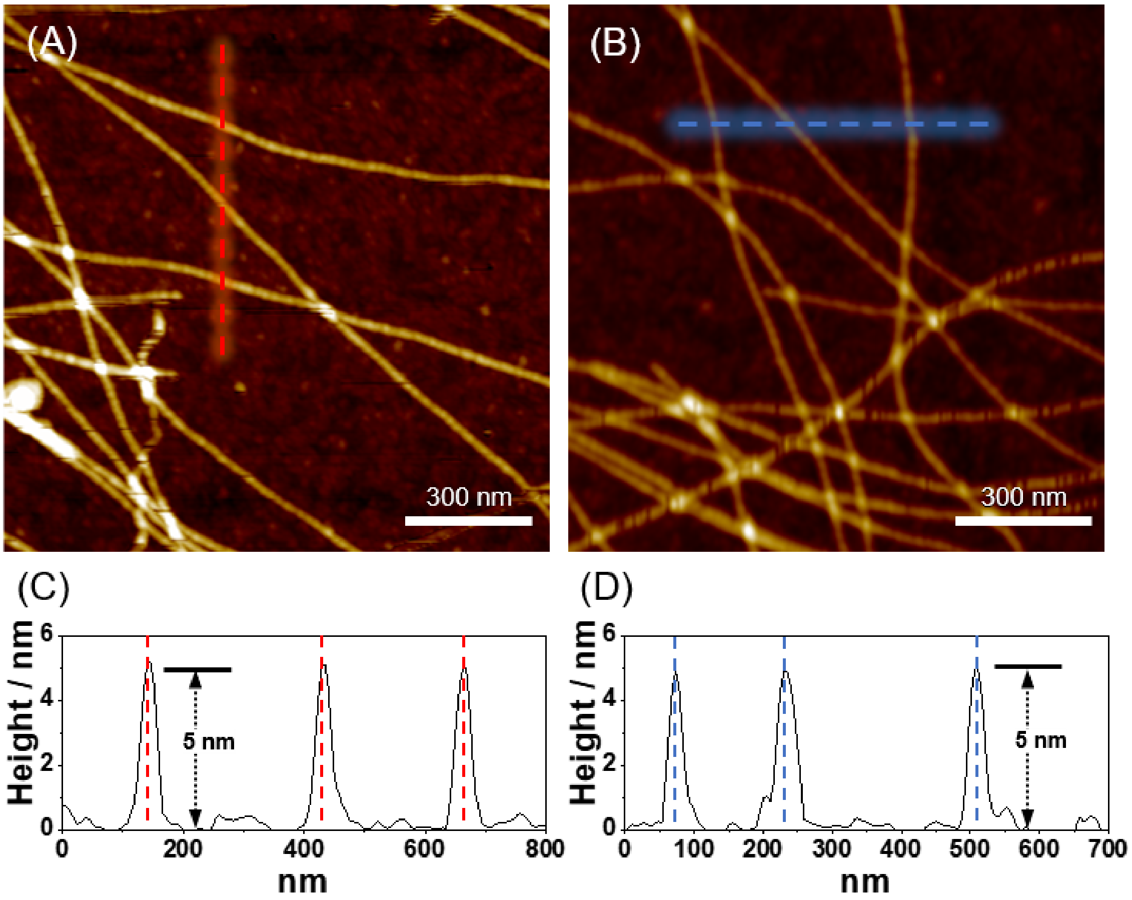

Figure S18. AFM images and height profiles upon addition of $Fe(BF_4)_2$ (1.5 eq) to SP-**II** obtained from Pt-**L**[1] (2.0 mM) in the presence of $AgNO_3$ (1.5 equiv.) in DMSO/H_2O (9:1 v/v).

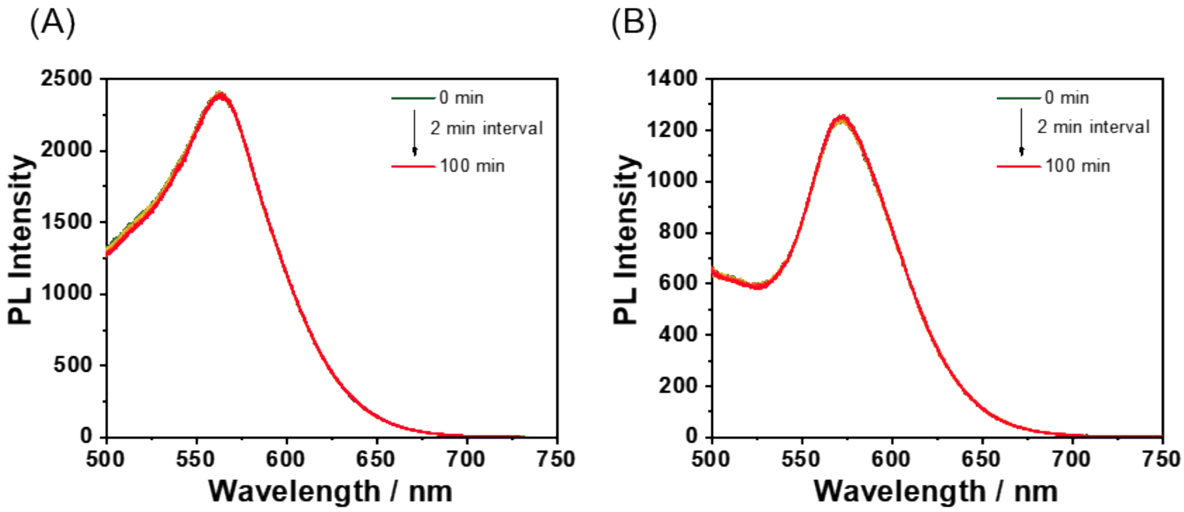

Figure S19. Time-dependent PL spectra (λ_{ex} = 420 nm) of (A) Pt-$\mathbf{L}^2$ (2.0 mM) and (B) Pt-$\mathbf{L}^3$ (2.0 mM) in DMSO/H_2O (9:1 v/v) at 293 K.

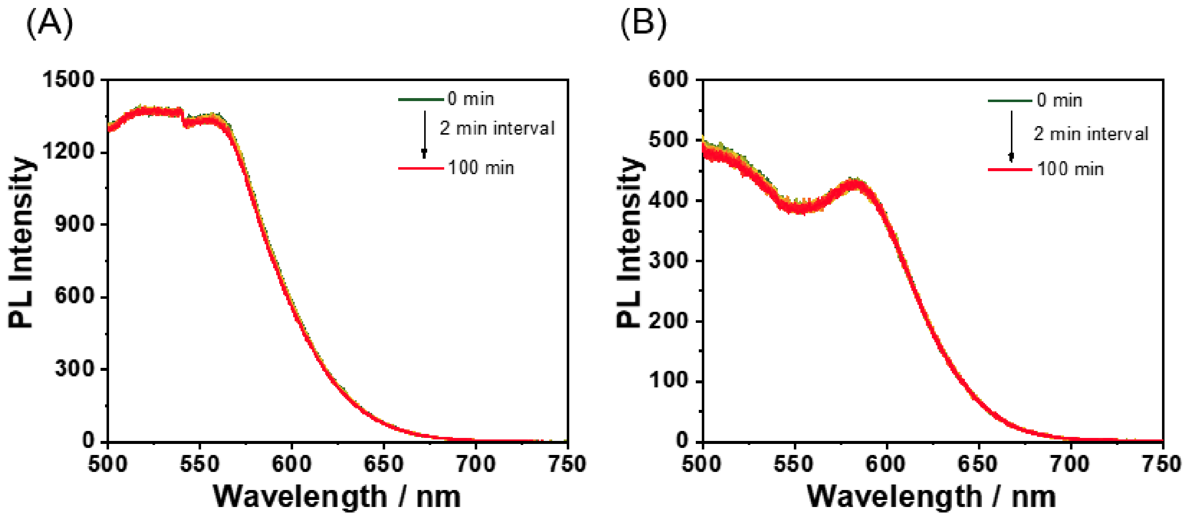

Figure S20. Time-dependent PL spectra (λ_{ex} = 420 nm) of Pt-**L**^{2} (2.0 mM) in the presence of (A) $AgNO_3$ (1.5 equiv.) and (B) $Fe(BF_4)_2$ (1.5 equiv.) in DMSO/H_2O (9:1 v/v) at 293 K.

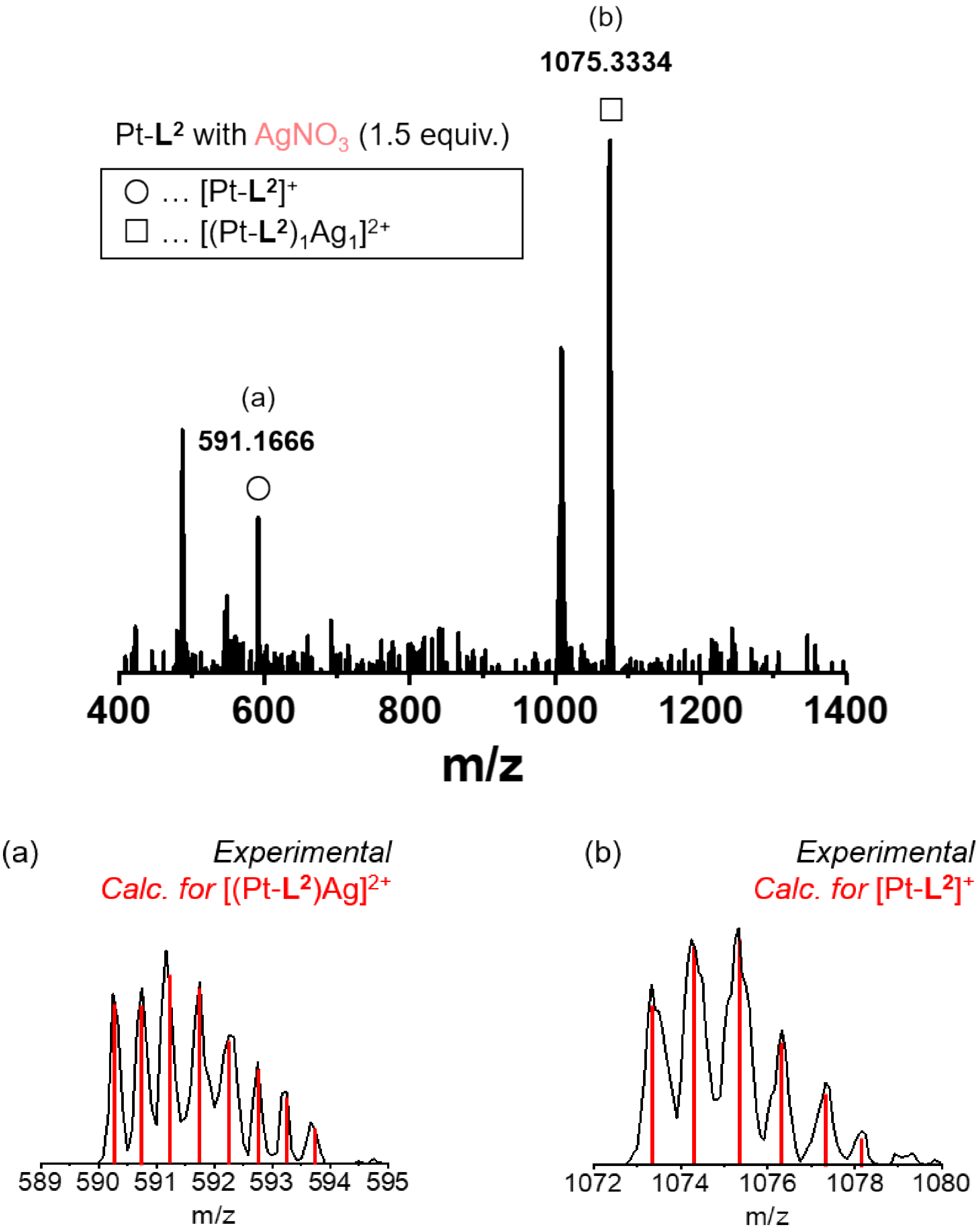

Figure S21. ESI-MS spectrum of Pt-**L**^{2} (2.0 mM) in the presence of $AgNO_3$ in DMSO/H_2O (9:1 v/v).

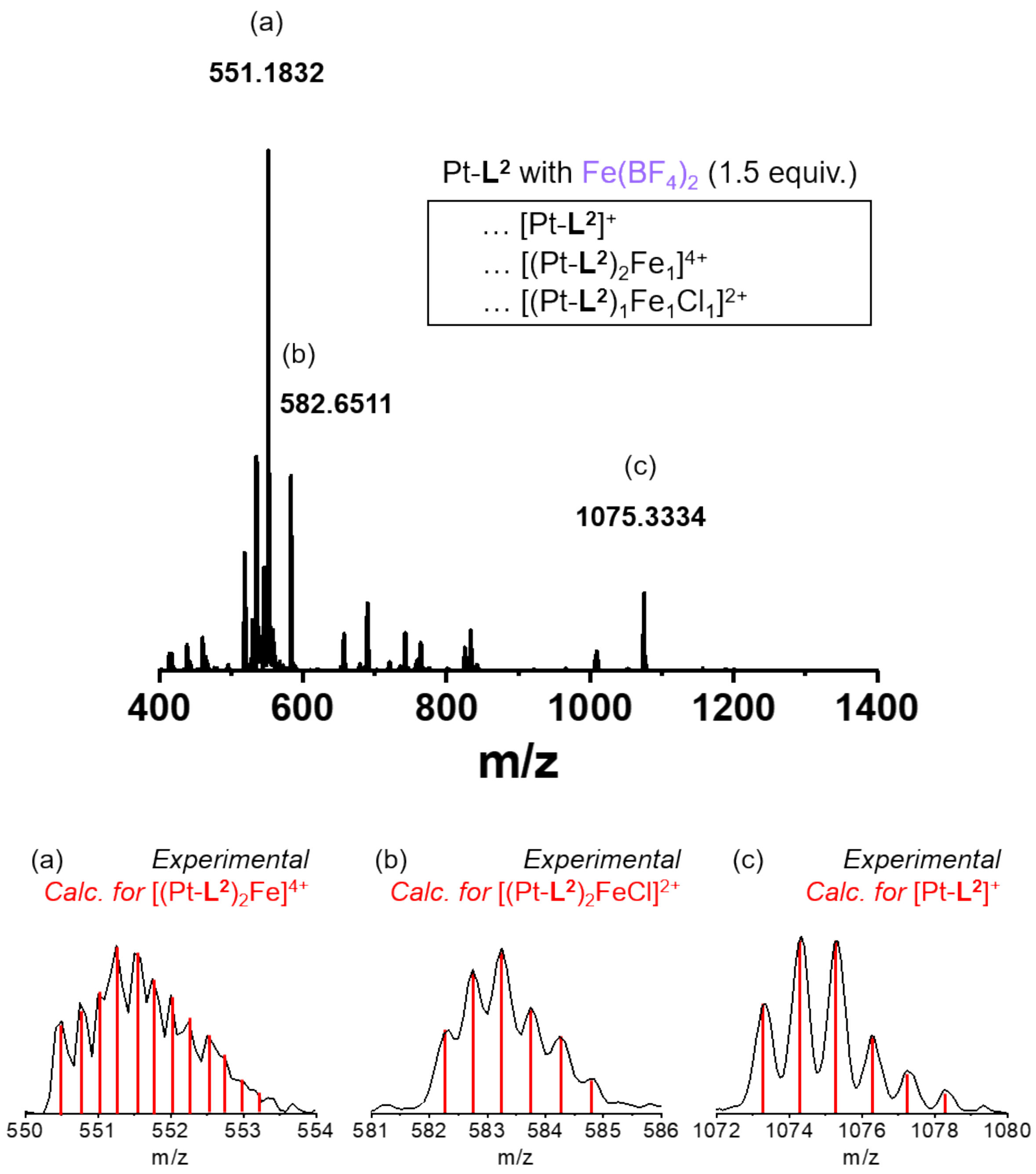

Figure S22. ESI-MS spectrum of Pt-**L**^{2} (2.0 mM) in the presence of $Fe(BF_4)_2$ in DMSO/H_2O (9:1 v/v).

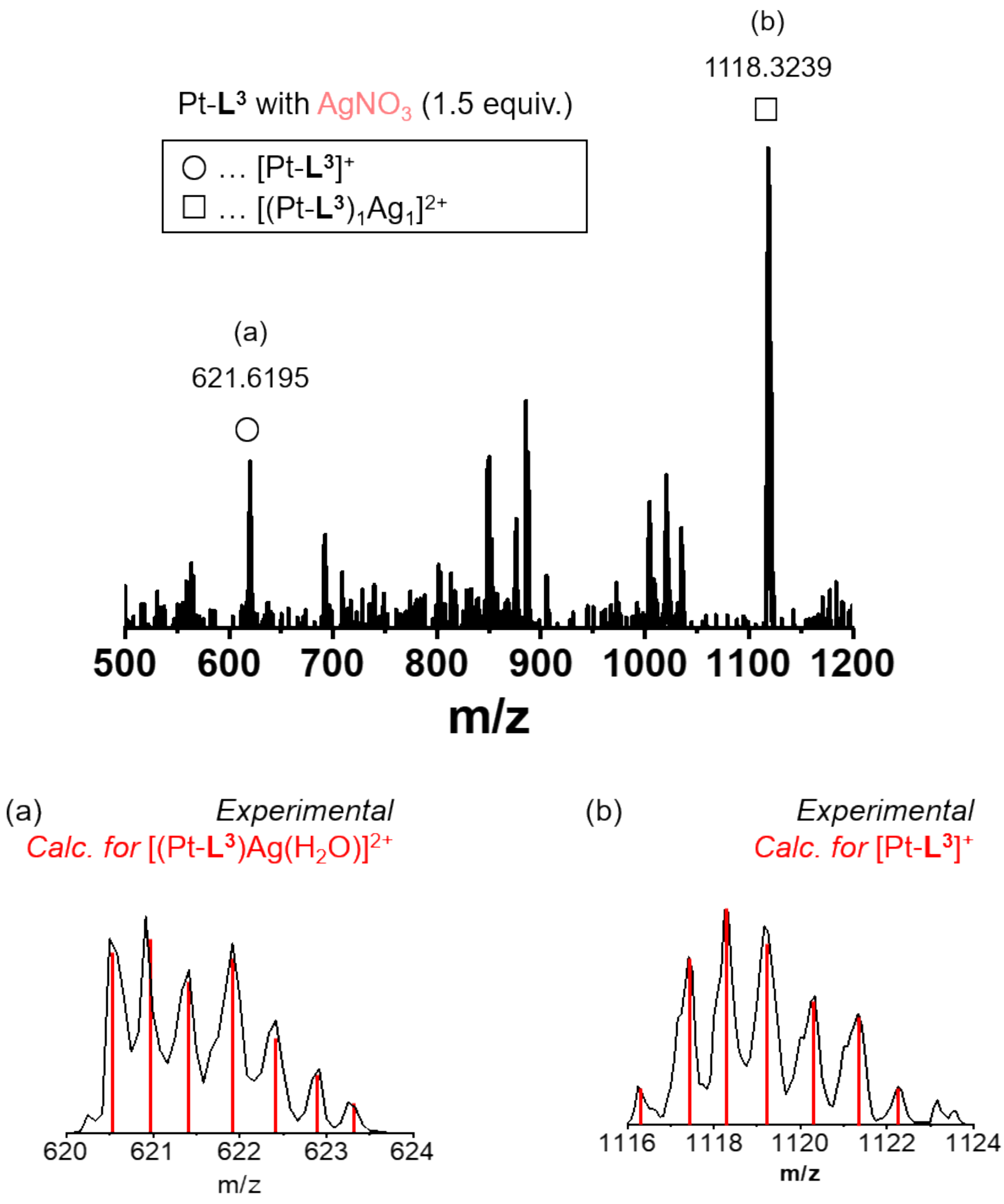

Figure S23. ESI-MS spectrum of Pt-**L**^{3} (2.0 mM) in the presence of $AgNO_3$ (1.5 equiv.) in DMSO/H_2O (9:1 v/v).

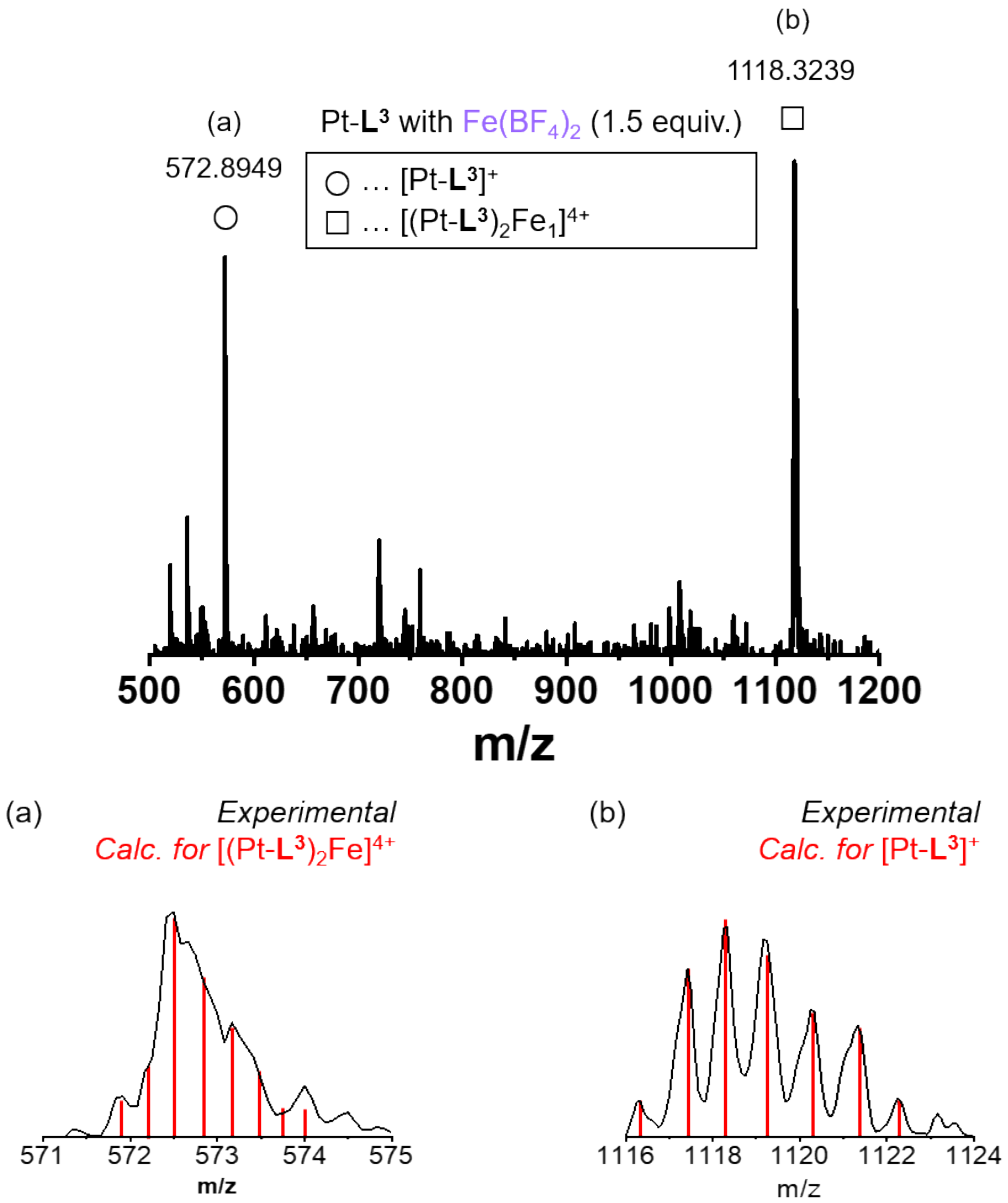

Figure S24. ESI-MS spectrum of Pt-**L**³ (2.0 mM) in the presence of $Fe(BF_4)_2$ (1.5 equiv.) in DMSO/H_2O (9:1 v/v).

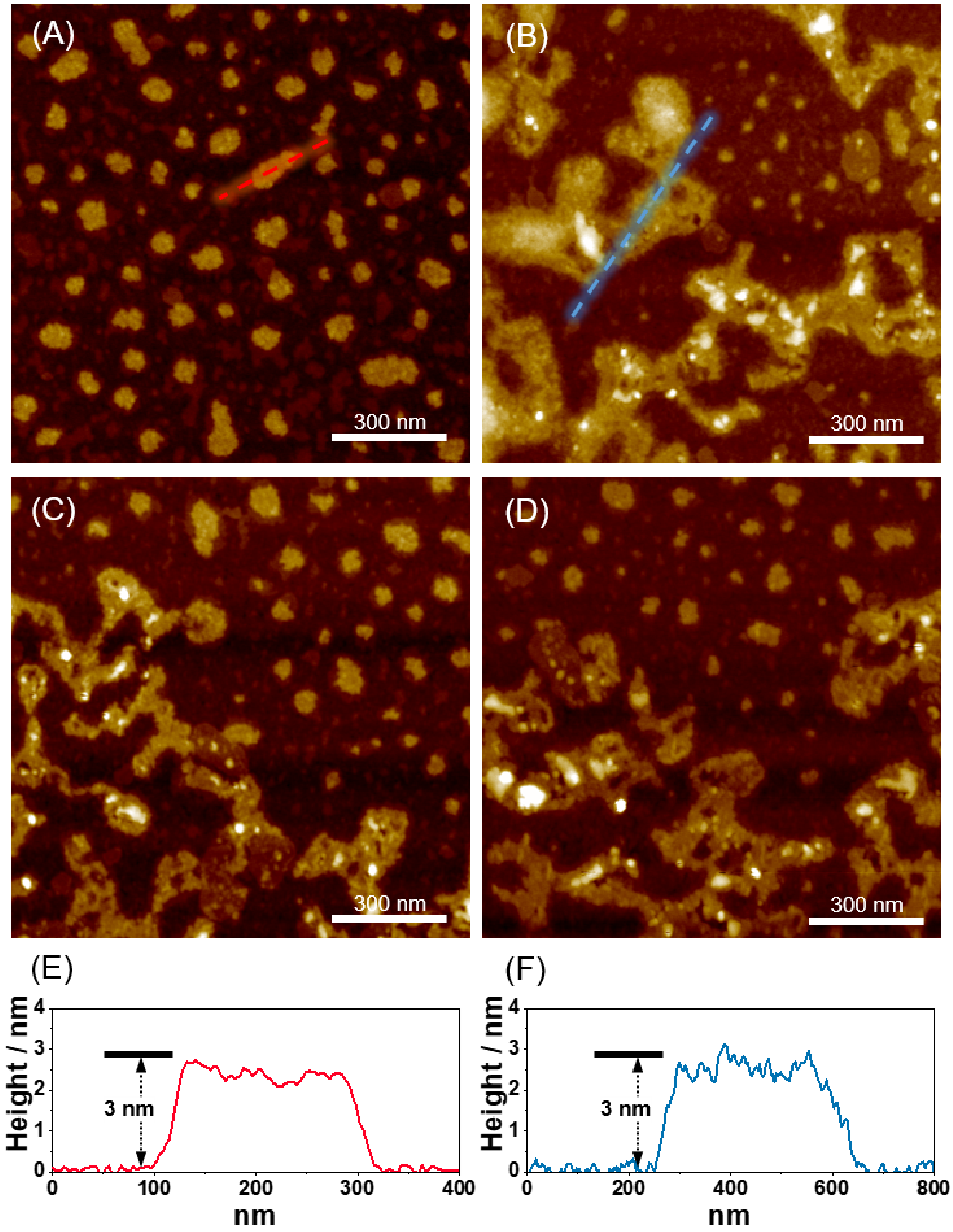

Figure S25. AFM images and height profiles obtained from Pt-**L**^{2} (2.0 mM) (A and B) in the absence and presence of (C) $AgNO_3$ (1.5 equiv.) and (D) $Fe(BF_4)_2$ (1.5 equiv.) in DMSO/H_2O (9:1 v/v).

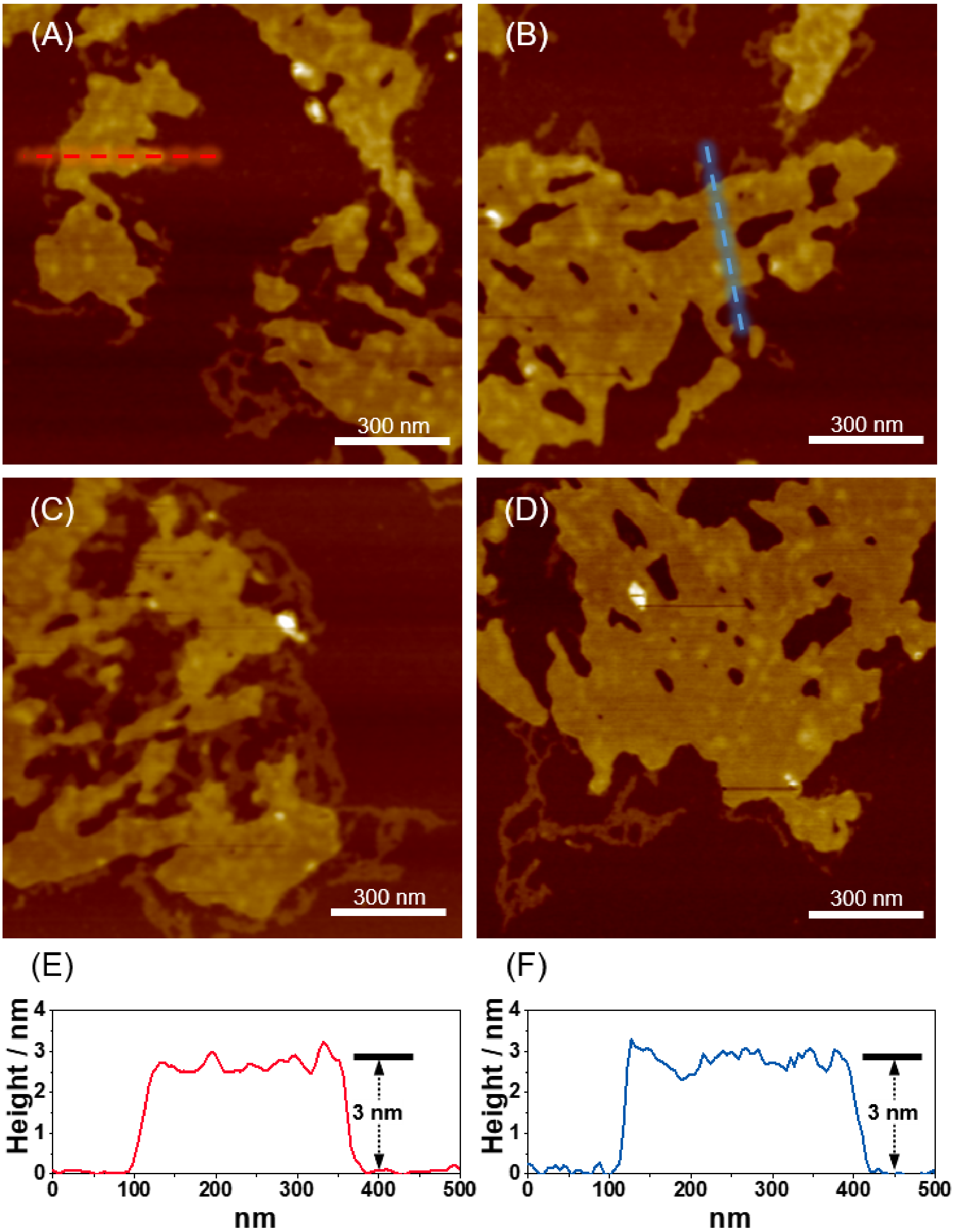

Figure S26. AFM images and height profiles obtained from Pt-**L**^{3} (2.0 mM) (A and B) in the absence and presence of (C) $AgNO_3$ (1.5 equiv.) and (D) $Fe(BF_4)_2$ (1.5 equiv.) in DMSO/H_2O (9:1 v/v).

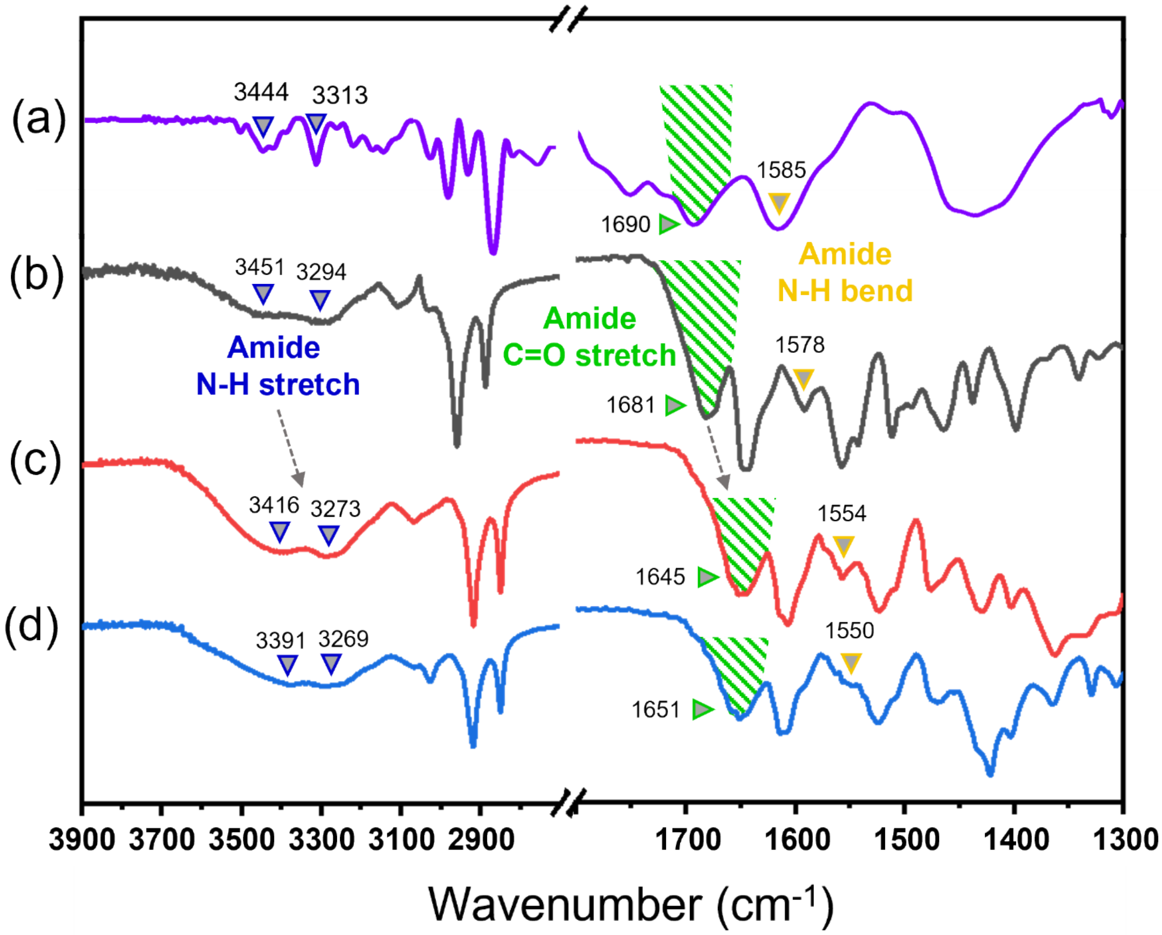

Figure S27. IR spectra of (a) Pt-**L**[1] in pure DMSO, (b) SP-**I**, (c) SP-**II**, and (d) SP-**IV** in DMSO/H_2O (9:1 v/v). The concentration of each SP is 3.0 mM.

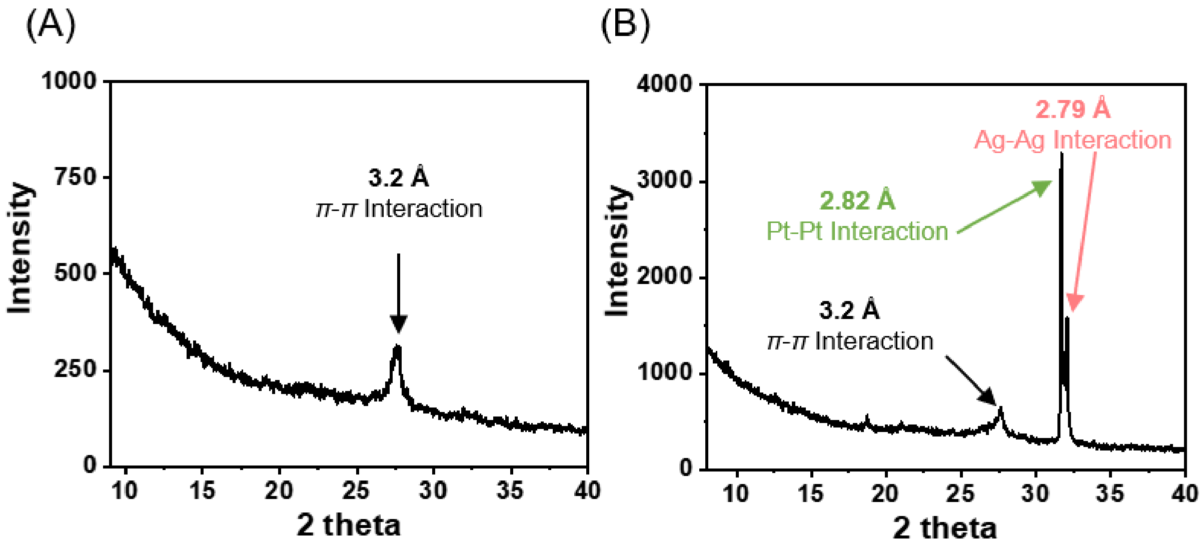

Figure S28. Wide-angle X-ray diffraction (WXRD) patterns of (A) SP-**I** and (B) SP-**II**. The concentration of each SP is 3.0 mM.

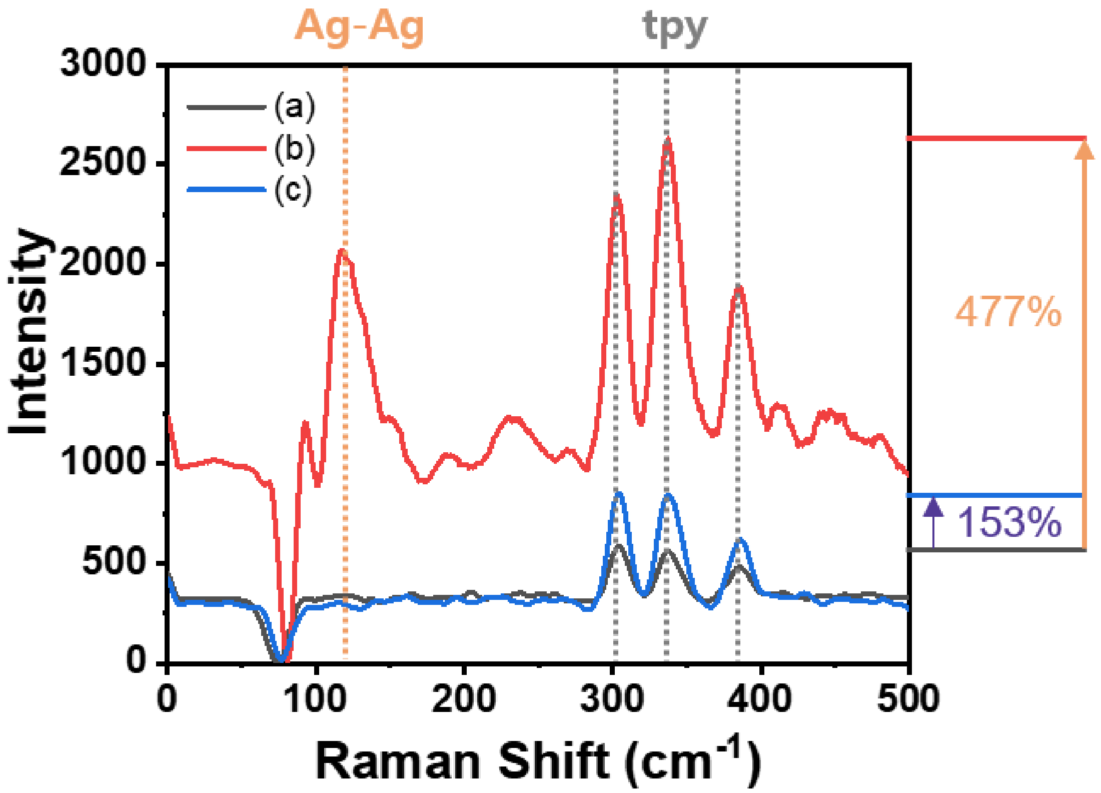

Figure S29. Raman spectra of (a) Pt-**L**[1] (3.0 mM, black color), (b) Pt-**L**[1] (3.0 mM) with $AgNO_3$ (1.5 equiv., red color), and (c) Pt-**L**[1] (3.0 mM, with $Fe(BF_4)_2$ (1.5 equiv. blue color).

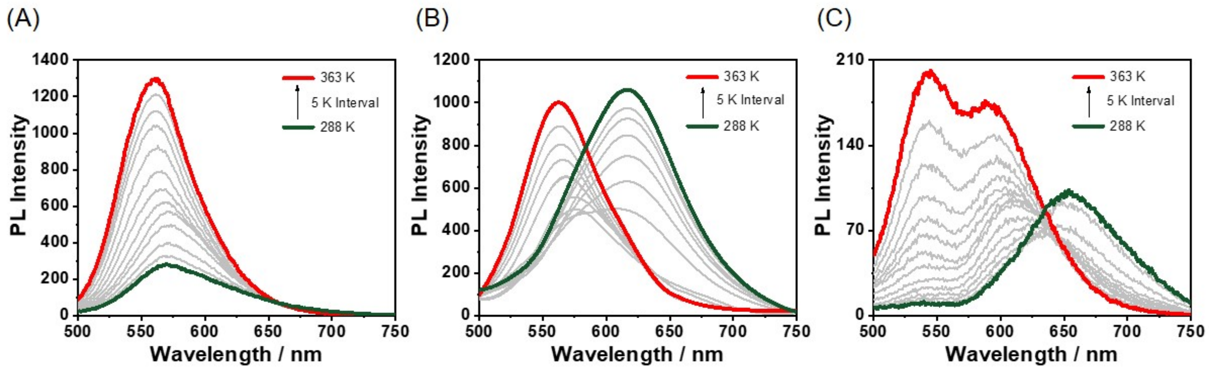

Figure S30. Temperature-dependent PL spectra (λ_{ex} = 420 nm) of (A) Pt-$\mathbf{L^1}$ (2.0 mM), (B) Pt-$\mathbf{L^1}$ (2.0 mM) + $AgNO_3$ (1.5 equiv.), and (C) Pt-$\mathbf{L^1}$ (2.0 mM) + $Fe(BF_4)_2$ (1.5 equiv.) in DMSO/H_2O (9:1 v/v).

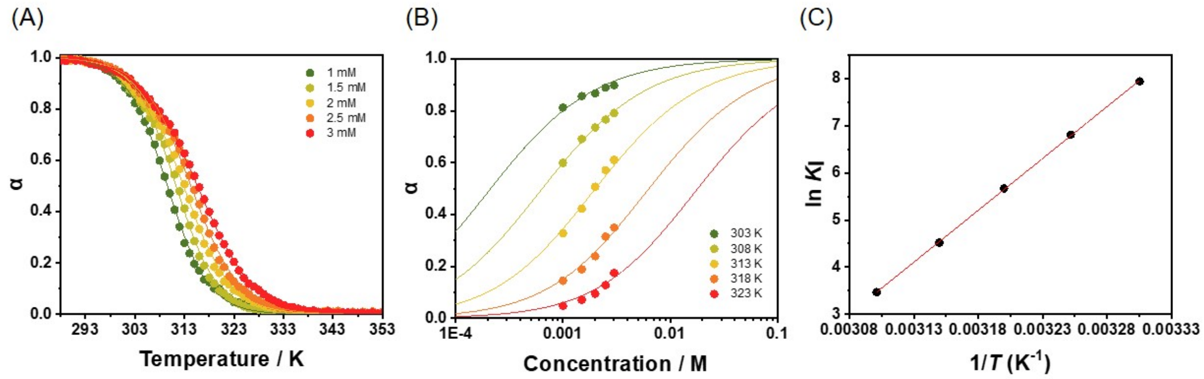

Figure S31. (A) Degree of aggregation (α, at 620 nm) as a function of temperature in different concentrations of Pt-**L**[1] (1.0-3.0 mM) in DMSO/H_2O (9:1 v/v): heating rate; 1 K min^{-1}. (B) Degree of aggregation as a function of pristine Pt-**L**[1] concentration at different temperatures in DMSO/H_2O (9:1 v/v). (C) Natural logarithm of the reciprocal C_T as a function of the reciprocal T_e showing a linear relationship.

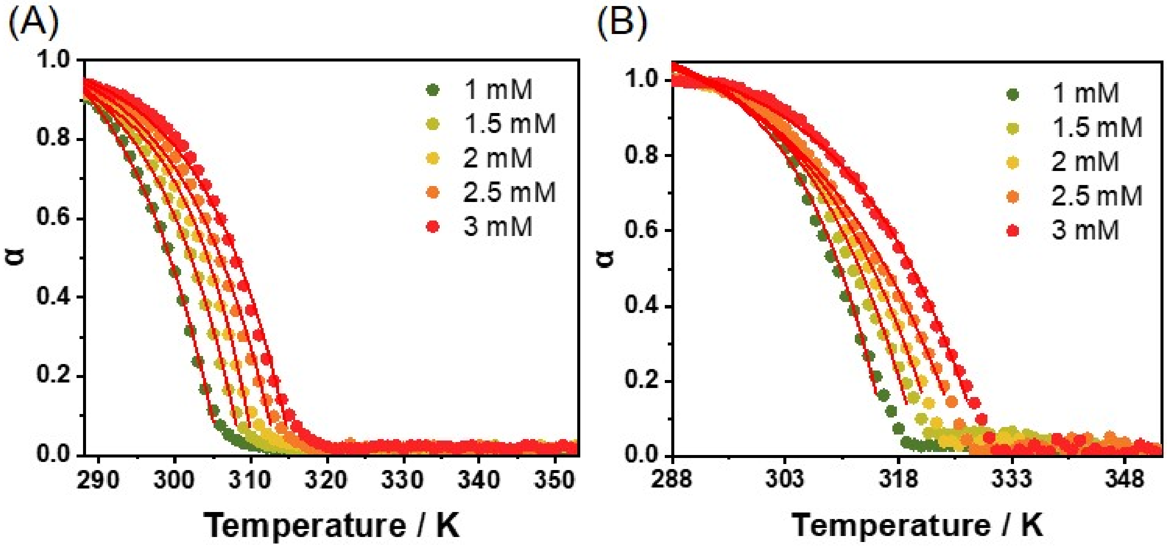

Figure S32. (A) Degree of aggregation (α, at 620 nm) as a function of temperature in different concentrations of Pt-**L**[1] with 1.5 equiv. of $AgNO_3$ in DMSO/H_2O (9:1 v/v). (B) Degree of aggregation (at 650 nm) as a function of temperature in different concentrations of Pt-**L**[1] with 1.5 equiv. of $Fe(BF_4)_2$ in DMSO/H_2O (9:1 v/v).

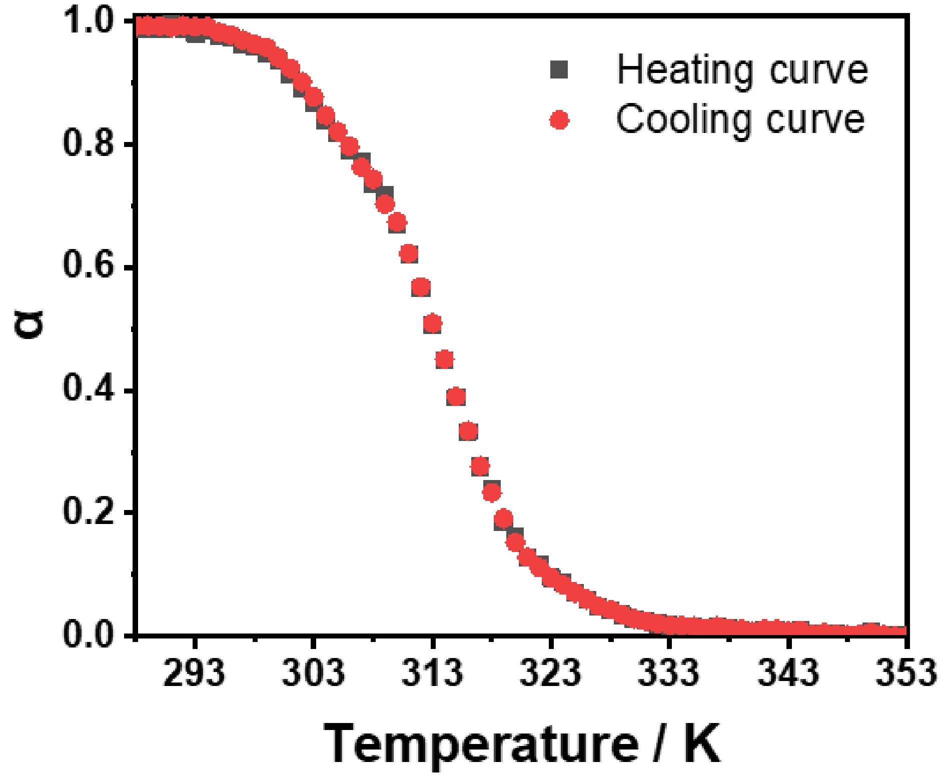

Figure S33. Heating and cooling curves (at 620 nm) of SP-**I** (2.0 mM) in DMSO/H_2O (9:1 v/v).

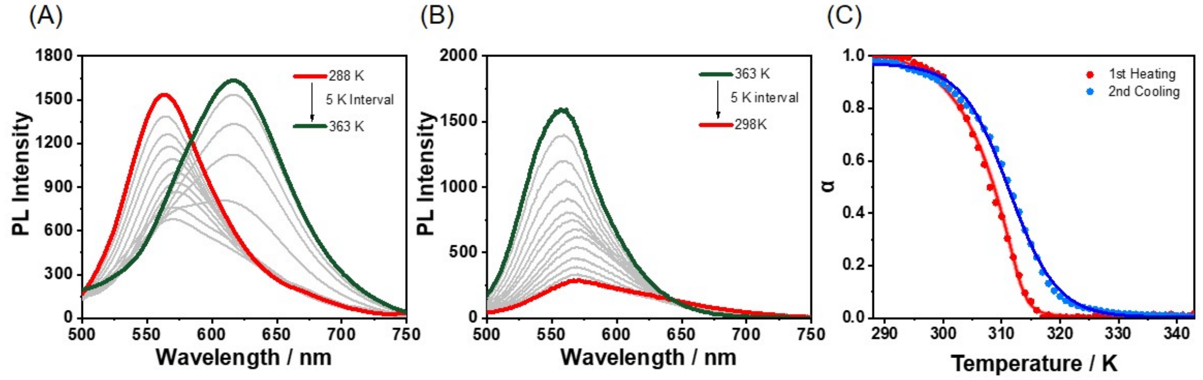

Figure S34. (A) Heating and (B) cooling PL spectra (λ_{ex} = 420 nm) of SP-**III** (2.0 mM) in DMSO/H_2O (9:1 v/v). (C) Heating and cooling curves of SP-**III**. The Heating curve (red line, cooperative model) and cooling curve (blue line, isodesmic model) are fitted by cooperative and isodesmic modes, respectively.

Note: The thermodynamic parameters of SP-**III** is acquired by fitting the melting curves with the one-component mass-balance model. The Gibbs free energy (ΔG_e) and enthalpy (ΔH_e) were calculated to be −19.1 kJmol^{-1} and −141.0 kJmol^{-1}, respectively. Further quantitative analysis yielded a cooperativity parameter σ as the ratio of K_n/K_e, with a value of 1.7×10^{-2}, obtained via curve fitting with Matlab.[15]

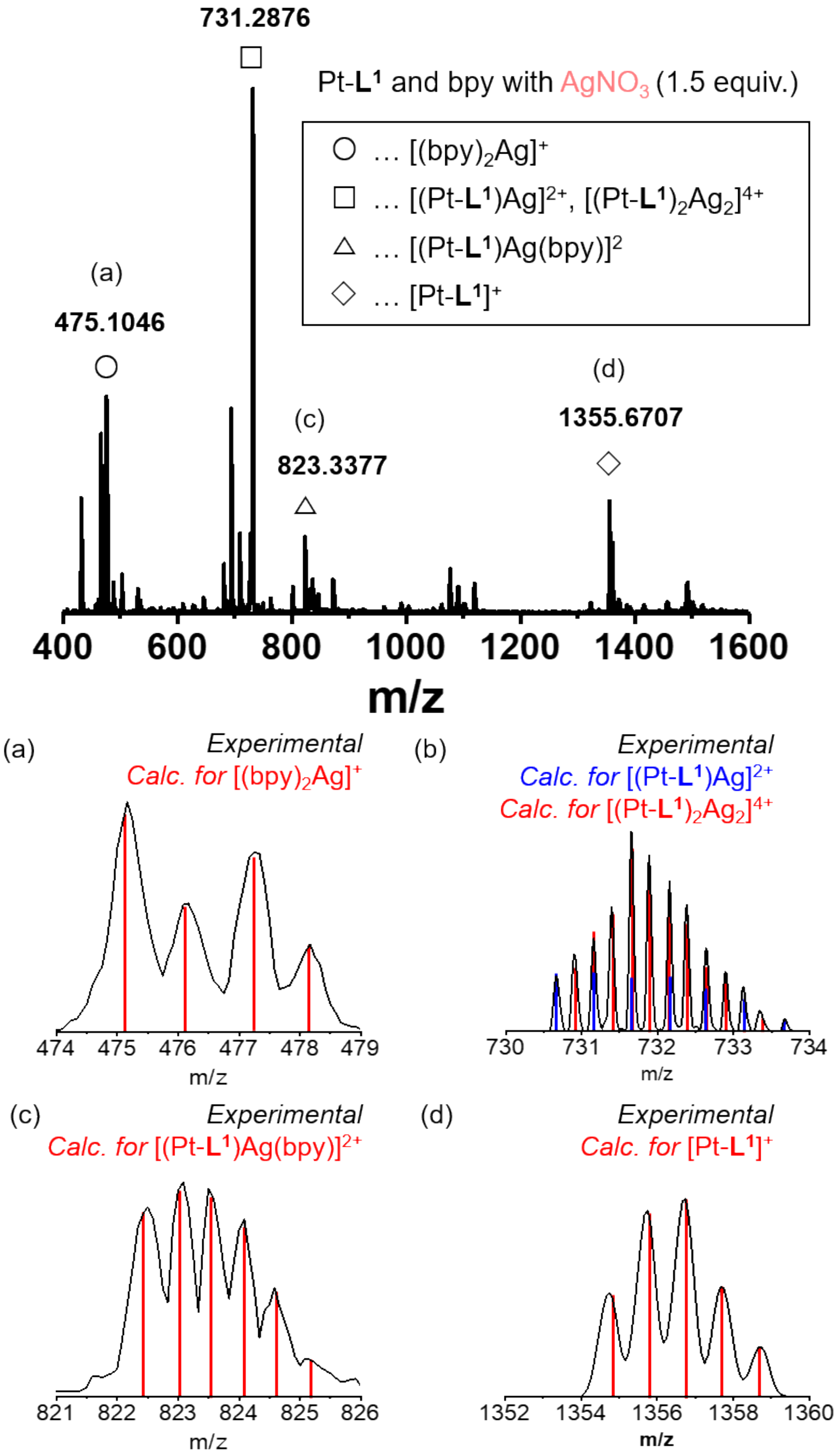

Figure S35. HR-ESI-MS spectrum of a mixture of Pt-**L**[1] (2.0 mM) and $AgNO_3$ (1.5 equiv.) in the presence of bpy (4.0 mM) in DMSO/H_2O (9:1 v/v) after 5 h aging.

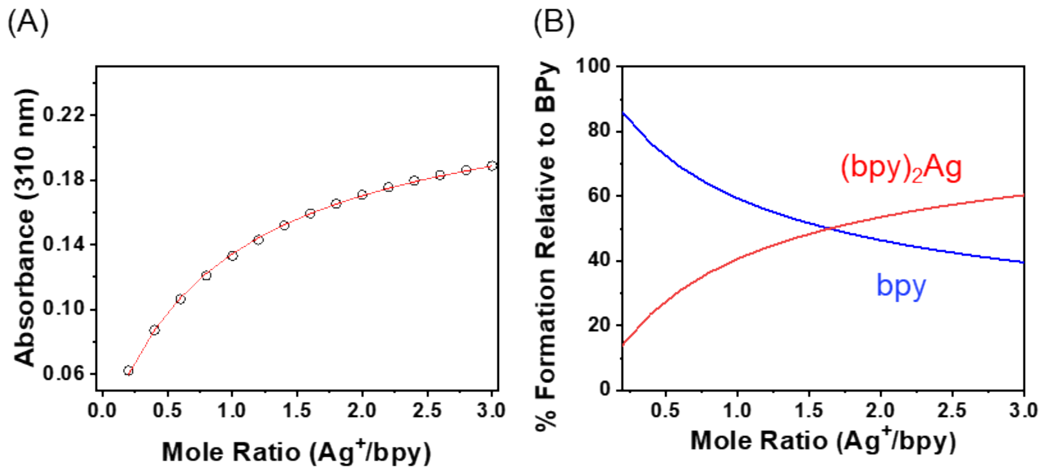

Figure S36. Fitting of the UV-vis titration data to determine the stability constants of the Ag^{+}-bpy complexation with HyperSpec software by employing the multiple binding model including 2:1 ratio: (A) UV-vis absorbance changes (at 310 nm) of bpy (0.3 mM) as a function of the mole ratio (Ag^{+}/bpy, ○: experimental points, solid line: theoretical fit) in DMSO/H_2O (9:1 v/v) at 293 K. (B) Species distribution plots.

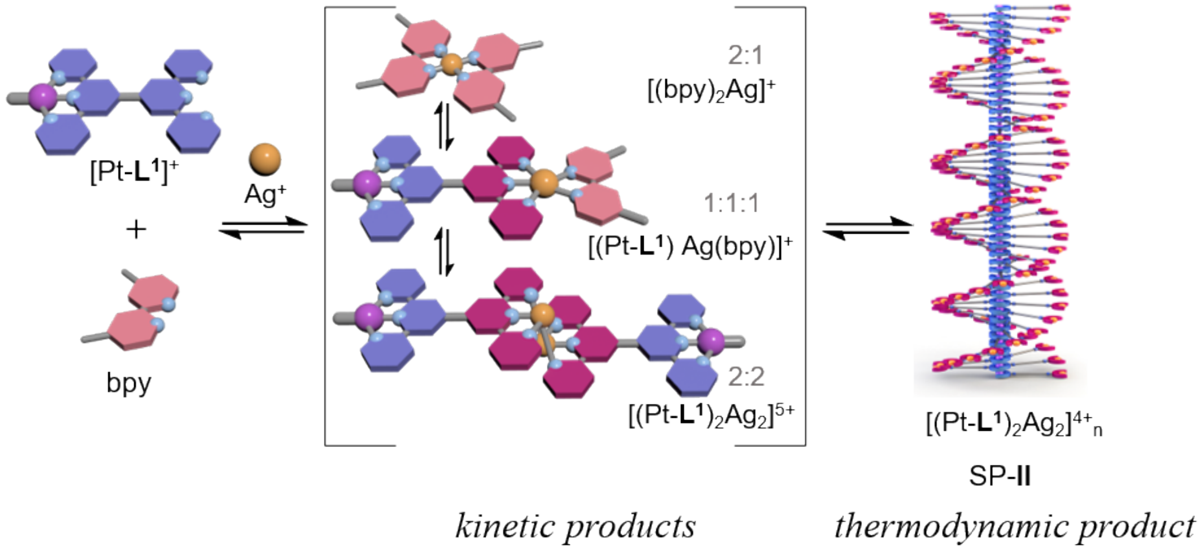

Figure S37. A proposed cooperative mechanism for the chiral supramolecular polymerization of a mixture of Pt-**L**[1] and $AgNO_3$ in the presence of bpy via a nucleation–elongation process to build up SP-**II** as a thermodynamic product.

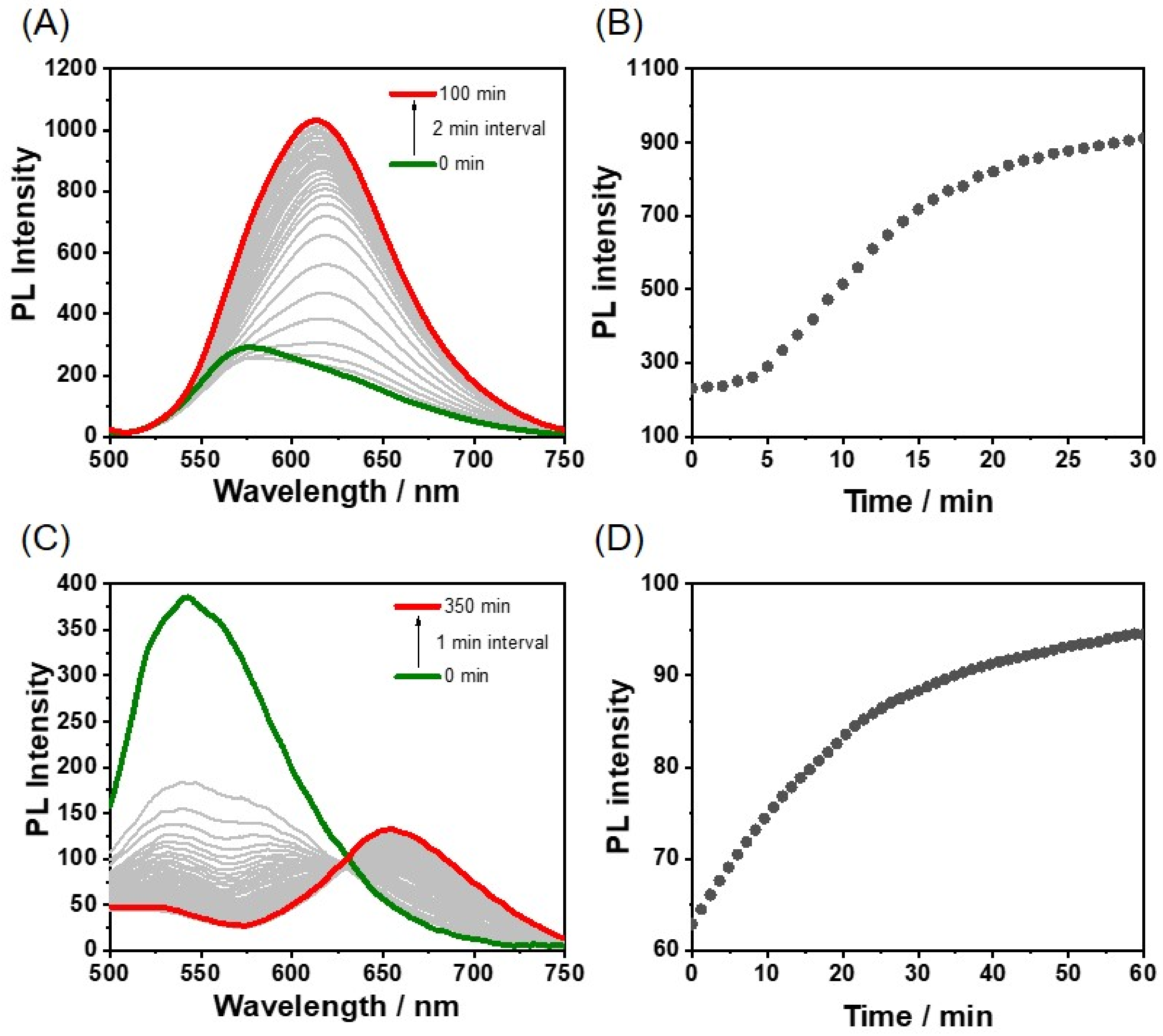

Figure S38. (A and C) Time-dependent PL spectra (λ_{ex} = 420 nm) of a mixed **Pt-L¹** (2.0 mM) and pyridine (6.0 mM) with (A) $AgNO_3$ (1.5 equiv.), (C) $Fe(BF_4)_2$ (1.5 equiv.) in DMSO/H_2O (9:1 v/v) at 293 K. (B and D) Plots of time vs. PL Intensity (620 or 650 nm) of **Pt-L¹** and pyridine (6.0 mM) in the presence of (B) $AgNO_3$ (1.5 equiv.) and (D) $Fe(BF_4)_2$ (1.5 equiv.).

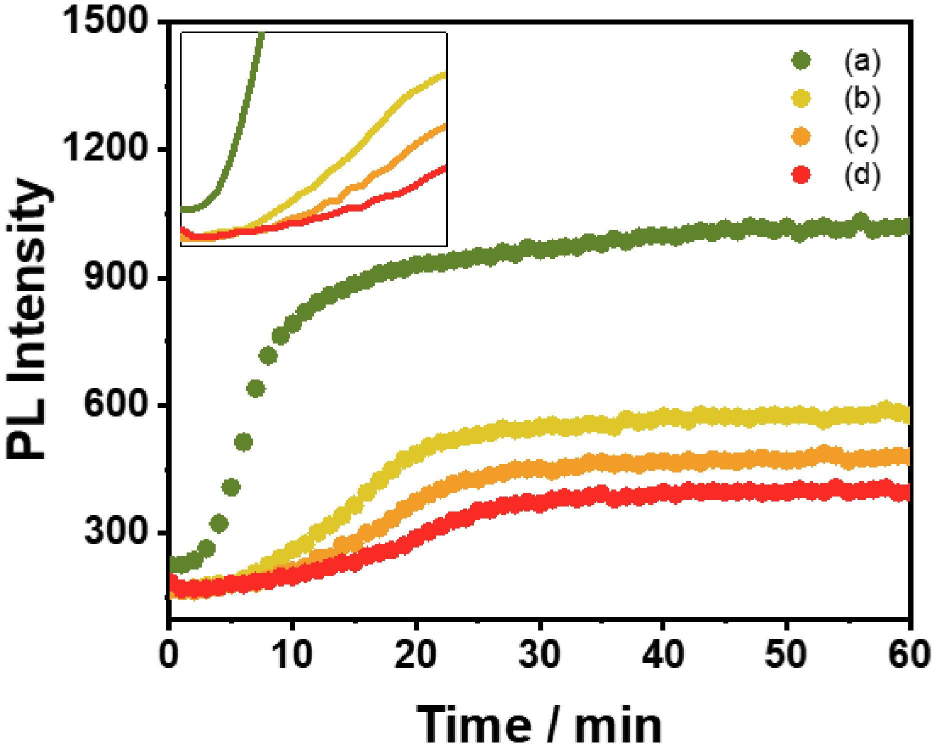

Figure S39. Plots of the PL intensity (at 620 nm. λ_{ex} = 420 nm) vs. times for a mixture of Pt-$\mathbf{L}^1$ (2.0 mM) and $AgNO_3$ (1.5 equiv.) in the presence of DA18C6 (1.0-4.0 mM) in DMSO/H_2O (9:1 v/v) at 293 K. The mole ratios of Pt-$\mathbf{L}^1$/DA18C6; (a)1:0, (b)1:0.5, (c)1:1, and (d)1:2.

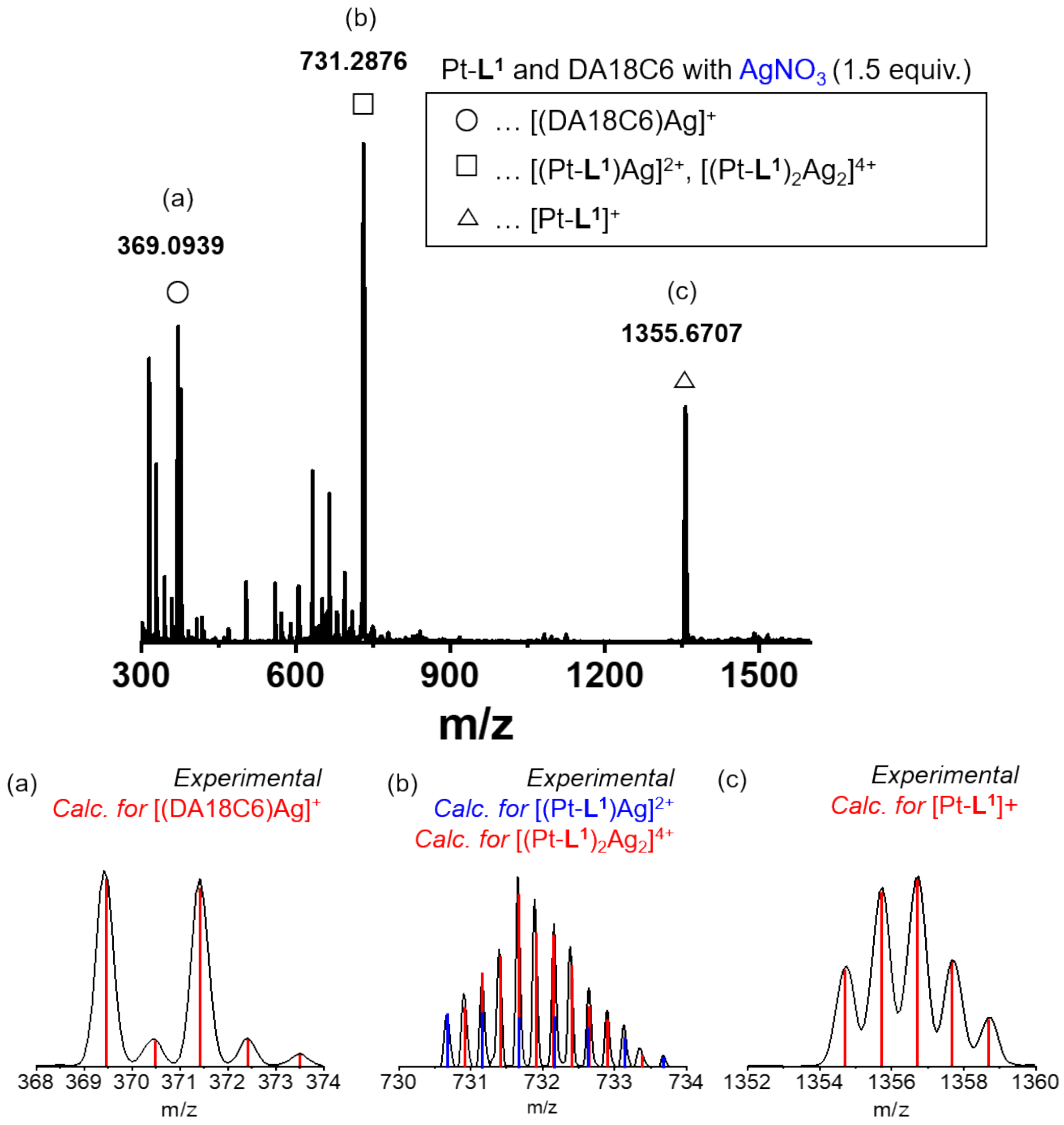

Figure S40. HR-ESI-MS spectrum of a mixture of Pt-**L**[1] (2.0 mM) and $AgNO_3$ (1.5 equiv.) in the presence of DA18C6 (4.0 mM) in DMSO/H_2O (9:1 v/v) after 5 h aging.

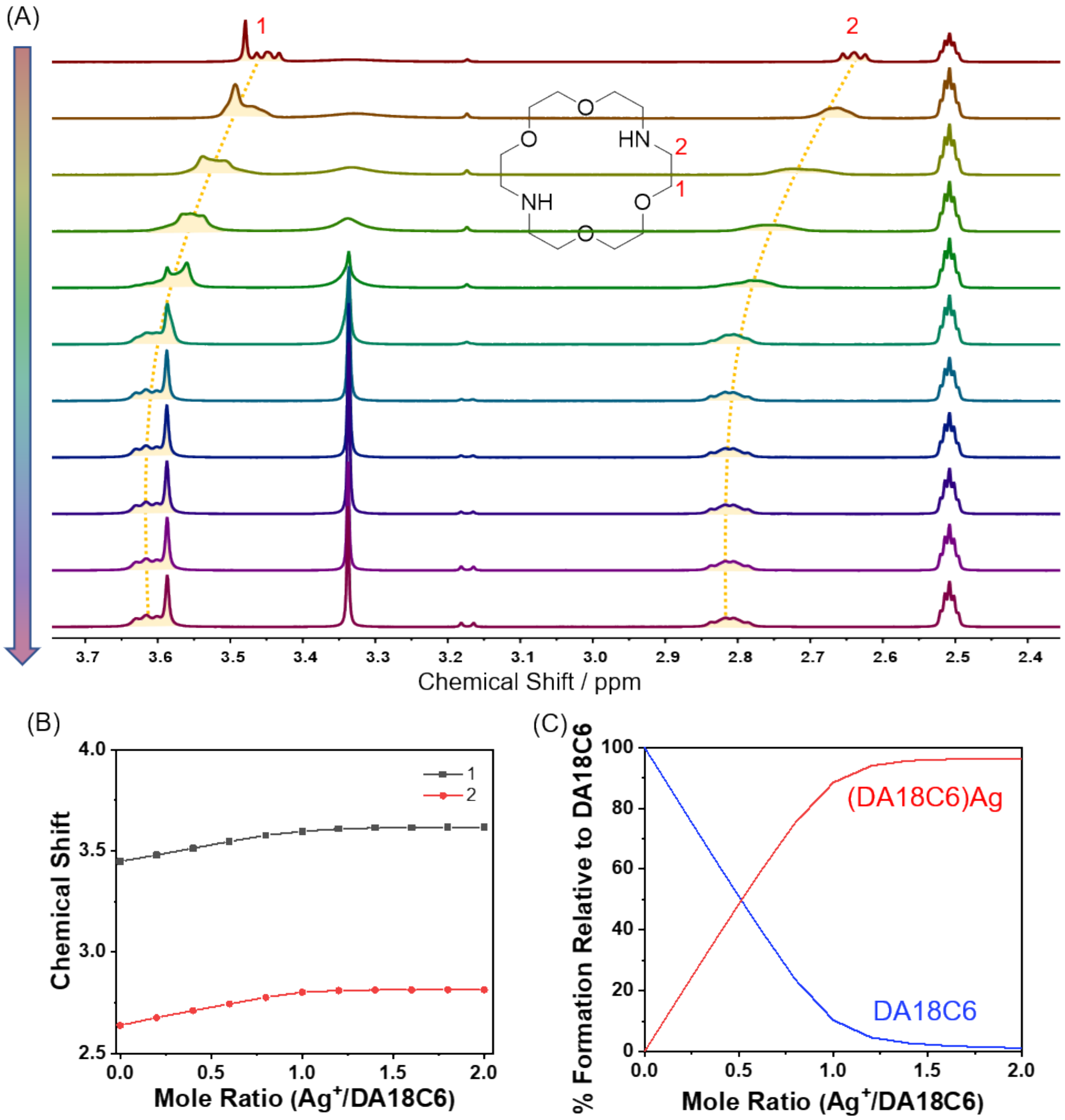

Figure S41. (A) ^1H NMR (300 MHz) titration of DA18C6 (5.0 mM) with $AgNO_3$ in DMSO-d_6/D_2O (9:1 v/v) at 298 K. (B) Titration curves for the protons H_1 and H_2 in DA18C6. (C) Species distribution plots (fitting output from HyperNMR software).

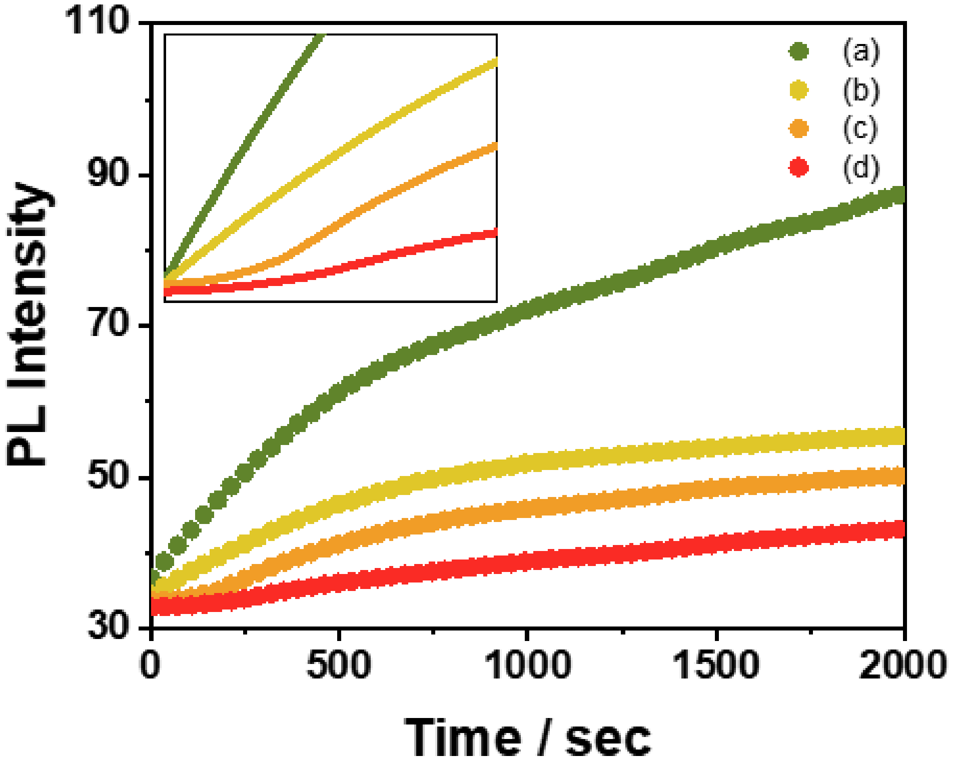

Figure S42. Plots of the PL intensity (at 620 nm, λ_{ex} = 420 nm) vs. times for a mixture of Pt-$\mathbf{L}^1$ (2.0 mM) and $Fe(BF_4)_2$ (1.5 equiv.) in the presence of DA18C6 (2.0-6.0 mM) in DMSO/H_2O (9:1 v/v) at 293 K. The mole ratios of Pt-$\mathbf{L}^1$/DA18C6; (a)1:0, (b)1:1, (c)1:2, and (d)1:3.

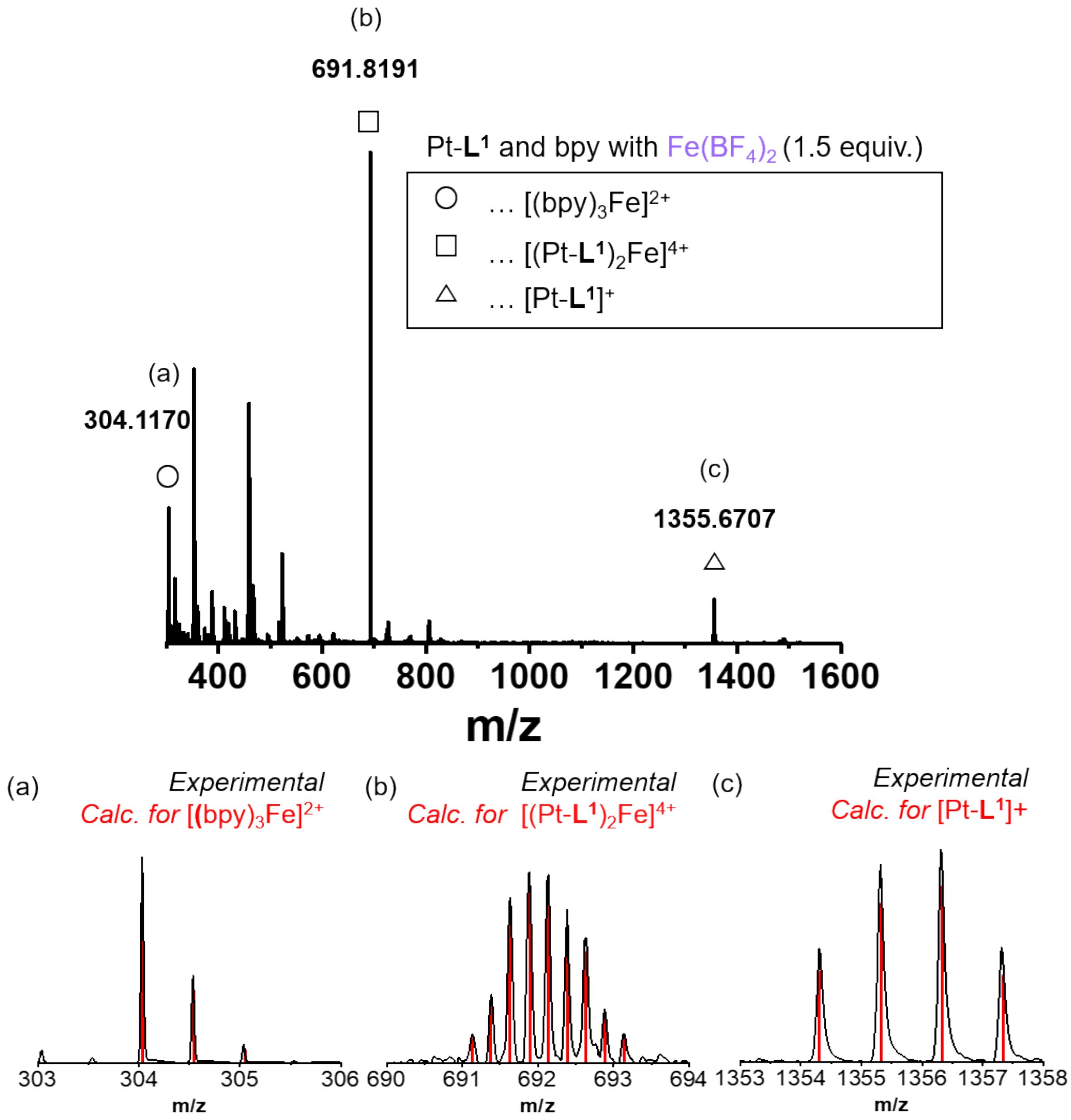

Figure S43. HR-ESI-MS spectrum of a mixture of Pt-**L**[1] (2.0 mM) and bpy (4.0 mM) in the presence of $Fe(BF_4)_2$ (1.5 equiv.) in DMSO/H_2O (9:1 v/v) after 5 h aging.

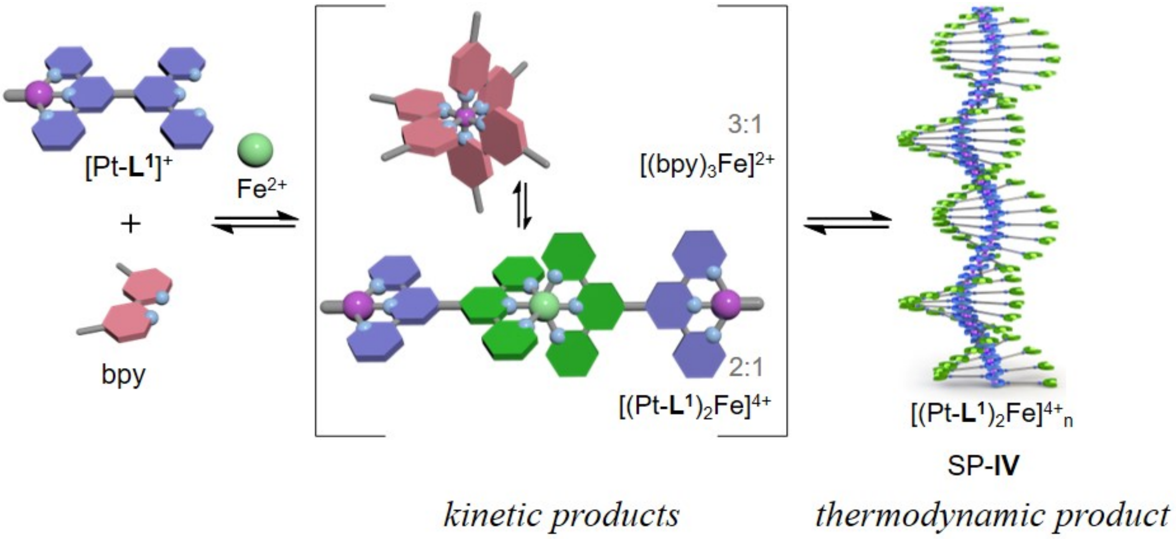

Figure S44. A proposed cooperative mechanism for the chiral supramolecular polymerization of a mixture of Pt-**L**[1] and $Fe(BF_4)_2$ in the presence of bpy via a nucleation–elongation process to build up SP-**IV** as a thermodynamic product.

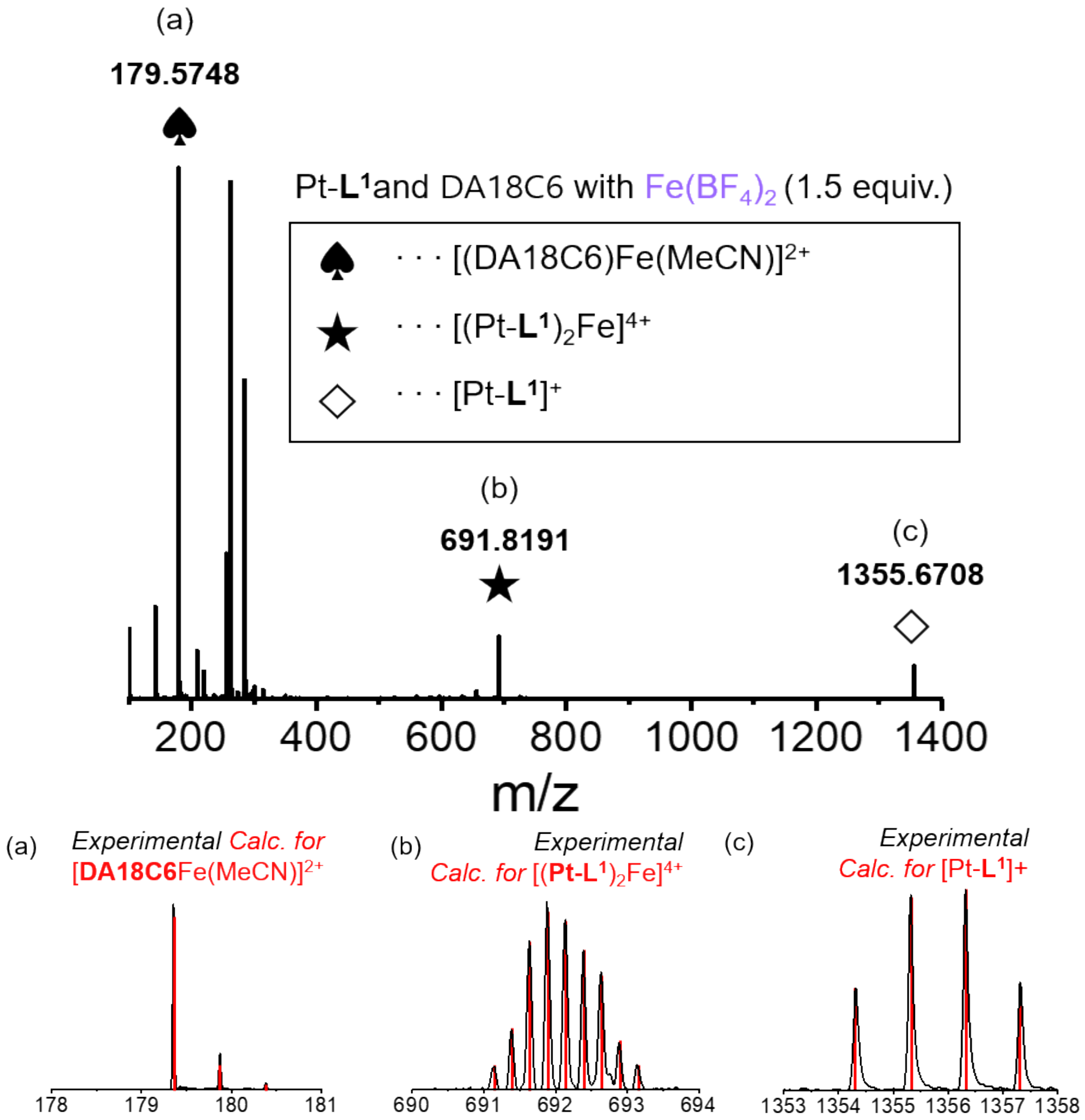

Figure S45. HR-ESI-MS spectrum of a mixture of Pt-**L**[1] (2.0 mM) and DA18C6 (4.0 mM) in the presence of $Fe(BF_4)_2$ (1.5 equiv.) in DMSO/H_2O (9:1 v/v) after 5 h aging.

Note: MeCN was used to dilute the sample for MS observation.

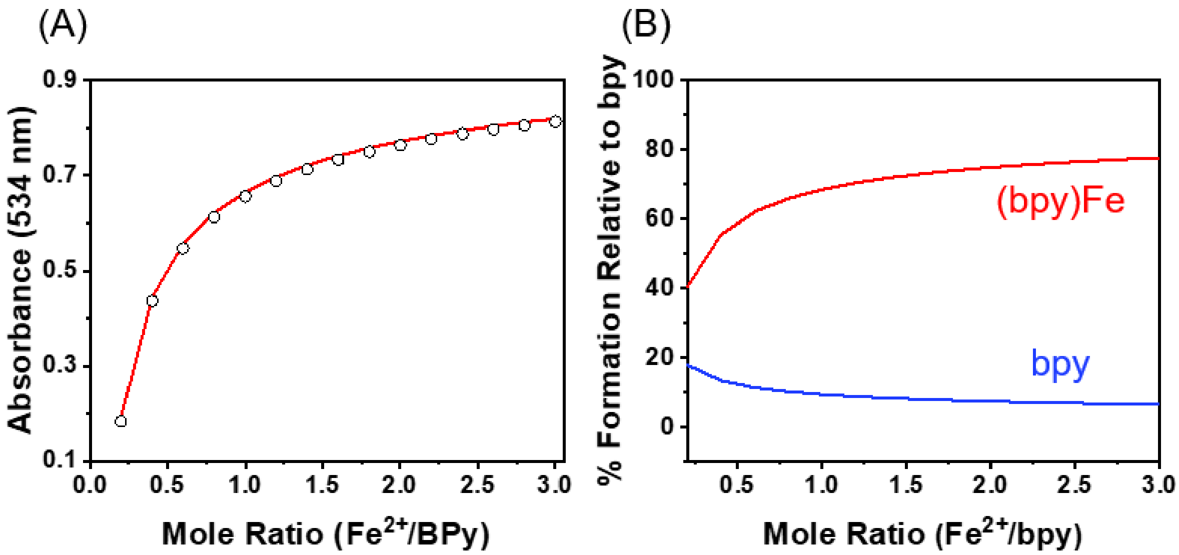

Figure S46. Fitting of the UV-vis titration data to determine the stability constants of the Fe^{2+}-bpy complexation with HyperSpec software by employing the multiple binding model including 3:1 ratio: (A) UV-vis absorbance changes (at 534 nm) of bpy (0.3 mM) as a function of the mole ratio (Fe^{2+}/bpy, ○: experimental points, solid line: theoretical fit)) in DMSO/H_2O (9:1 v/v) at 293 K. (B) Species distribution plots.

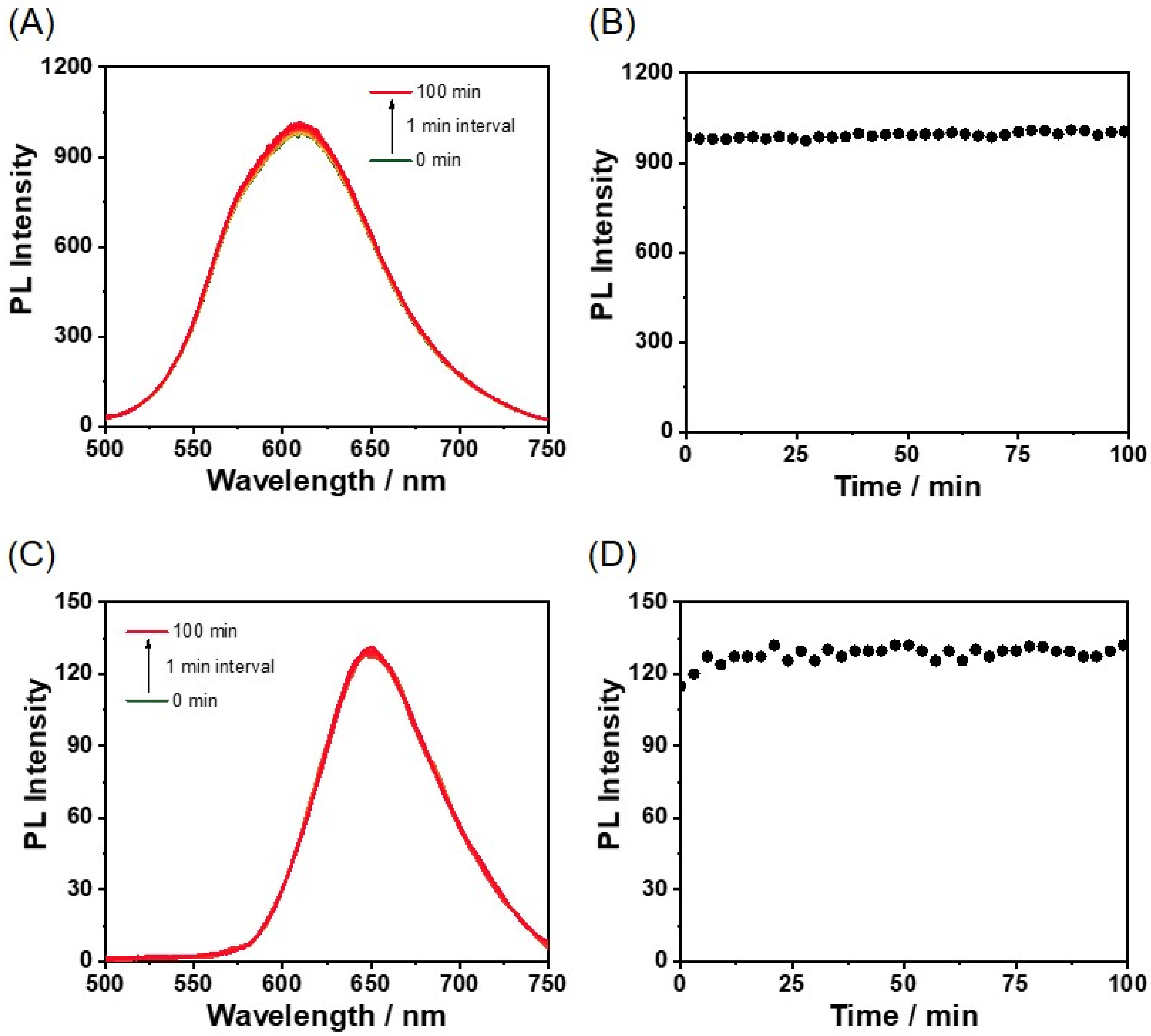

Figure S47. Time-dependent PL spectra (λ_{ex} = 420 nm) of (A) SP-**II** (2.0 mM) and (C) SP-**IV** (2.0 mM) upon the addition of bpy (6.0 mM) in DMSO/H_2O (9:1 v/v) at 293 K. Plots of time vs. PL Intensity (at 620 or 650 nm) for (B) SP-**II** and (D) SP-**IV** with bpy.

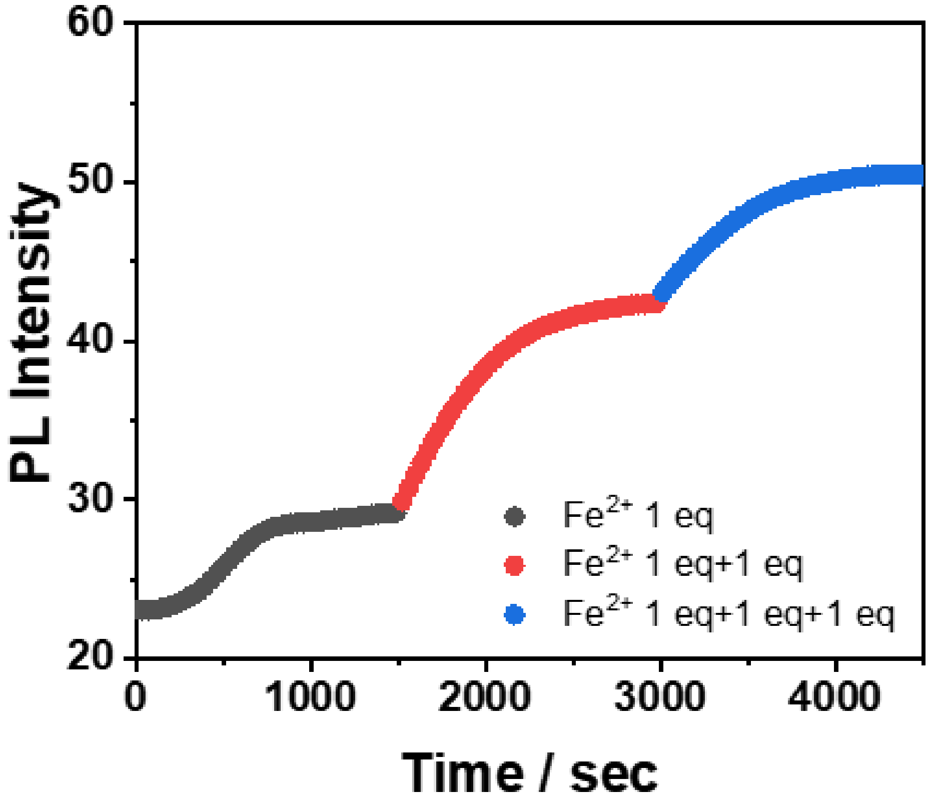

Figure S48. Plot of PL intensity (at 650 nm, λ_{ex} = 420 nm) vs. times during the stepwise addition of Fe^{2+} to a mixture of Pt-**L**[1] (2.0 mM) and bpy (4.0 mM) in DMSO/H_2O (9:1 v/v) at 293 K. Each 1.0 equiv. of $Fe(BF_4)_2$ was added three times (totally 3.0 equiv.).

4. Analytical Data

4.1 ^{1}H-NMR and ^{13}C-NMR Spectroscopy

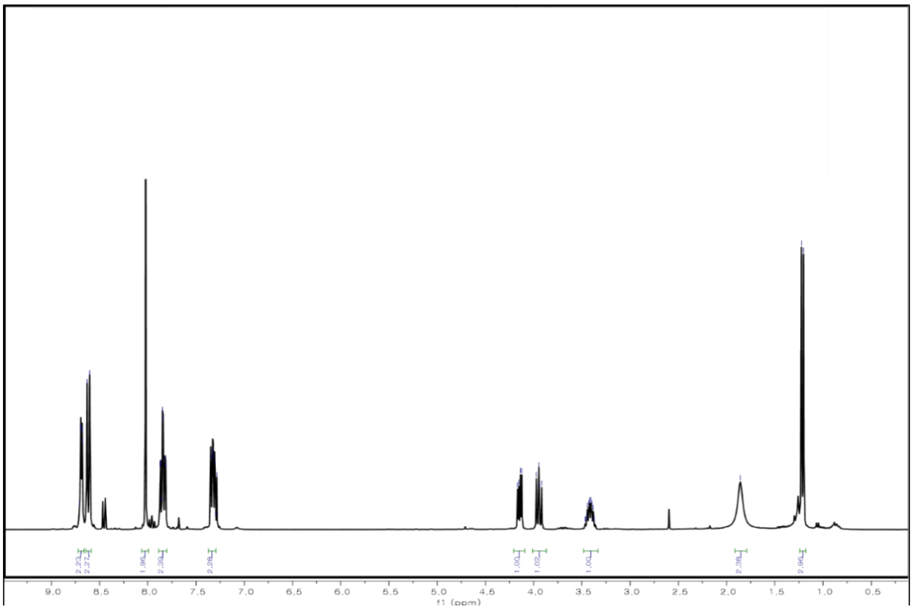

Figure S49. ^{1}H NMR spectrum (300 MHz) of *R*-**L**^{2} in $CDCl_3$ at 298 K.

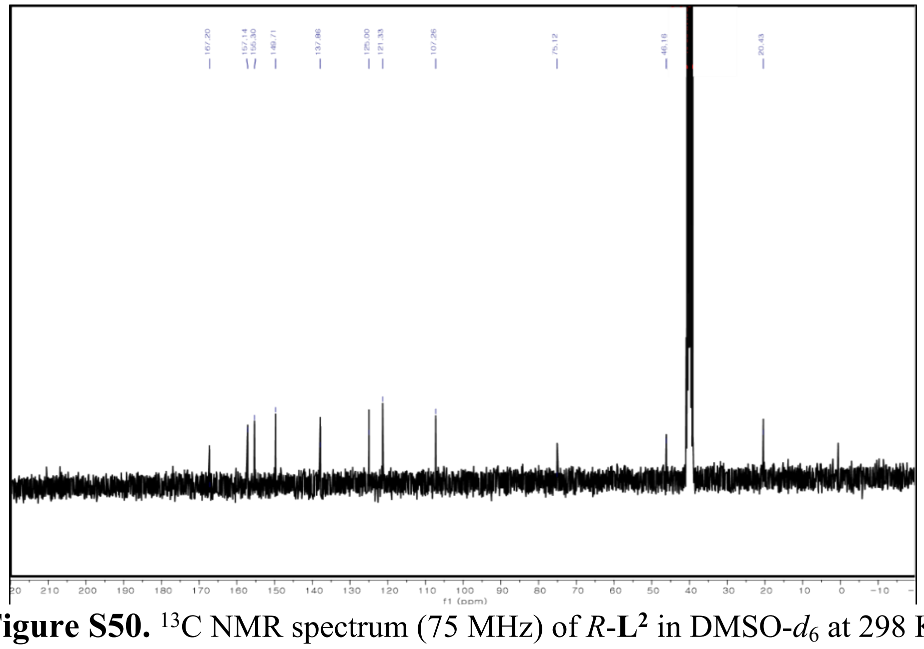

Figure S50. ^{13}C NMR spectrum (75 MHz) of *R*-**L**^{2} in DMSO-d_6 at 298 K.

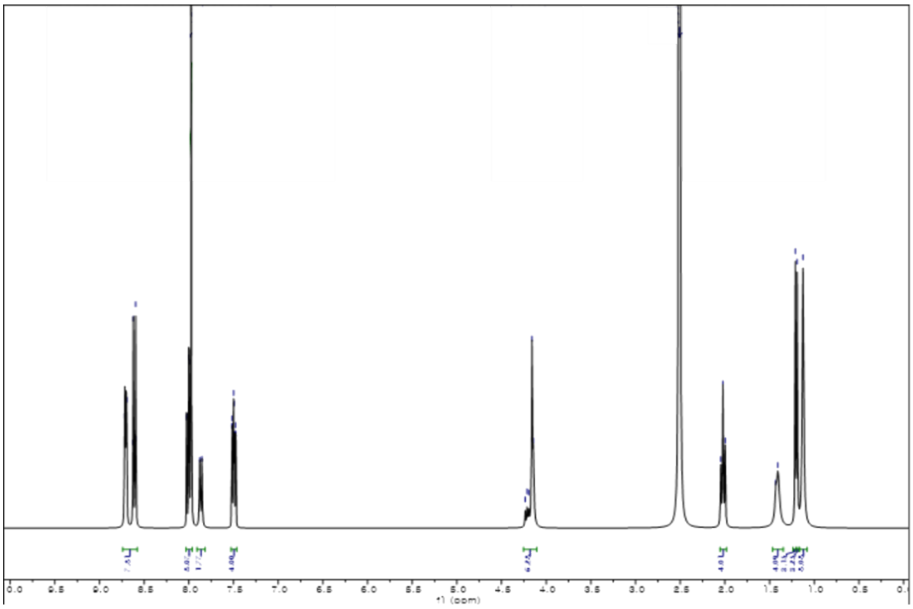

Figure S51. ^{1}H NMR spectrum (300 MHz) of *R*-**L^{1}** in DMSO-d_6 at 298 K

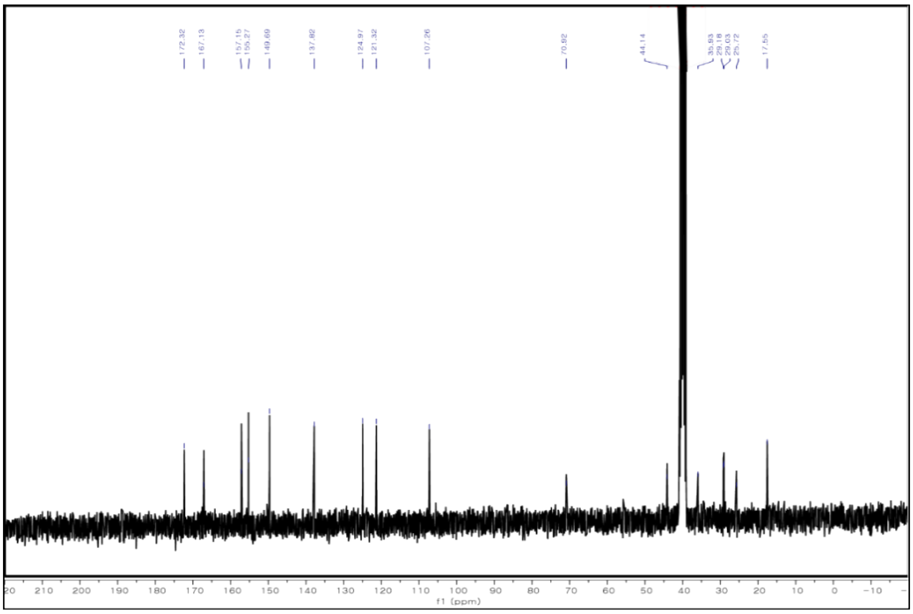

Figure S52. ^{13}C NMR spectrum (75 MHz) of *R*-**L^{1}** in DMSO-d_6 at 298 K.

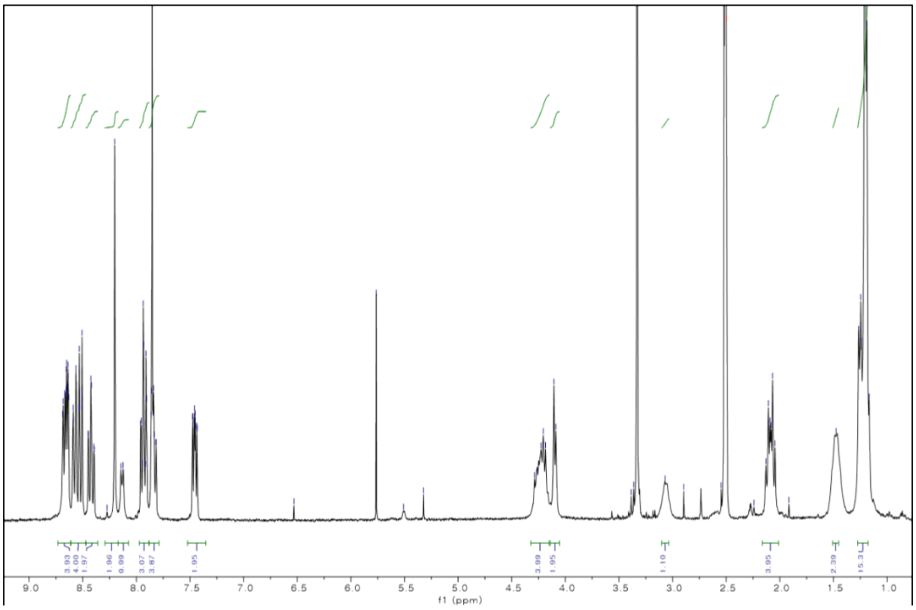

Figure S53. ^{1}H NMR spectrum (300 MHz) of PtCl-$\mathbf{L^2}$ in DMSO-d_6 at 298 K.

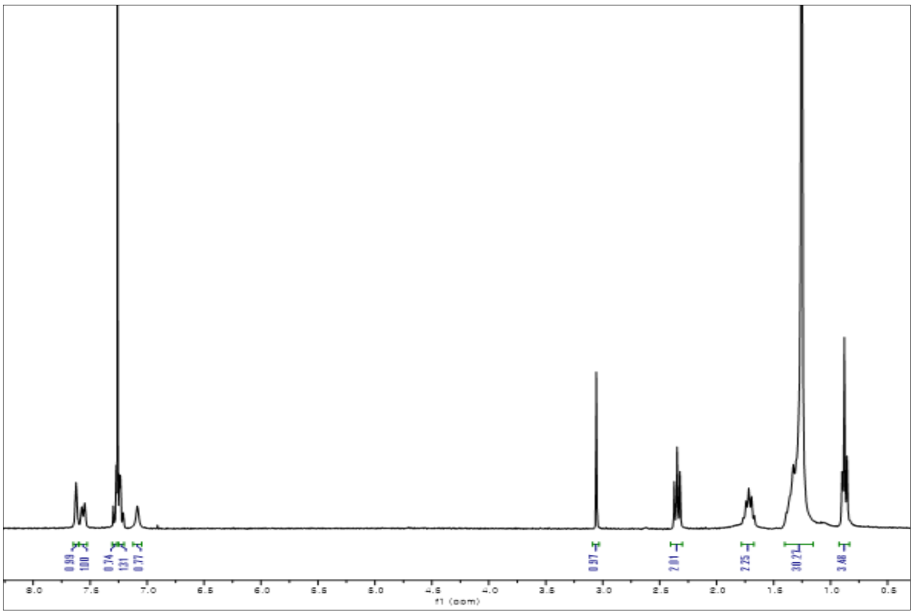

Figure S54. ^{1}H NMR spectrum (300 MHz) of **R-1** in $CDCl_3$ at 298 K.

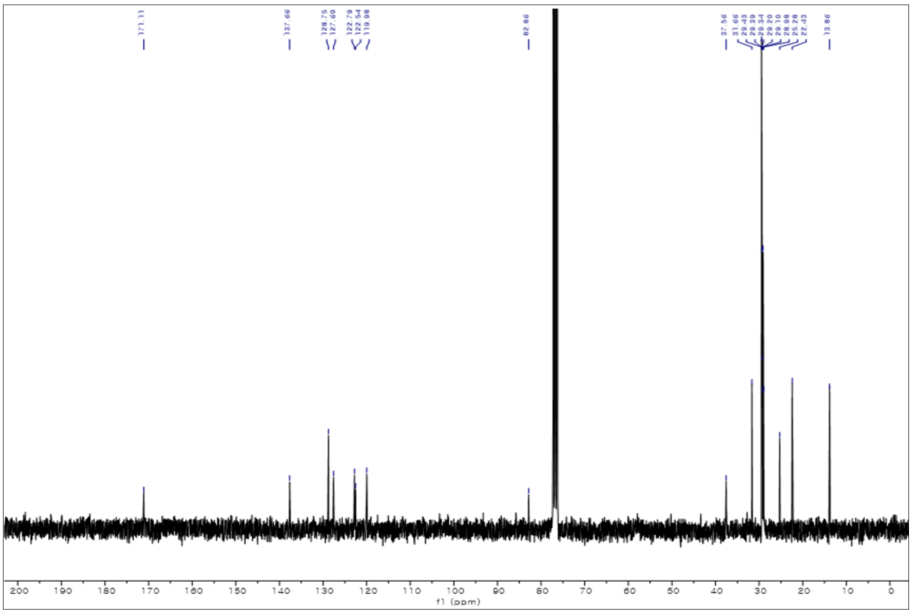

Figure S55. ^{13}C NMR spectrum (75 MHz) of **R-1** in $CDCl_3$ at 298 K.

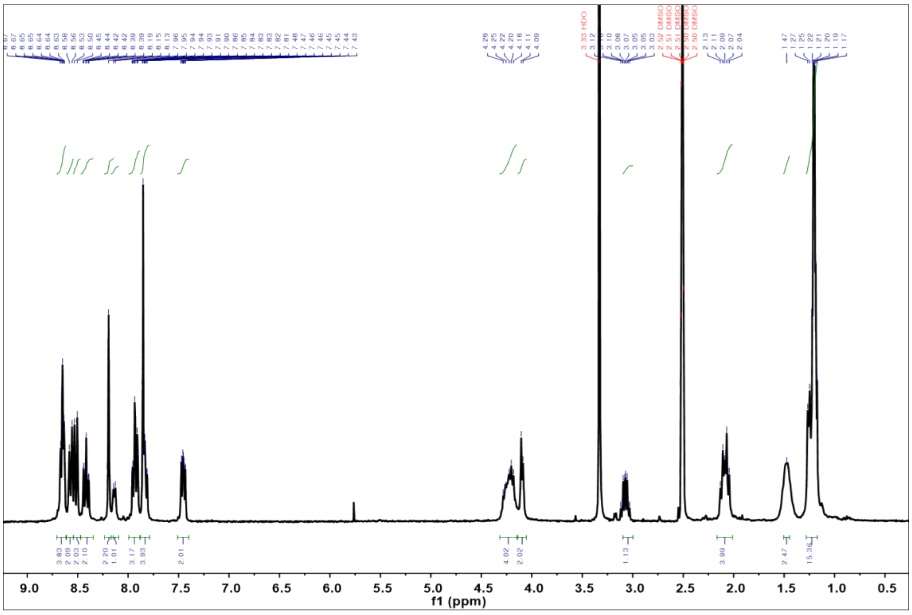

Figure S56. ^{1}H NMR spectrum (300 MHz) of Pt-$\mathbf{L^3}$ in DMSO-d_6 at 298 K.

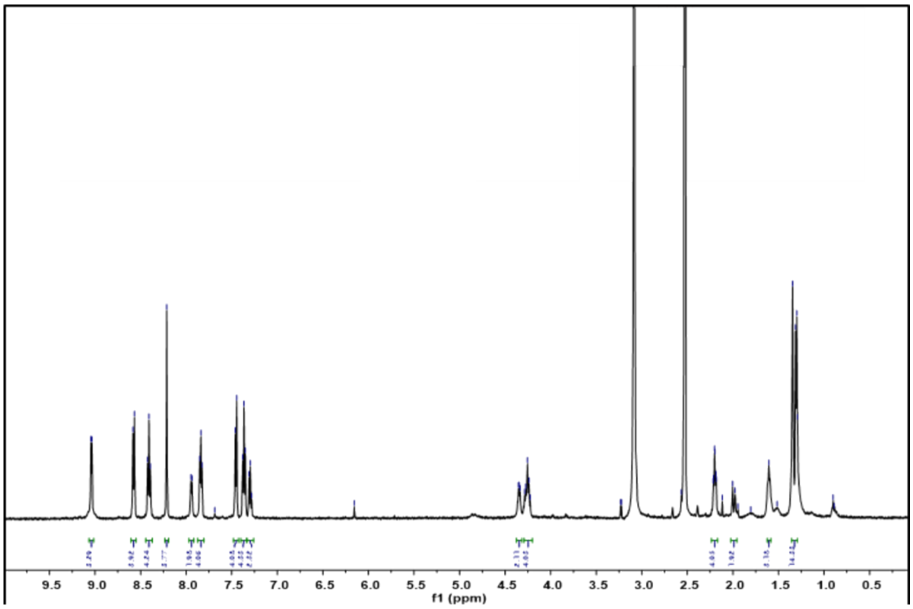

Figure S57. ^{1}H NMR spectrum (300 MHz) of Pt-$\mathbf{L^2}$ in DMSO-d_6 at 353 K.

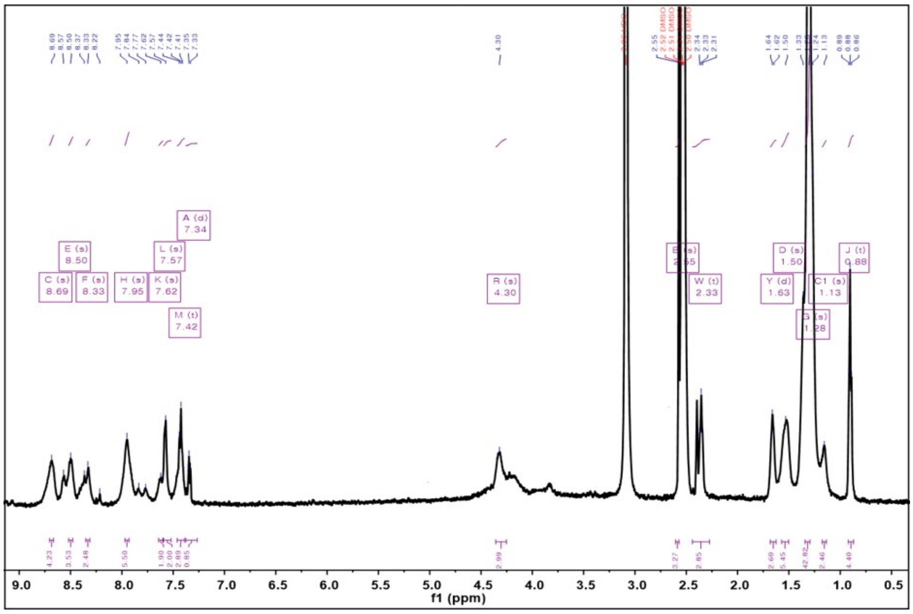

Figure S58. ^{1}H NMR spectrum (500 MHz) of Pt-$\mathbf{L^1}$ in DMSO-d_6 at 353 K.

4.2 HR and ESI Mass Spectrometry

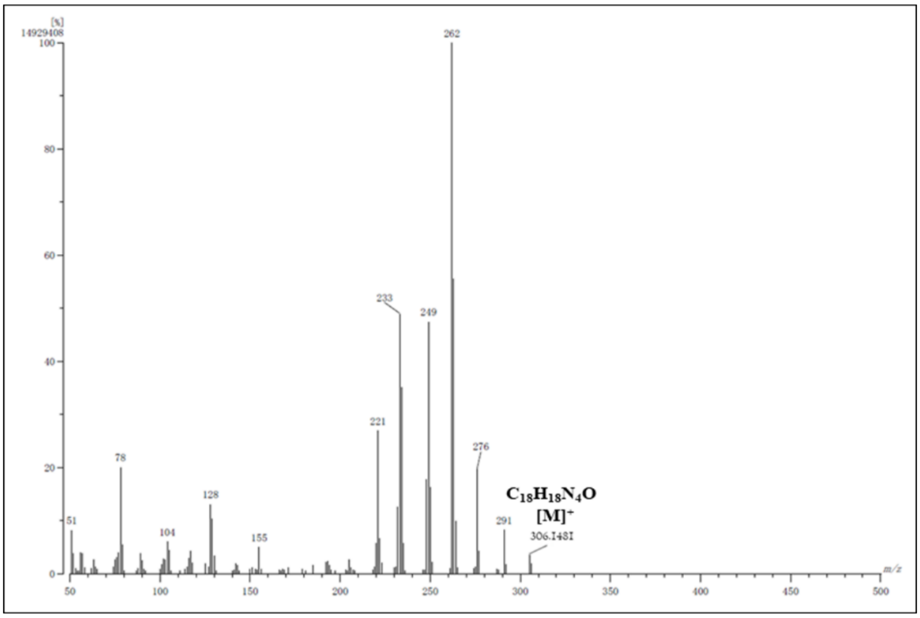

Figure S59. HR-EI-MS spectrum of *R*-$\mathbf{L}^2$ in DCM.

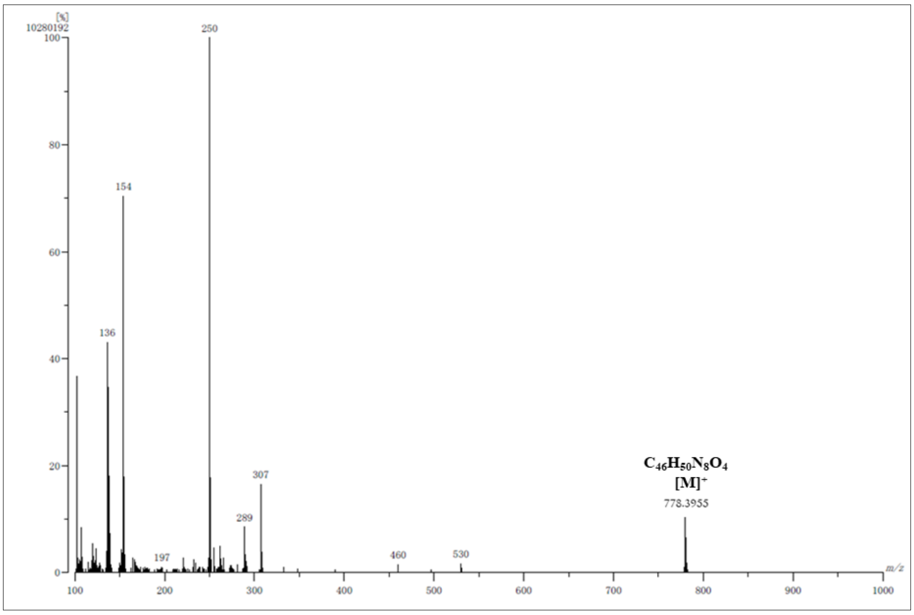

Figure S60. HR-FAB-MS spectrum of *R*-$\mathbf{L}^1$ in DCM.

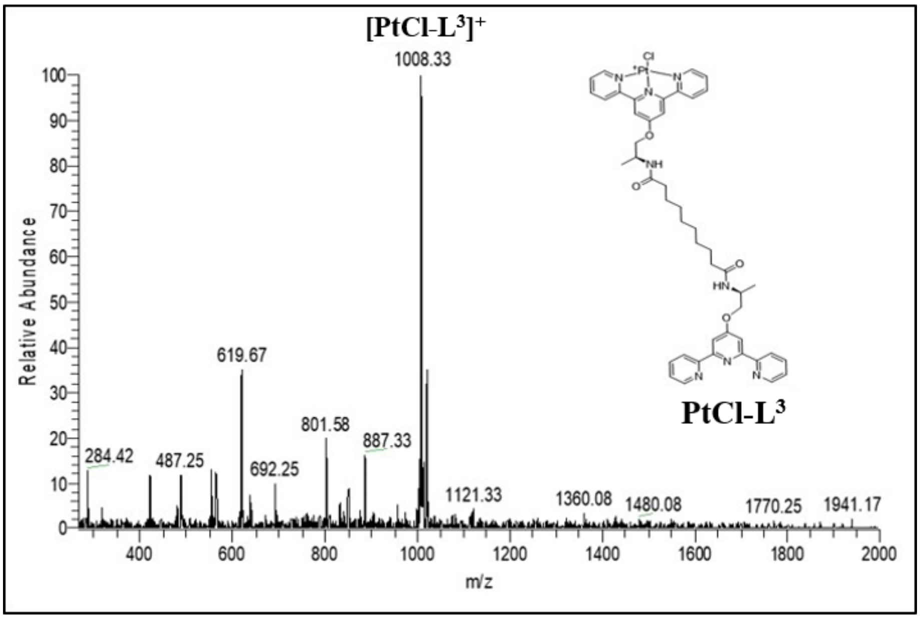

Figure S61. ESI mass spectrum of PtCl-$\mathbf{L^2}$ in H_2O.

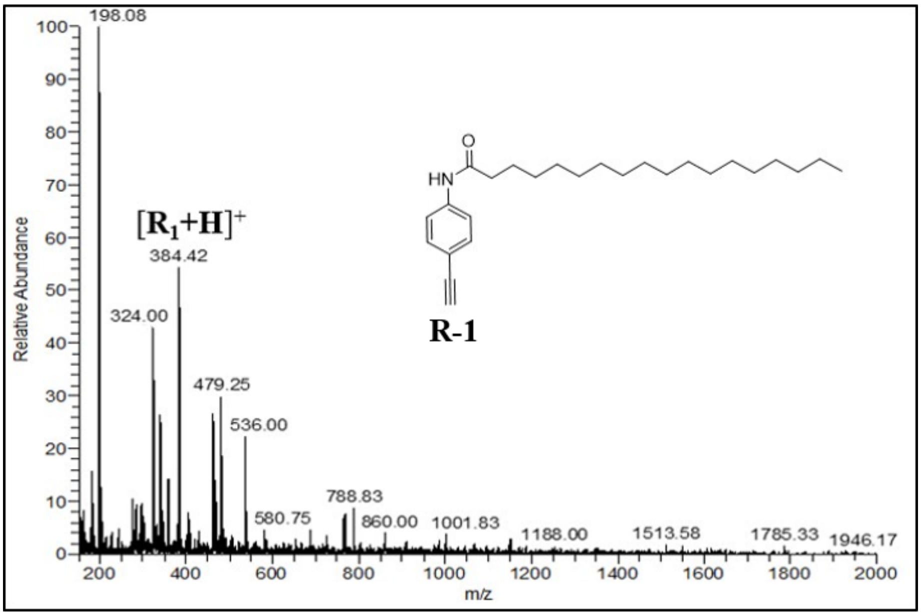

Figure S62. ESI mass spectrum of **R-1** in MeOH.

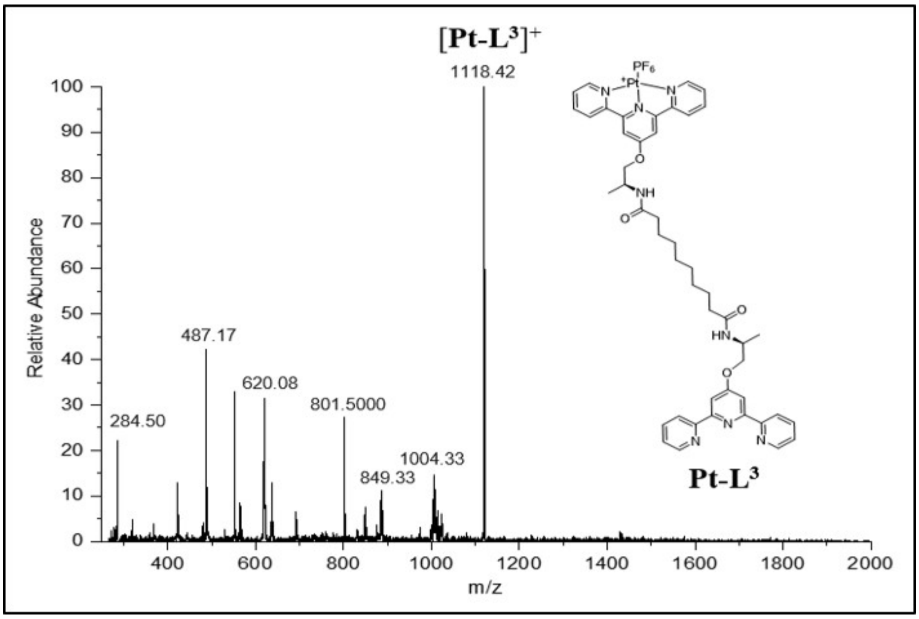

Figure S63. ESI mass spectrum of Pt-$\mathbf{L^3}$ in MeOH.

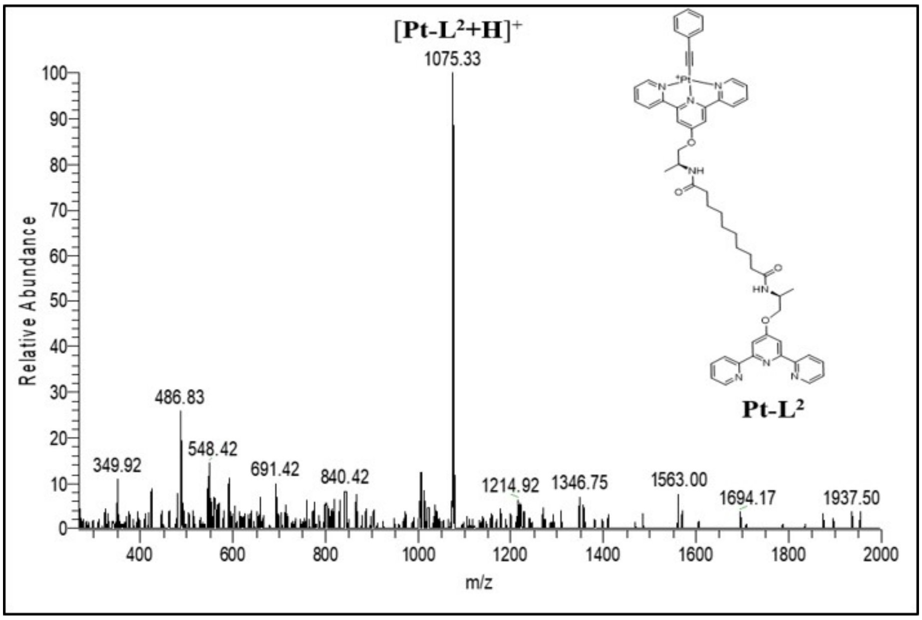

Figure S64. ESI mass spectrum of Pt-$\mathbf{L^2}$ in MeOH.

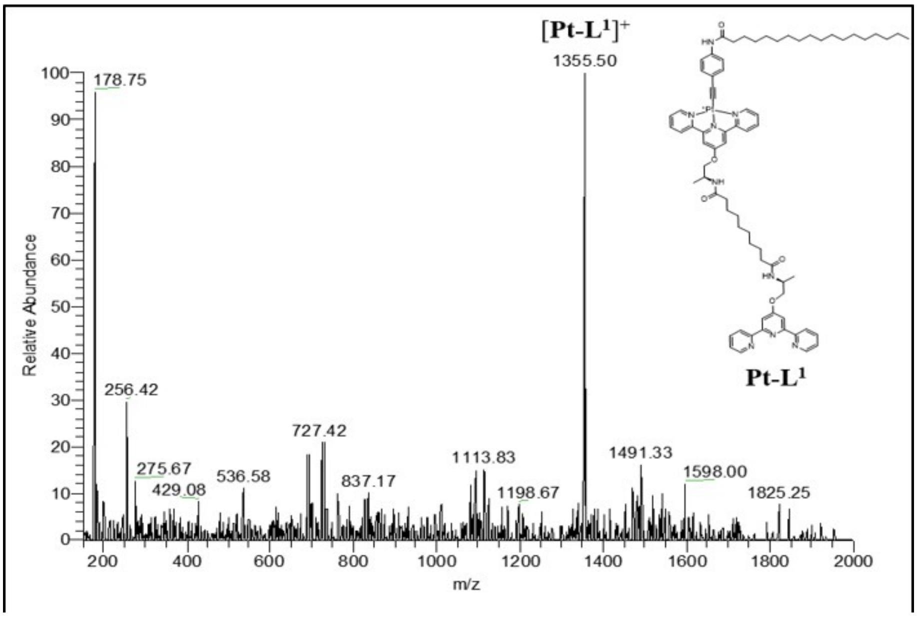

Figure S65. ESI mass spectrum of Pt-**L1** in MeOH.

5. Supplementary References

(1) Smulders, M. M. J.; Nieuwenhuizen, M. M. L.; de Greef, T. F. A.; van der Schoot, P.; Schenning, A. P. H. J.; Meijer, E. W., How to Distinguish Isodesmic from Cooperative Supramolecular Polymerisation. *Chem. Eur. J.* **2010,** *16*, 362-367.
(2) Jonkheijm, P.; van der Schoot, P.; Schenning, A. P. H. J.; Meijer, E. W., Probing the Solvent-Assisted Nucleation Pathway in Chemical Self-Assembly. *Science* **2006,** *313*, 80-83.
(3) Markvoort, A. J.; ten Eikelder, H. M. M.; Hilbers, P. A. J.; de Greef, T. F. A.; Meijer, E. W., Theoretical models of nonlinear effects in two-component cooperative supramolecular copolymerizations. *Nature communications* **2011,** *2*, 509.
(4) Wehner, M.; Röhr, M. I. S.; Bühler, M.; Stepanenko, V.; Wagner, W.; Würthner, F., Supramolecular Polymorphism in One-Dimensional Self-Assembly by Kinetic Pathway Control. *J. Am. Chem. Soc.* **2019,** *141*, 6092-6107.
(5) Dhiman, S.; Sarkar, A.; George, S. J., Bioinspired temporal supramolecular polymerization. *RSC Adv.* **2018,** *8*, 18913-18925.
(6) De Greef, T. F. A.; Smulders, M. M. J.; Wolffs, M.; Schenning, A. P. H. J.; Sijbesma, R. P.; Meijer, E. W., Supramolecular Polymerization. *Chem. Rev.* **2009**, *109*, 5687-5754.
(7) DOI: http://www.hyperquad.co.uk/HypSpec2014.htm.
(8) P. Gans, A. Sabatini and A. Vacca, *Talanta*, **1996**, 43, 1739-1753.
(9) H. Ju, T. Abe, Y. Takahashi, Y. Tsuruoka, A. Otsuka, E. Lee, M. Ikeda, S. Kuwahara and Y. Habata, *Inorg. Chem.*, **2021**, 60, 1738-1745.
(10) M. J. Frisch, G. W. T., H. B. Schlegel, G. E. Scuseria, M. A. Robb, J. R. Cheeseman, G. Scalmani, V. Barone, B. Mennucci, G. A. Petersson, H. Nakatsuji, M. Caricato, X. Li, H. P. Hratchian, A. F. Izmaylov, J. Bloino, G. Zheng, J. L. Sonnenberg, M. Hada, M. Ehara, K. Toyota, R. Fukuda, J. Hasegawa, M. Ishida, T. Nakajima, Y. Honda, O. Kitao, H. Nakai, T. Vreven, J. A. Montgomery, Jr., J. E. Peralta, F. Ogliaro, M. Bearpark, J. J. Heyd, E. Brothers, K. N. Kudin, V. N. Staroverov, R. Kobayashi, J. Normand, K. Raghavachari, A. Rendell, J. C. Burant, S. S. Iyengar, J. Tomasi, M. Cossi, N. Rega, J. M. Millam, M. Klene, J. E. Knox, J. B. Cross, V. Bakken, C. Adamo, J. Jaramillo, R. Gomperts, R. E. Stratmann, O. Yazyev, A. J. Austin, R. Cammi, C. Pomelli, J. W. Ochterski, R. L. Martin, K. Morokuma, V. G. Zakrzewski, G. A. Voth, P. Salvador, J. J. Dannenberg, S. Dapprich, A. D. Daniels, Ö. Farkas, J. B. Foresman, J. V. Ortiz, J. Cioslowski, and D. J. Fox Gaussian 09 Gaussian 09 Wallingford, CT, **2009**.
(11) J. P. Perdew, Phys. Rev. B, **1986**, *33*, 8822-8824.
(12) F. Weigend, Phys. Chem. Chem. Phys., **2006**, *8*, 1057-1065.
(13) F. Weigend and R. Ahlrichs, Phys. Chem. Chem. Phys., **2005**, *7*, 3297-3305.
(14) C. Kim, K. Y. Kim, J. H. Lee, J. Ahn, K. Sakurai, S. S. Lee and J. H. Jung, ACS Appl. Mater. Interfaces, **2017**, *9*, 3799-3807.
(15) Eikelder, ten; H. M. M.; Markvoort, A. J.; de Greef, T. F. A.; Hilbers, P. A. J. An equilibrium model for chiral amplification in supramolecular polymers. J. Phys. Chem. B. **2012**, *116*, 5291-5301.
